# PB01 suppresses radio-resistance by regulating ATR signaling in human non-small-cell lung cancer cells

**DOI:** 10.1038/s41598-021-91716-z

**Published:** 2021-06-08

**Authors:** Tae Woo Kim, Da-Won Hong, Sung Hee Hong

**Affiliations:** Division of Radiation Biomedical Research, Korea Institute of Radiological and Medical Sciences, Seoul, 139-706 Korea

**Keywords:** Biochemistry, Cancer, Cell biology, Chemical biology, Drug discovery, Molecular biology, Molecular medicine

## Abstract

Despite the common usage of radiotherapy for the treatment of human non-small-cell lung cancer (NSCLC), cancer therapeutic efficacy and outcome with ionizing radiation remains a challenge. Here, we report the antitumor effects and mechanism of a novel benzothiazole derivative PB01 (4-methoxy-cyclohexane carboxylic acid [2-(3,5-dimethyl-isoxazole-4-yl) sulpanil-benzothiazole-6-yl]-amide) in radiation-resistant human NSCLC cells. PB01 treatment is cytotoxic because it induces reactive oxygen species, ER stress, Bax, cytochrome c expression, the ATR-p53-GADD45ɑ axis, and cleavage of caspase-3 and -9. Additionally, we found that radio-resistant A549 and H460 subclones, named A549R and H460R, respectively, show enhanced epithelial-to-mesenchymal transition (EMT), whereas PB01 treatment inhibits EMT and mediates cell death through ER stress and the ATR axis under radiation exposure in radio-resistant A549R and H460R cells. Together, these results suggest that PB01 treatment can overcome radio-resistance during radiotherapy of NSCLC.

## Introduction

Among cancer types, lung cancer has the highest cancer ranks, including the worldwide incidence and mortality statistics^1^. Despite many studies, lung cancer therapies often fail to cure the disease. Histologically, there are different types of lung cancer: small-cell lung cancer (SCLC) and non-small-cell lung cancer (NSCLC)^2^. Lung cancer can be treated by 3 major types (chemical, radiation, and surgery). Single therapy frequently has limitation for improving anti-cancer efficacy; therefore, combination therapy is the effective cancer therapeutic strategy to solve this problem^3^.

Radiation therapy is one of the powerful therapies for patients with NSCLC^4^. The diverse factors, including chemo-resistance/radio-resistance, local recurrence, and distant metastases cause to the failure of clinical radiation therapy^5^. Though IR causes cytotoxicity in cancer cells, increasing studies have suggested that IR mediates the malignancy and radio-resistance in various cancer cell types including lung, hepatocellular carcinomas, and gliomas^6–8^. Moreover, many studies using animal models indicated that radiation administered to primary tumors increases their spread and the formation of distant metastasis *in vivo*^9^. Recent reports explained molecular relationship between radio-resistance, chemo-resistance, and EMT phenotype in cancer^10–14^. During the process of EMT phenotype, carcinoma cells stop expressing proteins that promote cell–cell interaction such as E-cadherin and β-catenin, and they start over expressing of mesenchymal markers such as vimentin, fibronectin, and N-cadherin. This causes cytoskeletal remodeling as well as cancer cell migration and invasion^15^. The Snail and Slug, a zinc finger transcription factor and suppressor of E-cadherin, contribute to process of EMT^16–18^. Ectopic expression of Snail regulates to the inhibition and activation of epithelial and mesenchymal markers, and is associated with metastatic ability in carcinoma cell lines that include breast cancer cell lines^19^. Snail knockdown through RNA interference (RNAi) impairs the metastatic potential of fully metastatic tumor cell lines^20^. There is also evidence that Snail affects radiation resistance and paclitaxel treatment in ovarian cancer cells by antagonizing apoptosis signaling via p53 and inducing the acquisition of stem-like characteristics^21^. It can be hypothesized that Snail expression induces a critical changes in the genome-wide patterns of cancer cells and advances more aggressive phenotypes^22^. Overcoming drug resistance or radio-resistance factors, including EMT phenotype via radiation, hypoxia and nutrient deprivation, suggests potential cancer therapy strategies, and developing new compound and combination strategy to overcome chemo- or radio-resistance may lead to more effective therapeutic strategy.

Benzothiazole derivatives are found to have outstanding structures in anti-cancer drug and regulate various biological targets such as replication, mitosis, topoisomerase, cytochrome P450, cathepsin, and epidermal growth factor receptor (EGFR) as well as have important anti-tumor, anti-inflammation, and anti-diabetes functions^23^. Rational drug design is an important process to develop potential novel compound, and benzothiazole structure-based novel drug development is an effective approach. Some benzothiazole derivatives show anti-tumor effects in breast cancer cells (e.g. MCF7 and MDA-MB-468)^24^. It is reported that benzothiazole derivative-mediated cell death causes ROS production and cell cycle arrest during tumor therapy^25^. Interestingly, ROS production enhances the expression of anti-oxidant proteins, and ROS release inhibits cancer cell proliferation, increased ER stress, and cytotoxicity by arresting cells growth via DNA damage^26–28^. Ataxia telangiectasia mutated (ATM) and ataxia telangiectasia mutated and Rad3-related (ATR) demonstrated that key protein kinases can sense DNA damage and factors that cause DNA damage such as IR and UV radiation, to promote cell cycle arrest^29^. ATR regulates DNA damage and cell cycle checkpoints through p53 activation^30^. p53 activates GADD45ɑ in response to DNA damage signals, and GADD45ɑ, a known p53-regulated and DNA damage-inducible protein, has essential functions in cell cycle arrest and cell death after DNA damage^31–32^. Recently, it has been reported that ROS induces ER stress during cell survival and cell death, and it triggers DNA damage and oxidative stress^33^. ER stress can be caused by increased amounts of unfolded or misfolded proteins in the endoplasmic reticulum (ER), and severe or prolonged ER stress results in cell death^34^. Under ER stress, ER trans-membrane sensing proteins such as protein kinase RNA-like endoplasmic reticulum kinase (PERK) and inositol requiring enzyme 1 (IRE1ɑ) are activated by Bip/GRP78 and promotes the translation of the transcription factor CHOP as a pro-apoptotic marker during ER stress^35^. Furthermore, CHOP regulates the expression of GADD45 and induces DNA damage-mediated cell death^36^.

Strategies to reverse radiation resistance or to increase cellular sensitivity to radiation are crucial in the development of more effective cancer treatments. In the present study, we evaluated the role of PB01, a novel benzothiazole structure-based synthetic chemical, in modulating the response to radiation-induced EMT and ER stress in radio-resistant A549R and H460R cells. By targeting IR-activated EMT pathways, PB01 may be effective at countering IR in these cell types and improving their radio-therapeutic response.

## Materials and methods

### Reagents

PB01 was synthesized in the Department of Chemistry at the College of National Science, Kongju National University (Gongju, Korea) (Figure S1). PB01 was dissolved in distilled water (DW) as a 1 mM stock solution. Other compounds were obtained as follows: diphenyleneiodonium (Nox or ROS inhibitor, DPI, Sigma Aldrich, St Louis, MO, USA), Apocynin (a ROS inhibitor, Apo, Sigma Aldrich, St Louis, MO, USA), Z-VAD-FMK (caspase inhibitor, Sigma Aldrich, St Louis, MO, USA), and Thapsigargin (ER stress inducer, TG, Millipore, Bedford, MA, USA).

### Cell culture

A549 and H460 human NSCLC cell lines and MRC5 normal lung cell lines were obtained from the American Type Culture Collection (ATCC; Manassas, VA, USA). A549 and H460 cells were cultured in DMEM (Welgene, Daegu, South Korea) supplemented with 10% fetal bovine serum (FBS), 100 U/ml penicillin, and 100 mg/ml streptomycin (all from Welgene) at 37 °C under a humidified 95/5% (v/v) mixture of air and CO_2_ atmosphere incubator..

### Cell viability

Human NSCLC cells were seeded into a 96-well plate with DMEM medium and grown for 24 h for stabilization. Then, cells were treated and incubated with PB01 or DMSO for 24 h. Cell viability was investigated using a WST-1 assay (Roche, USA). 100 μL of WST-1 reagent were added, and the plates were incubated for 30 min at 37 °C CO_2_ incubator. Cell absorbance was measured at 450 nm using an enzyme-linked immunosorbent assay reader (SpectraMax190, Microplate Reader, Molecular Devices, CA, USA).

### LDH assay

Human NSCLC cells were seeded into a 96-well plate with DMEM medium and grown for 24 h for stabilization. Then, cells were treated and incubated with PB01 or DMSO for 24 h. To identify the LDH release in supernatants, LDH assay (Thermo Scientific Pierce) was performed on this experiment. 100 μL of the LDH reagent were added, and the plates were incubated for 30 min in dark room.. The LDH activity was determined by measuring the absorbance of the samples at 490 or 492 nm using the ELISA reader (SpectraMax190, Microplate Reader, Molecular Devices, CA, USA).

### Caspase-3 and -9 activity assays

Caspase-3 and -9 activity assays (the Biovision colorimetric caspase-3 and -9 assay kit) were performed according to the manufacturer’s instructions. Human NSCLC cells (A549 and NCI-H460) were seeded into a 6-well plate with DMEM medium and grown for 24 h for stabilization. Then, cells were treated and incubated with PB01 or DMSO in a dose-dependent manner (0, 50, 100, and 200 nM; 24 h). These cells added with cell lysis buffer (50 μL) and incubated for 10 min on ice. Samples were centrifuged at 10,000 g for 1 min and quantified protein concentration. Twenty μg of total cellular protein was prepared for this experiment and added and mixed with 2X reaction buffer (50 μL) and 4 mM DEVD-*p*NA substrate (5 μL) or 4 mM LEHD-*p*NA substrate (5 μL), respectively. After incubation for 1 h at 37 °C, caspase-3 and -9 activities were analyzed at 405 nm using spectrophotometer (Molecular devices).

### Colony formation assay

Cells (A549, A549R, NCI-H460 and NCI-H460R; 1 × 10^3^) were plated and seeded onto 60 mm dishes with growth medium and grown for 24 h at 37 °C CO_2_ incubator. Cells incubated for 10-days, and when cells perform the colony formation, the colonies were stained with 0.5% crystal violet. To calculate the survival fraction, the number of colonies form was divided by the number of seeded cells form of the control plate.

### Fluorescence activated cell sorting (FACS) analysis

Human NSCLC cells were seeded onto 100 mm dishes with DMEM medium and grown for 24 h for stabilization. Then, cells were treated and incubated with PB01 (100 nM) or DMSO for 24 h. For cell cycle analysis, NSCLC cells were fixed with cold 80% ethanol and stained with a solution containing PI and RNase A (Sigma). The ROS generation during apoptosis was examined by monitoring the cells after staining with the cell-permeant 2′7′-dichlorodihydrofluorescein diacetate (CM-H_2_DCFDA, Invitrogen). For annexin V analysis, cells were fixed and stained annexin V/PI dye for 30 min using annexin V kit (Biovision, Palo Alto, CA, USA). Data were analyzed with a FACS Calibur flow cytometer (Becton Dickinson, San Jose, CA, USA).

### Irradiation

Cells (A549, A549R, NCI-H460 and NCI-H460R; 1 × 10^3^) were seeded and plated onto 60 mm dishes with growth medium and incubated at 37 °C CO_2_ incubator. Cells incubated for 24 h, and ionizing radiation (IR) exposure were carried out using ^137^Cs source irradiation (Atomic Energy of Canada, Ltd., Mississauga, ON, Canada). To establish radio resistant cells, cells were subjected to 2 Gy dose for 90 days. After completing of IR exposure, cells were grown with growth medium containing 10% FBS.

### Development of radio-resistant A549 and H460 cell lines

A549 and H460 cells were plated into 60-mm dishes. After 24 h, to generate parental cell lines, these cells were exposed at dose of 2 Gy daily for 12 weeks. For development radio-resistant A549R and H460R cell lines, selected radio-resistant cells were verified and established by comparing with parental cells.

### Scratch wound migration assay

A549 and A549R cells were plated into 6-well plates at a density to reach 100% confluence. Scratch wound migration assays were conducted following manufacturer’s protocol (Life technologies). Confluent monolayers of these cells were scratched with a sterile p200 pipet tip, washed with PBS and added to growth medium. These cells were subjected to treatment with PB01 and phase contrast images were monitored during migration after 24 h of treatment. The area of migrating cells was randomly monitored and photographed using optical microscopy (Zeiss).

### Invasion assay

A549 and A549R cells were incubated for 24 h in serum-free medium and treated with PB01 for 24 h. Cells (2 × 10^5^ cell/ml) were re-plated onto 8 μm-pore Transwell filters (Corning) in a 12-well plate following the manufacturer’s protocol (Corning). Invaded cells were stained with crystal violet, and the number of invaded cells were analyzed in five selected areas and complied as the mean of five repeats per filter. The results represent the average and SD of three independent experiments.

### Transfection

Human NSCLC cells (5 × 10^5^) in a 6-well plate were transfected with either ATR (Santacruz), p53 (Santacruz), PERK (Santacruz), or IRE1ɑ (Santacruz) double-stranded siRNAs (30 nmol/ml), for 24 h using Lipofectamine 2000 reagent (Invitrogen) according to the manufacturer’s instructions.

### Isolation of total RNA and protein

Total RNA from human NSCLC cells in a 100 mm cell culture dish was prepared using Trizol reagent according to the manufacturer’s instructions (Invitrogen). Protein cell lysates were collected in radioimmunoprecipitation assay (RIPA) lysis buffer (Bio-rad). The supernatant was analyzed for protein content using the BCA method (Thermo Scientific).

### Real-time PCR and Western blot analyses

Reactions were performed in triplicate for each sample using an ABI Power SYBR green PCR Master Mix (Applied Biosystems) with N-cadherin-specific primers [5′-GGCATACACCATG CCATCTT-3′ (sense) and 5′-GTGCATGAAGGACAGCCTCT-3′ (antisense)], Vimentin-specific primers [5′-GAGAACTTTGCCGTTGAAGC-3′ (sense) and 5′-GCTTCCTGTAGGTGGCAATC-3′ (antisense)], E-cadherin-specific primers [5′-GAACGCATTGCCACATACAC-3′ (sense) and 5′-GAATTCGGGCTTGTTGTCAT-3′ (antisense)], Slug-specific primers [(5′-CATGCCTGTCATACCACAAC-3′ (sense) and 5′-GGTGTCAGATGGAGGAGGG-3′ (antisense)], and Snail-specific primers [(5′-GAGGCGGTGGCAGACTAG-3′ (sense) and 5′-GACACATCGGTCAGACCAG-3′ (antisense)] on a Roche LightCycler 96 System (Roche) . RNA quantities were normalized with β-actin primers [5′-AAGGCCAAC CGCGAGAAGAT-3′ (sense) and 5′-TGATGACCTGGCCGTCAGG-3′ (antisense)], and gene expression was quantified according to the 2^−ΔCt^ method. To perform the Western blot analysis, human NSCLC cells were solubilized in RIPA lysis buffer (Bio-rad). Equal amount of protein (20 μg) were size-fractionated by 8 ~ 15% SDS-PAGE and then transferred PVDF membrane (Amersham, Germany). Transferred membranes were blocked by incubation for 30 min with 1X PBS-T buffer (PBS with 0.05% Tween-20) containing 5% skim-milk and washed with 1X PBS-T buffer. Then, blocked membranes incubated overnight at 4 °C with a 1:1000 dilution of primary antibodies in 1X PBS-T buffer. The primary antibodies used included β-actin (Santa Cruz, 1:1000, sc-47778), p53 (Santa Cruz, 1:1000, sc-126), p-p53 (Ser315) (Santa Cruz, 1:1000, sc-377567), eIF2ɑ (Santa Cruz, 1:1000, sc-133132), JNK (Santa Cruz, 1:1000, sc-7345), GADD45ɑ (Santa Cruz, 1:1000, sc-6850), BAX (Santa Cruz, 1:1000, sc-7480), VDAC (Santa Cruz, 1:1000, sc-390996), and GRP78 (Santa Cruz, 1:1000, sc-166490); IRE1ɑ (Abcam, 1:1000, ab37073) and p-IRE1ɑ (Abcam, 1:1000, ab48187); and cleaved caspase-3 (Cell Signaling, 1:1000, #9664), caspase-9 (Cell Signaling, 1:1000, #20,750), ATR (Cell Signaling, 1:1000, #2790), p-ATR (Ser428) (Cell Signaling, 1:1000, #2853), p-PERK(Thr980) (Cell Signaling, 1:1000, #3179), PERK (Cell Signaling, 1:1000, #5683), ATF4 (Cell Signaling, 1:1000, #11,815), CHOP (Cell Signaling, 1:1000, #2895), ɣH2AX (Cell Signaling, 1:1000, #9718), p-eIF2ɑ (Ser51) (Cell Signaling, 1:1000, #3398), p-JNK (Thr183/Tyr185) (Cell Signaling, 1:1000, #9255), cytochrome c (Cell Signaling, 1:1000, #11,940), p-ERK (Thr202/Tyr204) (Cell Signaling, 1:1000, #4370), p-Akt (Ser473) (Cell Signaling, 1:1000, #4060), p-p38 (Thr180/Tyr182) (Cell Signaling, 1:1000, #4511), E-cadherin (Cell Signaling, 1:1000, #14,472), N-cadherin (Cell Signaling, 1:1000, #13,116), Slug (Cell Signaling, 1:1000, #9585), Snail (Cell Signaling, 1:1000, #3879), Cyclin B1 (CellSignaling, 1:1000, #4138) and vimentin (CellSignaling, 1:1000, #5741). After this incubation, the membranes were washed 3 times with PBS-T buffer and incubated for 40 min at room temperature with a 1:4000 dilution of HRP-conjugated secondary antibodies in PBS-T buffer. The secondary antibodies including anti-mouse anti rabbit IgG HRP-linked antibody (Santa Cruz, sc-2357) and m-IgGK BP-HRP-linked antibody (Santa Cruz, sc-516102) were purchased from Santa Cruz Biotechnology and used to these experiment. The blots were visualized by ECL Prime Western Blotting Detection Reagents (Amersham, UK).

### Isolation of mitochondria and cytosols

Human NSCLC cells were seeded and grown into a 100 mm plate with DMEM medium and Cytoplasmic and mitochondrial fractions were isolated using a mitochondria isolation kit (Pierce, Rockford, IL, USA) according to the manufacturer’s instructions.

### Immunofluorescence staining

A549 and A549R cells (5 × 10^5^) were plated on glass coverslips into 6-well plates for 24 h in growth medium and then treated with PB01 for 24 h following the manufacturer’s protocol (Life technologies). A549 and A549R cells were fixed with 4% formaldehyde, permeabilized with PBS supplemented with 0.5% Triton X-100 and blocked with blocking solution. Cells were incubated and stained with primary antibodies against N-cadherin and E-cadherin overnight at 4 °C and were stained with the secondary antibodies against Alexa-488 (Thermo Fisher Scientific) and Alexa-555 (Thermo Fisher Scientific) for 1 h at 37 °C. Nucleus were stained with DAPI (4′,6-diamidino-2-phenylindole, Sigma Aldrich). The image of stained cells was monitored and analyzed using confocal laser fluorescence microscopy (Zeiss, LSM 710).

### Statistical analyses

Data are expressed as the mean ± the standard error (SE). Statistical analyses of the experimental data were performed using a two-sided Student's *t*-test. P values < 0.05 were deemed statistically significant.

## Results

### PB01 inhibits the proliferation of NSCLC cells

**Figure 1:**
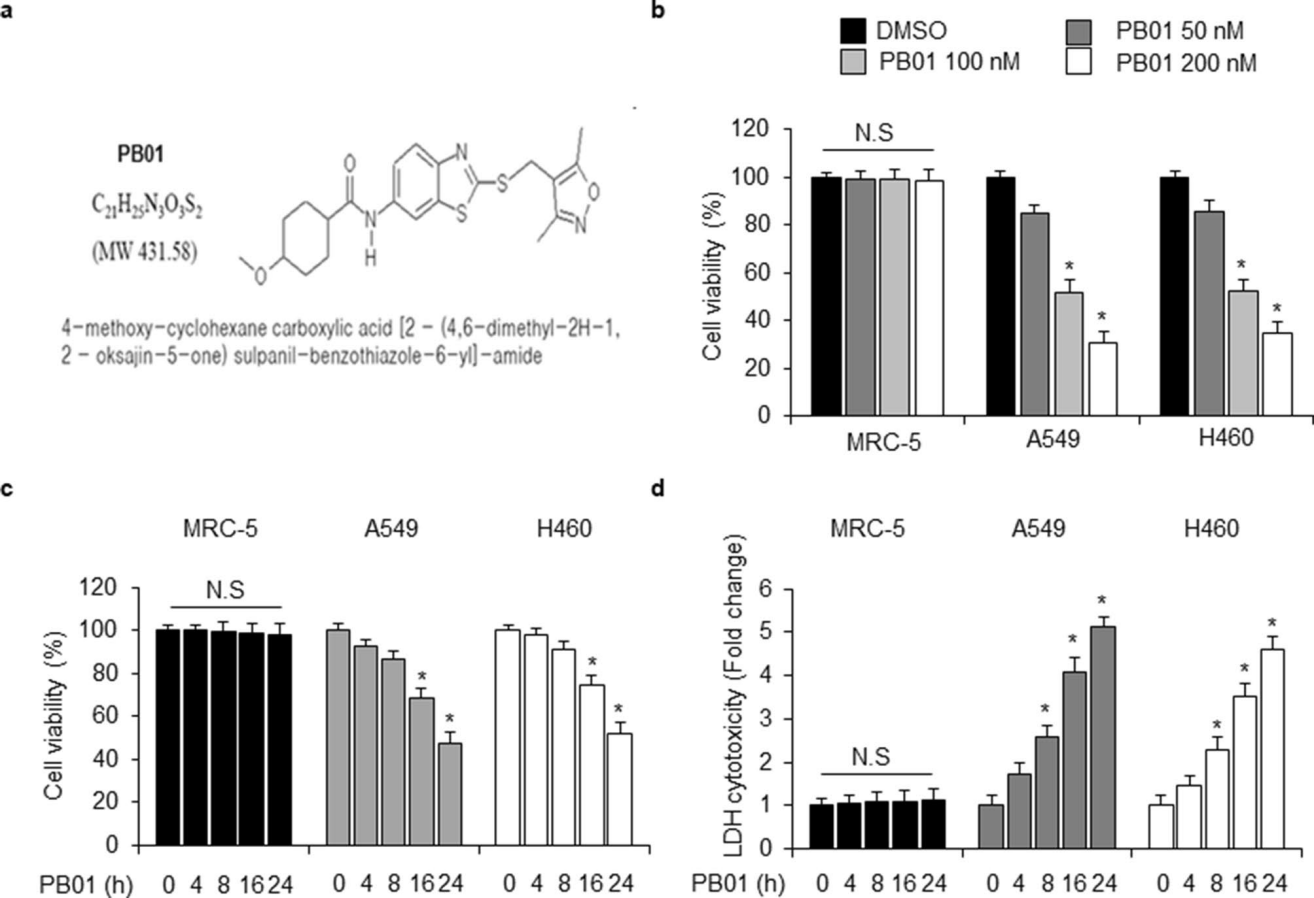
PB01 inhibits the proliferation of NSCLC cells. (**a**) The chemical structure of PB01. (**b**) MRC5 normal lung cells and human NSCLC A549 and H460 cells were incubated with various concentrations of PB01 for 24 h. Cell viability was determined using a WST-1 assay; *p < 0.05. (**c**,**d**) MRC5 normal lung cells and human NSCLC A549 and H460 cells were incubated with PB01 (100 nM) for various times; *p < 0.05. Cell viability was determined using a WST-1 assay, and LDH cytotoxicity was determined using an LDH assay.

In a search to identify novel anti-cancer agents, we synthesized a 4-methoxy-cyclohexane carboxylic acid (4-methoxy-cyclohexane carboxylic acid [2-(3,5-dimethyl-isoxazole-4-yl) sulpanil-benzothiazole-6-yl]-amide) (PB01) (Fig. 1a). The ability of PB01 to block cell growth was investigated using a WST-1 assay. As shown in Fig. 1b,c, PB01 inhibited the growth of lung cancer cell lines in a concentration and time-dependent manner (0, 50, 100, and 200 nM for 24 h; 0, 4, 8, 16, and 24 h at 100 nM) when compared with a normal lung cell line (MRC5). Figure 1d shows that PB01 increased LDH cytotoxicity in a time-dependent manner in A549 and H460 cells when compared to MRC5 cells. These results indicate that PB01 inhibits the proliferation of NSCLC cells. This activity makes PB01 a promising anti-lung cancer compound.

### PB01 induces apoptotic cell death in NSCLC cells

**Figure 2:**
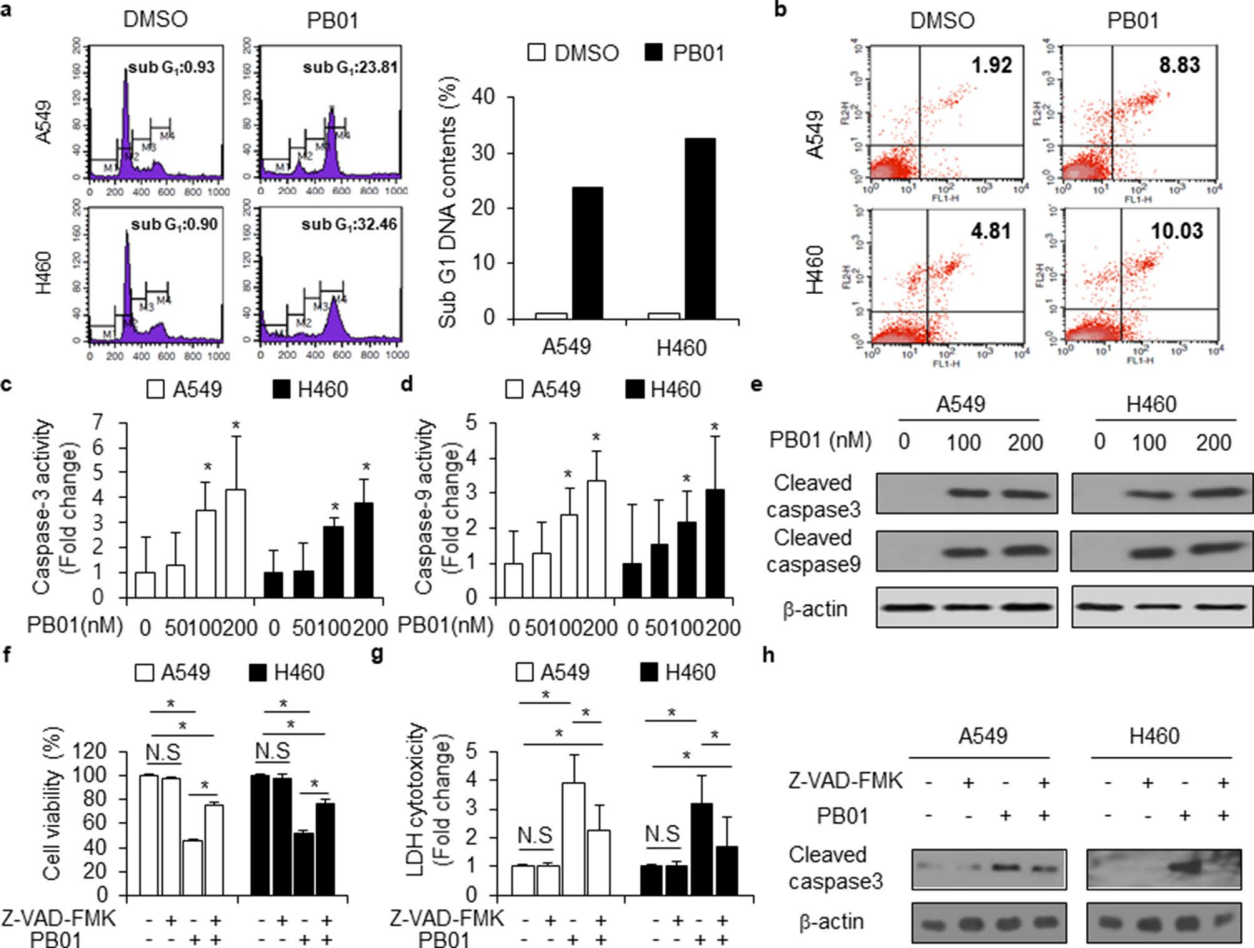
PB01 regulates apoptotic cell death in NSCLC cells. (**a**) Cell cycle arrest by PB01 in A549 and H460 NSCLC cells, was measured using FACS, and the percentage represents the cell population in sub-G-1 (**b**,**c**) Caspase-3 and -9 activities in PB01 (0, 50, 100, and 200 nM, 24 h)-treated A549 and H460 cells; *p < 0.05. (**d**) Western blot of cleaved caspase-3 and caspase-9 analyzed for the indicated doses in PB01-treated A549 and H460 cells. (**e**–**g**) The effect of a pan-caspase inhibitor, Z-VAD-FMK(50 mM, 24 h), on PB01-induced apoptotic cell death. A549 and H460 cells were pretreated with Z-VAD-FMK for 4 h and subsequently treated with PB01 (100 nM, 24 h); *p < 0.05. Caspase-3 activity was performed using caspase-3 activity assay, cell viability was determined using a WST-1 assay, and the LDH cytotoxicity was determined using an LDH assay. (**h**) Western blot analysis identifying the activation of apoptosis markers such as cleaved caspase-3 analyzed in PB01 and Z-VAD-FMK-co-treated A549 and H460 cells. β-actin was used as the protein loading control.

Previous studies have shown that benzothiazole derivatives frequently trigger apoptosis, making them potent antitumor agents^37^. In a cell cycle arrest analysis, A549 and H460 cells were treated with PB01 (100 nM) for 24 h and harvested. The percentage of A549 and H460 cells in sub G-1 phase was significantly increased in cells treated with PB01 (Fig. 2a). To identify the effect of PB01 on apoptosis in A549 and H460 cells, we performed a caspase-3 and -9 activity assays. As shown in Fig. 2b,c, PB01 enhanced the active caspase-3 and -9 value of these cells in a dose-dependent manner (0, 50, 100, and 200 nM). We also investigated whether PB01 induced apoptosis in a caspase-dependent manner using Western blot analysis. PB01 significantly increased the cleavage of caspase-3 and -9 at the indicated dose points (Fig. 2d). To probe whether PB01-treated apoptosis is regulated by a pan-caspase inhibitor (Z-VAD-FMK), we treated NSCLC cells with Z-VAD-FMK (50 µM, 24 h) and PB01 (100 nM, 24 h). Our results indicate that Z-VAD-FMK inhibits the decrease of cell viability and the increase of LDH cytotoxicity and caspase-3 activity caused by PB01 in NSCLC cells (Fig. 2e–g). Immunoblot analysis shows that PB01 + Z-VAD-FMK co-treatment inhibits the cleavage of caspase-3 when compared with PB01 treatment alone (Fig. 2h).

### PB01 induces apoptotic cell death via the ATR axis in NSCLC cells

**Figure 3:**
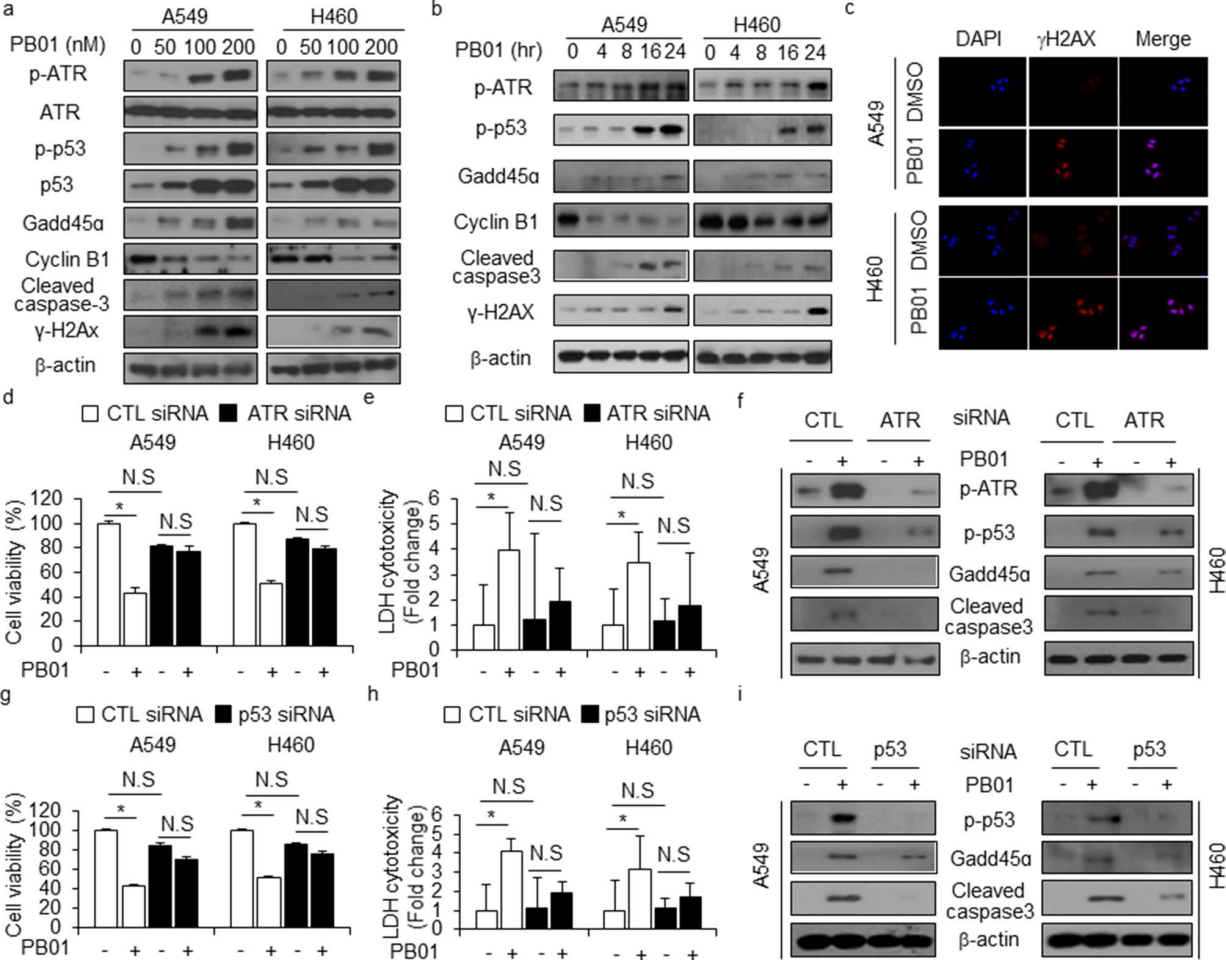
PB01 regulates apoptotic cell death via ATR-p53-GADD45ɑ in NSCLC cells. (**a**–**c**) Western blot of p-ATR, ATR, p-p53, p53, Cyclin B1, GADD45ɑ, cleaved caspase-3, and Immunofluorescence assay for ɣH2AX analyzed for the indicated doses and times in PB01-treated A549 and H460 cells. (**d**–**i**) After A549 and H460 cells were transfected with ATR and p53 siRNA, cell viability, LDH, and Western blot assays were performed after PB01 (100 nM, 24 h) treatment; *p < 0.05. β-actin was used as the protein loading control.

Various stresses, including DNA damage, hypoxia, and ROS, induce apoptotic cell death via activation of protein kinases such as ATR, ATM, CHK1, and CHK2, and they cause cell cycle arrest, apoptosis, and DNA damage through phosphorylation of p53^38–39^. Based on our results, which indicate that PB01 dramatically induces apoptosis, cell death, and cell cycle arrest in NSCLC cells, we investigated whether PB01 modulates DNA damage and ROS generation. As expected, PB01 significantly enhanced p-ATR, p-p53, p53, GADD45ɑ, cleaved caspase-3, and ɣH2AX expression in a dose- and time-dependent manner (Fig. 3a,b). Immunofluorescence assays showed that PB01 increases ɣH2AX expression, which indicate DNA damage, in A549 and H460 cells (Fig. 3c). To further investigate whether PB01 modulates apoptotic cell death via the ATR-p53-GADD45ɑ axis in NSCLC cells, we used siRNAs against, namely ATR and p53. NSCLC cells were transfected with ATR and p53 siRNAs and treated with PB01. The viability increased and the release of LDH decreased in cells treated with ATR and p53 siRNA and PB01 when compared to control cells (Fig. 3d,e,g,h). Western blot analysis showed that PB01 reduced ATR, p-p53, GADD45ɑ, and cleaved caspase-3 expression in ATR knock-down cells when compared in control cells (Fig. 3e). p53 knock-down led to reduced p-p53, GADD45ɑ, and cleaved caspase-3 expression in A549 and H460 cells when compared to control cells (Fig. 3i). Therefore, our findings indicate that ATR-p53-GADD45ɑ signaling plays a powerful role in the apoptotic cell death of NSCLC cells treated with PB01.

### PB01 induces ER stress-mediated cell death in NSCLC cells

**Figure 4:**
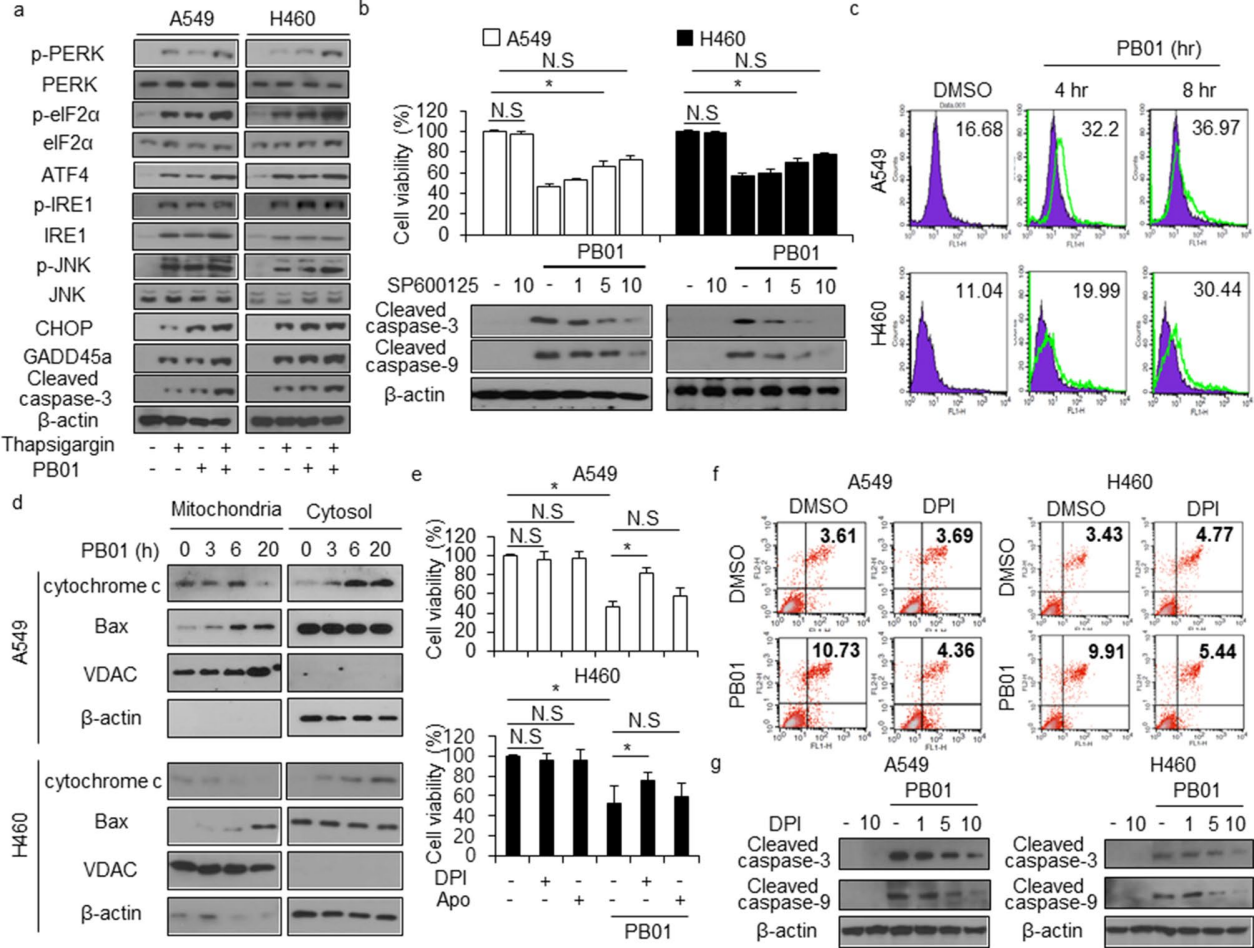
DPI inhibits multiple PB01-mediated cell death pathways in NSCLC cells. (**a**) Western blot of p-PERK, PERK, p-eIF2α, eIF2α, ATF4, p-IRE1α, IRE1α, p-JNK, JNK, CHOP, GADD45α, and cleaved caspase-3 levels from thapsigargin (3 μM, 24 h) and PB01 (100 nM, 24 h)-treated A549 and H460 cells. β-actin was used as the protein loading control. (**b**) A549 and H460 cells were pretreated with SP600125 (1, 5, and 10 μM, for 4 h) and subsequently treated with PB01 (100 nM, 24 h). Cell viability was determined using a WST-1 assay; *p < 0.05. Western blot analysis was conducted on total lysates to identify the inhibition of apoptosis markers such as cleaved caspase-3 and -9. β-actin was used as the protein loading control. (**c**) The FACS data indicates the change in fluorescence intensity of DCFDA dye in PB01 (100 nM, 4 and 8 h)-treated A549 and H460 cells. (**d**) A Western blot of cytochrome c and Bax from the cytosolic and mitochondrial fractions was performed in PB01 (100 nM, 0, 3, 6, and 20 h)-treated A549 and H460 cells. β-actin and VDAC were used as protein loading controls for the cytosolic and mitochondrial fractions, respectively. (**e**) A549 and H460 cells were pretreated with DPI (1 μM) and NAC (100 μM) for 4 h and subsequently treated with PB01 (100 nM, 24 h). Cell viability was determined using a WST-1 assay; *p < 0.05. (**f**) A549 and H460 cells were pretreated with DPI (1 μM) for 4 h and subsequently treated with PB01 (100 nM, 24 h). Apoptotic cell death was determined using a Annexin V FACS assay. (**g**) Western blot showing cleaved caspase-3 and -9 levels was performed using these samples. β-actin was used as the protein loading control.

ER stress induces p53-dependent cell death in DNA damage-mediated cancer cells^40^. To investigate whether PB01 modulates ER stress and DNA damage pathways in NSCLC cells, we measured the levels of ER stress proteins, such as p-PERK, PERK, p-eIF2α, eIF2α, ATF4, p-IRE1ɑ, IRE1ɑ, p-JNK, JNK, cleaved caspase-3, CHOP, and DNA damage protein GADD45ɑ using Western blot analysis in thapsigargin (TG)-treated NSCLC cells. PB01, in combination with TG, induces a synergistic increase in these ER stress proteins (Fig. 4a).

To identify whether PB01 effect the mitogen-activated protein kinase (MAPK) pathway, we monitored the expression levels of p-JNK, p-ERK, p-p38, and p-Akt in a time-dependent manner (Figure S2a). The results showed that PB01 induces time-dependent phosphorylation of JNK but not p-ERK, p-p38, and p-Akt. To identify whether PB01 regulates the MAPK pathway in NSCLC cells, we examined the effects of SP600125 (a JNK inhibitor), U0126 (an ERK inhibitor), and SB203580 (a p38 inhibitor) on cell viability and cell cycle arrest. We also conducted a Western blot analysis to measure caspase-3 cleavage. U0126 and SB203580 did not affect cell viability, cell cycle arrest, and cleaved caspase-3 levels in PB01-treated NSCLC cells; SP600125 decreased cell viability, cell cycle arrest, and cleaved caspase-3 levels in PB01-treated NSCLC cells (Figure S2b–d). Moreover, PB01 treatment inhibited decrease of cell viability and cleaved caspase-3 expression in SP600125-induced cells in a dose-dependent manner (Fig. 4b). These findings suggest that PB01 causes JNK phosphorylation-dependent cell death in NSCLC cells, and PB01, in combination with SP600125, inhibits cell death.

ROS release is a critical mediator of apoptosis^41^. To determine whether PB01 triggers ROS generation, ROS levels were measured using DCFDA, a fluorescent dye. PB01 significantly increased ROS levels at each in indicated time condition when compared to the control (Fig. 4c). Recent reports have indicated that ROS mediates various apoptotic cell death pathways such as the mitochondrial pro-apoptotic pathway, and it is related to Bax and cytochrome c release^42^. Mitochondrial and cytosolic fractions quantified by VDAC and β-actin in PB01-treated cells in a time-dependent manner revealed that the cytosolic cytochrome c level was increased in accordance with the decrease in the mitochondrial cytochrome c level in A549 and H460 cells after PB01 treatment (Fig. 4d). At the same time, PB01 causes a time-dependent increase in Bax expression in the mitochondrial fraction, but it causes a time-dependent decrease in Bax in the cytosol fraction (Fig. 4d). These results indicate that PB01 cause cytosolic cytochrome c and mitochondrial Bax to be released in A549 and H460 cells. Next, we performed pharmacological experiments with DPI (an NAD(P)H Oxidase (NOX) inhibitor) and Apo (a p47phox inhibitor), and DPI inhibits decrease of cell viability in PB01-treated NSCLC cells, but not Apo (Fig. 4e). Annexin V assay indicated that DPI inhibits PB01-mediated apoptosis in A549 and H460 cells (Fig. 4f). Western blot analysis showed that DPI treatment significantly decreased PB01-induced cell death by decreasing cleaved caspase-3 and 9 expression (Fig. 4g). These results suggest that DPI attenuates PB01-induced ROS production and cell death via NOX inhibition.

### A549R and H460R cells mediate change of EMT regulatary markers

**Figure 5:**
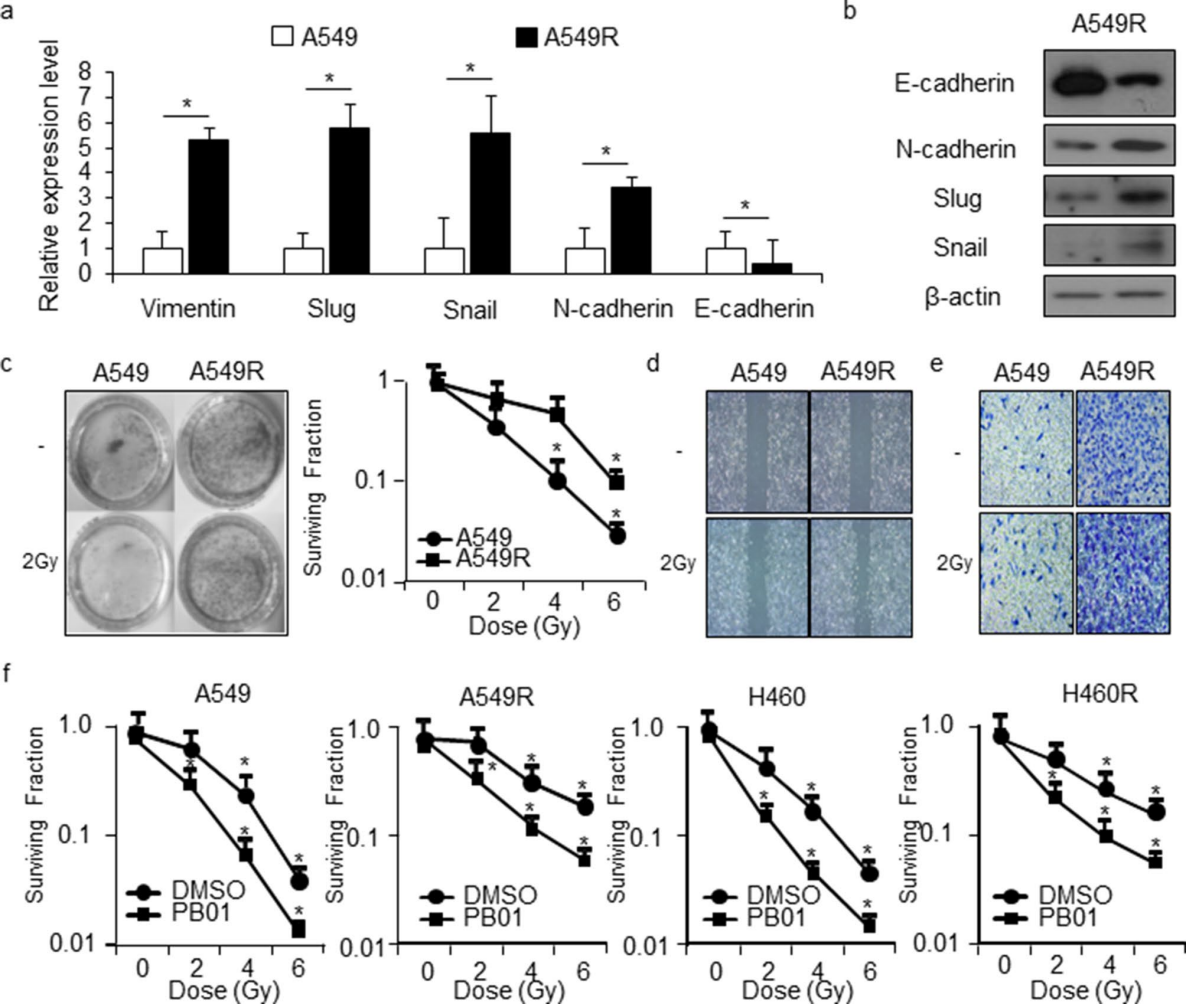
Established radio-resistant A549R and H460R cells acquire an EMT phenotype. (**a**,**b**) Real-time RT-PCR was used to measure the expression of E-cadherin, N-cadherin, vimentin, Slug, and Snail in A549 and A549R cells, and Western blot analysis was used to measure the expression of E-cadherin, N-cadherin, Slug, and Snail in A549 and A549R cells; *p < 0.05. β-actin was used as the RNA and protein loading control. (**c**) A clonogenic cell survival assay was performed using various doses (2, 4, or 6 Gy) of radiation, and the survival fraction was calculated using the surviving fraction formula in A549 and A549R cells; *p < 0.05. (**d**) Radiation (2 Gy) was exposed in A549 and A549R cells. The migratory ability of A549 and A549R cells was observed using a wound-healing assay where cells were scratched with a pipette tip. (**e**) The invasiveness of A549 and A549R cells was observed by a Matrigel invasion assay. Results represent the means ± SEM of 3 independent experiments; *p < 0.05. (**f**) A clonogenic cell survival assay was performed after PB01 (100 nM, 24 h) in combination with various radiation doses (2, 4, or 6 Gy), and the survival fraction was calculated using the surviving fraction formula in A549, H460, A549R, and H460R cells; *p < 0.05.

A549 human NSCLC cells were used to generate irradiation-resistant cells. When the cells reached approximately 60% confluency, they were exposed to radiation. A549 and H460 cells were irradiated 20 times (once a day) with the dose of 2 Gy and when cells reached 90% confluency, they were subcultured. Untreated parental A549 cells were cultured under the same conditions without irradiation. A549 and H460 cells that survived multiple fractions of radiation treatment (in total 100 Gy) were named A549R and H460R, respectively. EMT causes resistance to various antitumor therapies, and it also was induces radiation resistance^43^. To determine if the acquisition of radiation resistance induces specific molecular changes consistent with EMT, we examined the expression of epithelial and mesenchymal phenotype markers. As shown in Fig. 5a,b, the mRNA and protein expression of E-cadherin was significantly lower in the A549R cells than in the parental A549 cells. In contrast, the expression levels of N-cadherin, vimentin, Slug, and Snail were increased. A clonogenic survival assay was employed to compare the radio-sensitivity of A549R cells with A549 parental cells. A549R cells demonstrated significantly higher levels of clonal survival after radiation (2, 4, and 6 Gy) treatment when compared to the parental A549 cells (Fig. 5c). Next, we investigated the effects of radiation (2 Gy) treatment on the migratory ability and invasive potential of the A549R and parental A549 cells. As shown in Fig. 5d,e, the migration ability and wound closure of A549R cells were significantly increased when compared with parental A549 cells, and radiation (2 Gy) exposure in A549R cells also increased when compared with parental A549 cells. These results demonstrate that acquired radiation resistance causes A549R cells to undergo EMT and enhances cancer migration and invasion ability.

### PB01 suppresses the EMT phenotype in NSCLC and radio-resistant NSCLC cells

**Figure 6:**
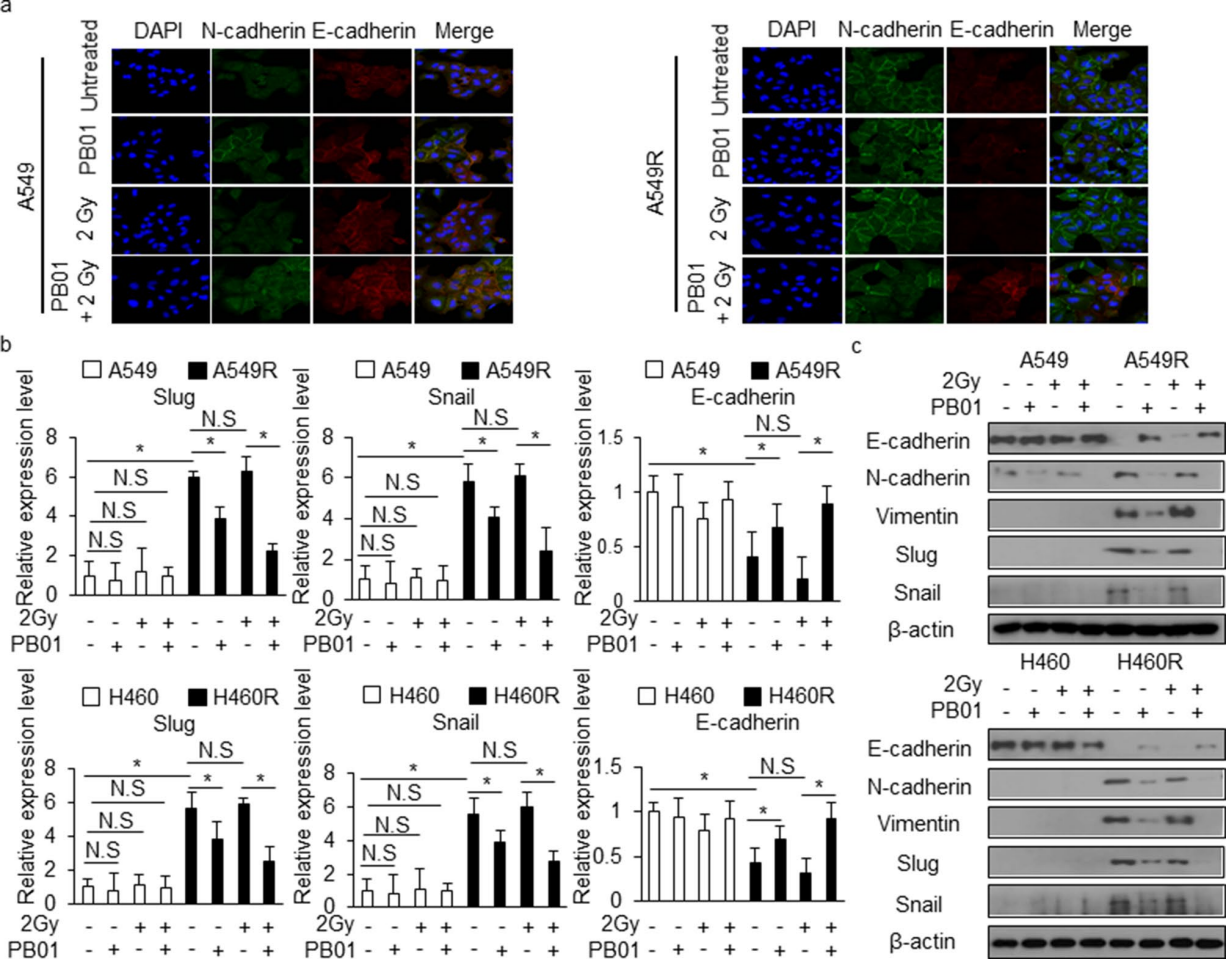
PB01 blocks the EMT phenotype in radiation-resistant NSCLC cells exposed to radiation. (**a**) A549 and A549R cells were pretreated with PB01 (100 nM) for 2 h and then stimulated and incubated for 24 h after 2 Gy exposure. The cells were then subjected to immunofluorescence staining for E-cadherin and N-cadherin. DAPI was used to stain nuclei. (**b**,**c**) A549, H460, A549R and H460R cells were pretreated with PB01 (100 nM) for 2 h and then stimulated and incubated for 24 h after 2 Gy exposure. Then, the RNA and protein from the samples were subjected to real-time RT-PCR and Western blot analysis to measure for E-cadherin, N-cadherin, vimentin, Slug and Snail expression; *p < 0.05. β-actin was used as the RNA and protein loading control.

To investigate radio-sensitivity caused by PB01 in NSCLC and radio-resistant NSCLC cells, we performed colony formation analyses. PB01 decreased the survival rates of A549, A549R, H460, and H460R cells exposed to a gradient of radiation (2, 4, and 6 Gy) in a dose-dependent manner when compared to control cells. However, A549 and H460 cells were more susceptible to PB01 than A549R and H460R cells (Fig. 5f). In addition, we calculated the combination index (CI) for PB01 and radiation combination with CacuSyn software and these results indicated synergistic action of the combination PB01 and radiation in A549, A549R, H460 and H460R cells (Figure S3). These findings indicate that PB01 increases radio-sensitivity in NSCLC and radio-resistant NSCLC cells. To further probe the radio-sensitivity effect of PB01 in NSCLC and radio-resistant NSCLC cells, a single treatment of PB01 and radiation (2 Gy) was performed separately or in combination. To examine whether PB01 affects EMT markers in radiation-treated NSCLC and radio-resistant NSCLC cells, we treated A549 and A549R cells with a combination of PB01 and 2 Gy radiation. Confocal imagery coupled with immunofluorescence staining showed a significant increase in E-cadherin and a reduction of N-cadherin in PB01/2 Gy-treated A549R cells when compared to untreated A549R cells (Fig. 6a). Real-time RT PCR and Western blot analysis indicated that PB01 and PB01/2 Gy treatments inhibited N-cadherin, vimentin, Slug, and Snail expression and increased E-cadherin in A549R and H460R cells, whereas they remained largely unchanged in A549 and H460 cells (Fig. 6b,c and Figure S4). Furthermore, the decreased in Slug and Snail expression and the increase in E-cadherin in A549R and H460R cells were more prominent when treated with a combination of PB01 and 2 Gy (Fig. 6b,c). These results indicate that PB01 can overcome radio-resistance through EMT inhibition in A549R and H460R cells.

### A combination of PB01 and radiation induce ER stress and apoptotic cell death in radio-resistant NSCLC cells

**Figure 7:**
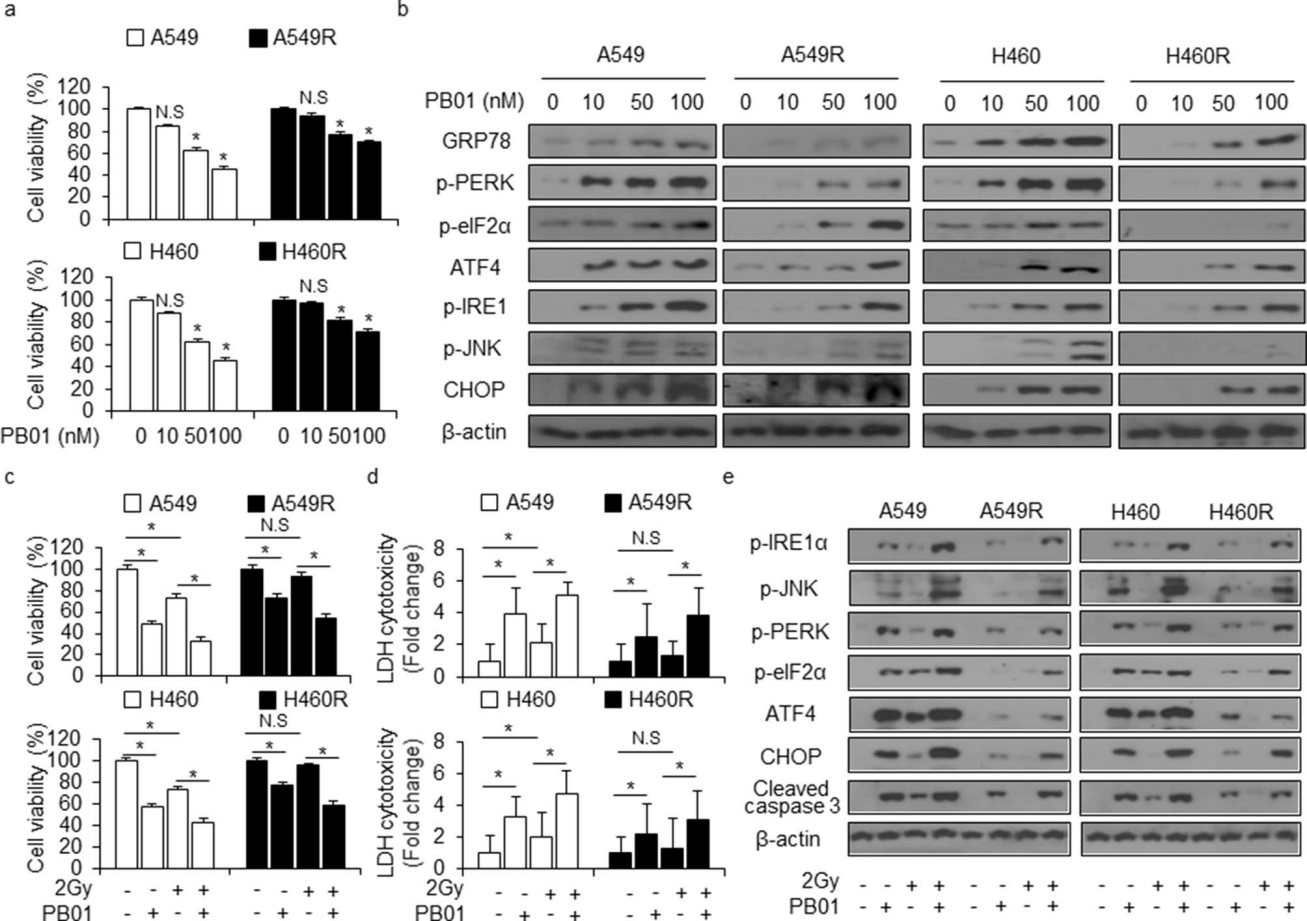
PB01, in combination with radiation, induces ER stress-mediated cell death in radiation resistant NSCLC cells. (**a**) A549, A549R, H460 and H460R cells were incubated with various concentrations (0, 10, 50, and 100 nM) of PB01 for 24 h. Cell viability was determined using a WST-1 assay; *p < 0.05. (**b**) Western blotting analysis of the ER stress markers GRP78, p-PERK, p-eIF2ɑ, ATF4, p-IRE1ɑ, p-JNK and CHOP was conducted for the indicated doses (0, 10, 50, and 100 nM) in PB01-treated A549, A549R, H460 and H460R cells. β-actin was used as the protein loading control. (**c**–**e**) A549, A549R, H460 and H460R cells were pretreated with PB01 (100 nM) for 2 h and then stimulated and incubated for 24 h after 2 Gy exposure. Cell viability was determined using a WST-1 assay, and the LDH cytotoxicity was determined using an LDH assay. Western blotting analysis of p-PERK, p-eIF2ɑ, ATF4, p-IRE1ɑ, p-JNK, CHOP and cleaved caspase-3 was performed using these samples in A549, A549R, H460, and H460R cells. β-actin was used as the protein loading control; *p < 0.05.

Several reports suggest that ER stress blocks chemo-resistance and causes cell death via EMT inhibition^44^. Our above indicate that PB01 causes ER stress-induced cell death in NSCLC cells. Therefore, we hypothesized that PB01-induced ER stress may mediate cell death by inhibiting EMT in radio-resistant NSCLC cells. We found that PB01 causes a dose-dependent reduction in the viability of NSCLC cells and radio-resistant NSCLC cells but increases viability in A549R and H460R cells when compared to A549 and H460 (Fig. 7a). Additionally, PB01 mediates the expression of ER stress markers such as GRP78, p-PERK, p-eIF2ɑ , ATF4, p-IRE1ɑ, p-JNK, and CHOP in a dose-dependent manner with A549 and H460 cells showing higher levels of ER stress markers than A549R and H460R cells (Fig. 7b). Treating A549, H460, A549R, and H460R cells with PB01, alone, decreased cell viability and increased LDH cytotoxicity when compared to cells treated with 2 Gy alone. Treating A549, H460, A549R, and H460R cells with a combination of PB01 and 2 Gy caused a greater decrease in cell viability and a greater increase in LDH release in A549R and H460R cells than cells treated with PB01 or 2 Gy, alone (Fig. 7c,d). Moreover, the PB01/2 Gy combination treatment had a greater effect on cell death in A549 and H460 cells, but it also overcame radio-resistance in A549R and H460R cells and induced cell death (Fig. 7c,d). Western blot analysis suggests that PB01 causes ER stress and apoptotic cell death by increasing the expression of p-PERK, p-eIF2ɑ, ATF4, p-IRE1ɑ, p-JNK, CHOP, and cleaved caspase-3 in A549 and H460 cells when compared to cells treated with 2 Gy, alone, but the combination treatment of PB01 and 2 Gy has a synergic effect on ER stress and apoptotic cell death in A549 and H460 cells when compared to cells treated with PB01 or 2 Gy, alone (Fig. 7e). PB01 or PB01/2 Gy treatments cause lower levels expression of p-PERK, p-eIF2ɑ, ATF4, p-IRE1ɑ, p-JNK, CHOP, and cleaved caspase-3 expression in A549R and H460R cells when compared to A549 and H460 cells, but treatment with PB01/2 Gy induces the expression of p-PERK, p-eIF2ɑ, ATF4, p-IRE1ɑ, p-JNK, CHOP, and cleaved caspase-3 in A549R and H460R (Fig. 7e). These findings suggested that PB01, in combination with radiation, causes radio-resistant NSCLC cells to become radiation-sensitive via ER stress, which induces apoptotic cell death.

### The inhibition of ER stress attenuates PB01-mediated apoptotic cell death in radio-resistant NSCLC cells

**Figure 8:**
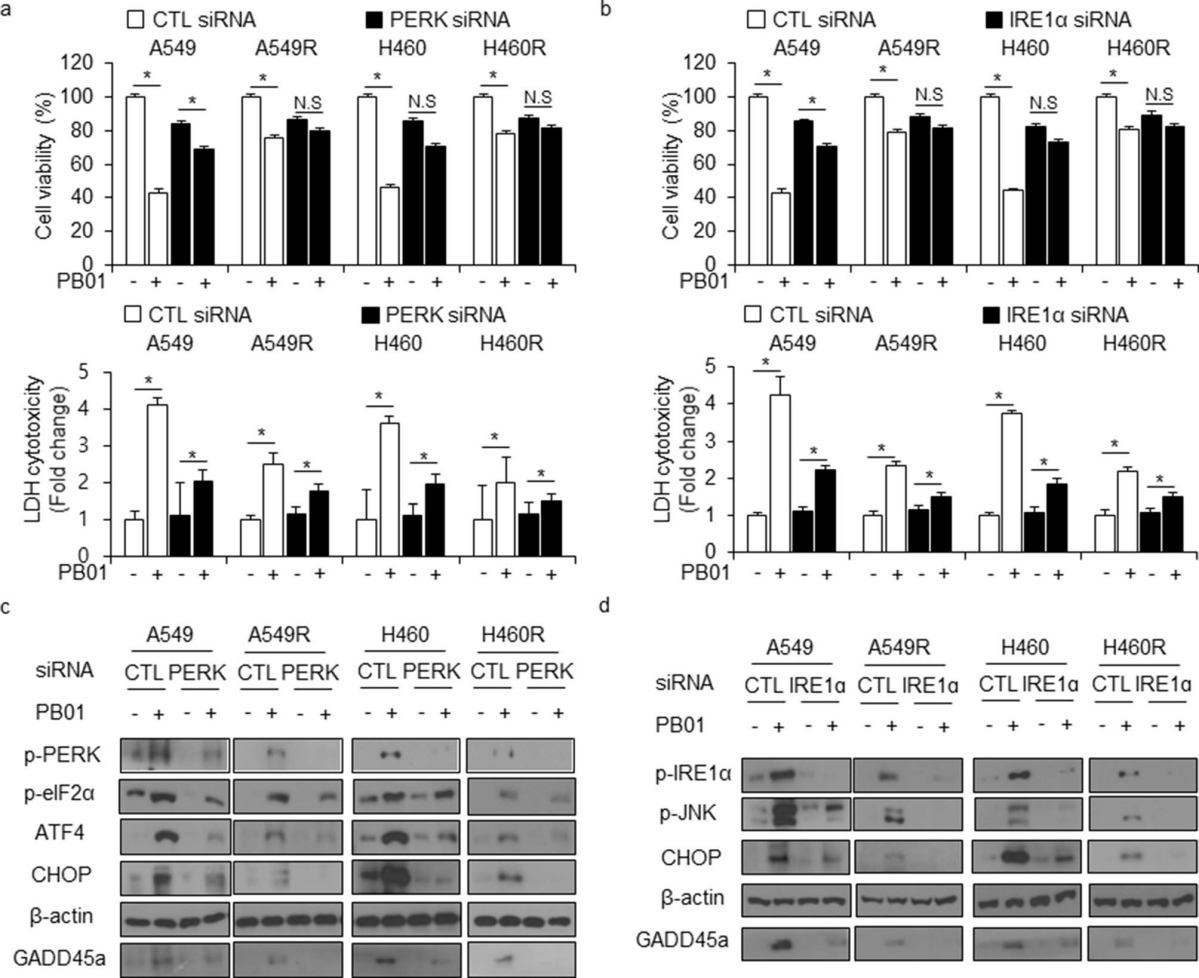
Inhibition of ER stress blocks cell death caused by PB01/radiation treatments in radiation-resistant NSCLC cells. (**a**–**d**) After A549, A549R, H460 and H460R cells were transfected with PERK and IRE1ɑ siRNAs, a cell viability assay and an LDH assay were performed as well as Western blot analysis examining p-PERK, p-eIF2ɑ, ATF4, p-IRE1ɑ, p-JNK, CHOP and GADD45α levels after PB01 (100 nM, 24 h) treatment; *p < 0.05. β-actin was used as the protein loading control.

To determine if ER stress regulates PB01-mediated apoptotic cell death in radio-resistant NSCLC cells, we treated PERK and IRE1ɑ knockdown A549, A549R, H460, and H460R cells (created using specific siRNAs) with PB01. Our results indicate that PB01 caused greater chemo-resistance in A549R and H460R cells than in A549 and H460 cells. It also increased cell viability and decreased of LDH cytotoxicity in PERK and IRE1ɑ knockdown NSCLC cells and radio-resistant NSCLC cells when compared to cells transfected with control siRNA (Fig. 8a,b). To further probe whether ER stress causes cell death after PB01 treatment, we performed Western blot analyses. PB01 increased p-PERK, p-eIF2ɑ, ATF4, CHOP, p-IRE1ɑ, and p-JNK expression in control siRNA-transfected A549, A549R, H460, and H460R cells, but not in PERK or IRE1ɑ knockdown cells (Fig. 8c,d). Additionally, PB01 caused less ER stress activation in A549R and H460R cells than in A549 and H460 cells, and PB01 down-regulated GADD45ɑ expression in PERK and IRE1ɑ siRNA-transfected A549, A549R, H460, and H460R cells. These findings suggest that PB01 also induces ER stress-induced cell death in both NSCLC cells and radio-resistant NSCLC cells, and this cell death may be regulated by the p53-GADD45ɑ axis. When viewed together, our results indicate that PB01 regulates apoptotic and mitochondrial cell death via ER stress and ATR signaling in NSCLC cells (Figure S5).

## Discussion

IR is known as a double-edged sword: it kills cancer cells and slows growth by DNA damage, but it can also cause distant metastases, and induce radio-resistance and damaged normal tissues. Recently, EMT has been found to induce malignant characteristics in cancer cells, causing metastatic dissemination, resistance to various therapies, and recurrence^45^. Resistance to radio-therapeutics represents the biggest obstacle in the therapy for patients with NSCLC, and the presence of EMT phenotypic cells can explain, at least partially, the development of NSCLC radio-resistance and the high mortality rate among NSCLC patients^46^. Accumulating evidence indicates that radiation is one of the causes of EMT; therefore, it is necessary to understand the relationship between radiation and EMT, the characteristic of novel targets that can fight cancer progression, and damaged normal tissue induced by radiotherapy^47^. A thorough understanding of the mechanisms through which residual tumor cells survive after radiotherapy could ultimately provide new, more effective radio-therapeutic strategies. Therefore, we generated A549R and H460R radio-resistant subclones by treating A549 and H460 cells with multiple fractions of gamma-radiation and characterized their radio-resistant phenotypes. In this study, we used parental and radiation-resistant human lung cancer cell lines (A549 and H460) to investigate the molecular mechanisms and the cellular behaviors associated with PB01 in combination with radiation. We also demonstrated that A549R and H460R cells underwent EMT. This was confirmed by changes among the EMT regulating proteins: decreased expression of E-cadherin and increased expression of vimentin, Snail, Slug, and N-cadherin, correlating with a significant increase in the expression of EMT regulating transcription factors and by the enhanced invasive and migratory ability of the cells. In agreement with our findings, an EMT-like phenotype and its close relationship with radio-resistance have been investigated in various cancer cells^48–51^. Increasing reports indicate that EMT process indicating change to a mesenchymal phenotype regulates activation of the aggressive phenotype and metastatic cascade of tumors^52^.

In the present study, we investigated the ability of PB01 to reverse radio-resistance via EMT, inhibit growth, and induce apoptotic cell death in A549R and H460R lung cancer cells. We showed that PB01 treatment reverses the radio-resistance via inhibition of EMT in A549R and H460R lung carcinoma cells, and that radio-sensitivity induces ER stress, the ATR-p53-GADD45ɑ axis, and mitochondrial cell death when used in combination with radiation (2 Gy). A combination of PB01 and radiation up-regulated E-cadherin expression and down-regulated N-cadherin, vimentin, Snail, and Slug expression in A549R and H460R cells when compared to control cells. Colony formation assays demonstrated that PB01 treatment in combination with radiation, reduced the survival fraction of A549R and H460 cells when compared to A549 and H460 cells. To identify the mechanism through which PB01/radiation treatment reverses radio-resistance, we examined the expression of the transcriptional repressors Snail and Slug. They play a vital role in tumor progression via EMT phenotype, including tumor cell invasion and metastatic cascade^53^. Our data show that combination of PB01 and 2 Gy strongly decreases N-cadherin, vimentin, Snail, and Slug expression and mediates E-cadherin expression in A549R and H460R cells. These results indicate that radio-resistance in A549R and H460R cells can be altered via EMT reversal and down-regulation of Snail and Slug by PB01/radiation treatment. Interestingly, PB01 not only overcomes radio-resistance in A549R and H460R cells, but it also restores their sensitivity to IR. Further work is necessary to uncover the exact mechanism that causes this phenomenon.

The present study also investigated the mechanism underlying PB01-induced EMT inhibition in radio-resistant NSCLC cells. Since PB01 dramatically increased cell death in NSCLC cells, we hypothesized that this phenomenon may be linked to EMT. Apoptosis signaling, which includes ER stress, cell cycle arrest, and mitochondrial cell death, regulates the loss of the EMT phenotype in cancer^54–55^. Our data demonstrate that PB01 induces NSCLC cell death in several ways, including PERK and IRE1ɑ-mediated ER stress, ATR-p53-GADD45ɑ-induced cell cycle arrest, and BAX-induced mitochondrial cell death. PB01 decreases cell viability, increases of LDH release, increases caspase-3 and -9 activities through phosphorylation of ATR and p53 in A549 and H460 cells, and up-regulates the expression of GADD45ɑ, which indicates DNA damage. In an ATR knockdown experiment, PB01 treatment inhibits p53 phosphorylation and GADD45ɑ expression via ATR inhibition in A549 and H460 cells, and it inhibits apoptotic cell death by increasing cell viability and decreasing LDH cytotocixity. In cytosol and mitochondria fractioned samples, PB01 induces cytochrome c release in the cytosol fraction, but it mediates BAX expression in the mitochondrial fraction, which indicates PB01-induced mitochondrial cell death. PB01 also induces ER stress-mediated cell death in NSCLC cells. PB01 treatment phosphorylates eIF2ɑ via phosphorylation of PERK, and it activates CHOP-mediated cell death by inducing ATF4. Furthermore, PB01 phosphorylates JNK via IRE1ɑ phosphorylation and it causes cell death by inducing CHOP. Additionally, PB01 activates ER stress-induced cell death in radio-resistant NSCLC cells, (A549R and H460R), but not when compared to in NSCLC cells, (A549 and H460).

ROS plays a key role in the cell death signaling of cancer cells, which includes DNA damage, redox balance, ER stress, mitochondria, death ligand receptor, cell cycle arrest, and the ATR-p53 axis^56^. In the present study, we demonstrated that PB01 induces ROS production using FACS with DCFDA dye-stained NSCLC cells. DPI, (a NOX inhibitor), suppresses ROS generation in PB01-treated NSCLC cells, but not Apo (a ROS inhibitor). DPI has frequently been used to inhibit ROS production in the presence of DPI through various flavoenzymes such as NAD(P)H oxidase (Noxs). Noxs are important molecules that regulate intracellular ROS release, and PB01-mediated regulation of ROS via NOXs may be an important factor for various cell death signals, including cell cycle arrest, DNA damage, mitochondrial cell death, and ER stress. Taken together, PB01 produces ROS via NOXs, and it triggers cell death via multiple signaling pathways in NSCLC cells and radio-resistant NSCLC cells.

In summary, PB01, in combination with radiation, overcomes acquired radio-resistance via inhibition of EMT and induction of Noxs-mediated ROS production and cell death in NSCLC cells. Based on these findings, PB01, in combination with radiotherapy, may present a novel and more effective approach in treating lung cancer, especially for patients who have acquired resistance to radiotherapy.

## Supplementary Information

Supplementary Information.
