# Supplementary material for: PB01 suppresses radio-resistance by regulating ATR signaling in human non-small-cell lung cancer cells
Source: Sci Rep. 2021 Jun 8;11:12093. doi: 10.1038/s41598-021-91716-z (PMC8187425; doi:10.1038/s41598-021-91716-z)

Supplementary materials:

PB01 suppresses radio-resistance by regulating ATR signaling in human non-small-cell lung cancer cells

Tae Woo Kim, Da-Won Hong, and Sung Hee Hong*

Division of Radiation Biomedical Research, Korea Institute of Radiological and Medical Sciences, Seoul 139-706, Korea.

* To whom correspondence should be addressed:

Sung Hee Hong *, Division of Radiation Biomedical Research, Korea Institute of Radiological and Medical Sciences, Seoul 139-706, Korea. Tel: +82-2-970-1320; Fax: +82-2-970-2402; E-mail: gobrian817@daum.net

Figure S1. Synthesis of PB01.

Step 1.

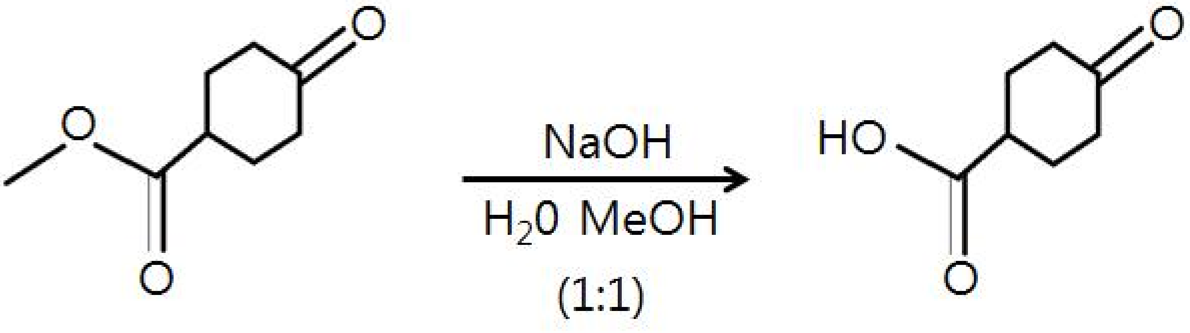

(II) (III)

Step 1. A sodium hydroxide aqueous solution (2.5 g, 43.5 mmol) was added to a solution of ethyl-4-cyclohexanone carboxylate (5 g, 29 mmol) in methanol (II). The resulting reaction mixture was stirred at room temperature for the reaction. After completion of the reaction, the solvent was removed under vacuum to obtain a crude product, which was then dissolved in water, and adjusted to a pH of 3 to 4 with hydrochloric acid, followed by extraction with ethyl acetate, drying with sodium sulfate (Na_2SO_4), and then concentration. The resulting crude product was purified using silica gel column chromatography to obtain 3.5 g of 4-cyclohexanone carboxylic acid in white solid as a compound of Formula III (Yield: 85%).

Step 2.

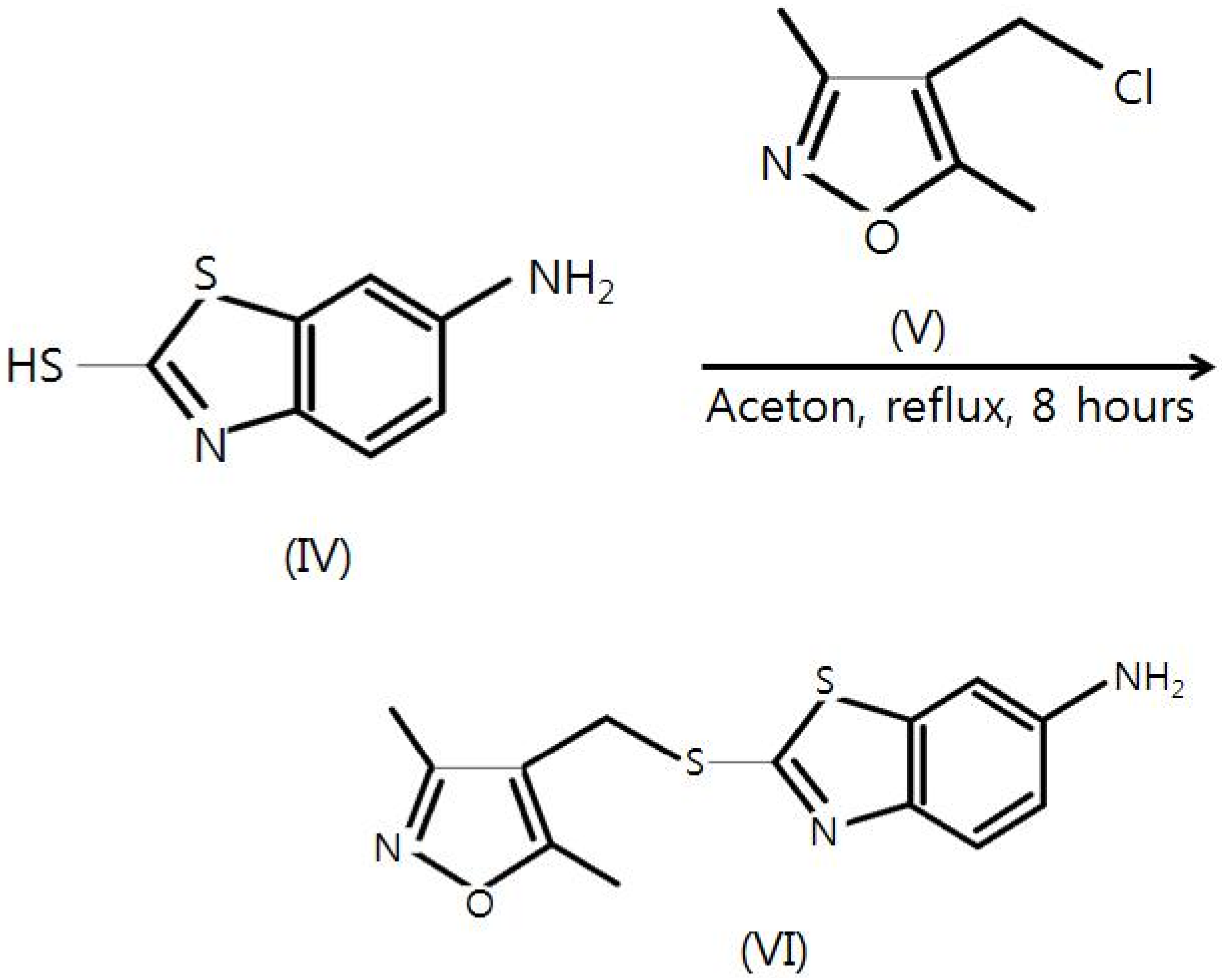

Step 2. 6-amino-2-mercapto benzothiazole (1 g, 5.49 mmol, IV), 4-(chloromethyl)-3,5-dimethylisoxazole (0.749 mL, 6.04 mmol, V), and K_2CO_3 (1.8 g. 13.72 mmol) were refluxed in acetone for about 8 hr. After completion of the reaction, then solvent was removed by rotary evaporation to obtain a crude product, which was then dissolved in water, followed by extraction with ethyl acetate, washing with sodium hydrogen carbonate, drying with sodium sulfate (Na_2SO_4), and then concentration. The resulting crude product was purified using silica gel column chromatography to obtain 1.07 g of a compound of Formula VI in white solid (Yield: 67%).

Step 3.

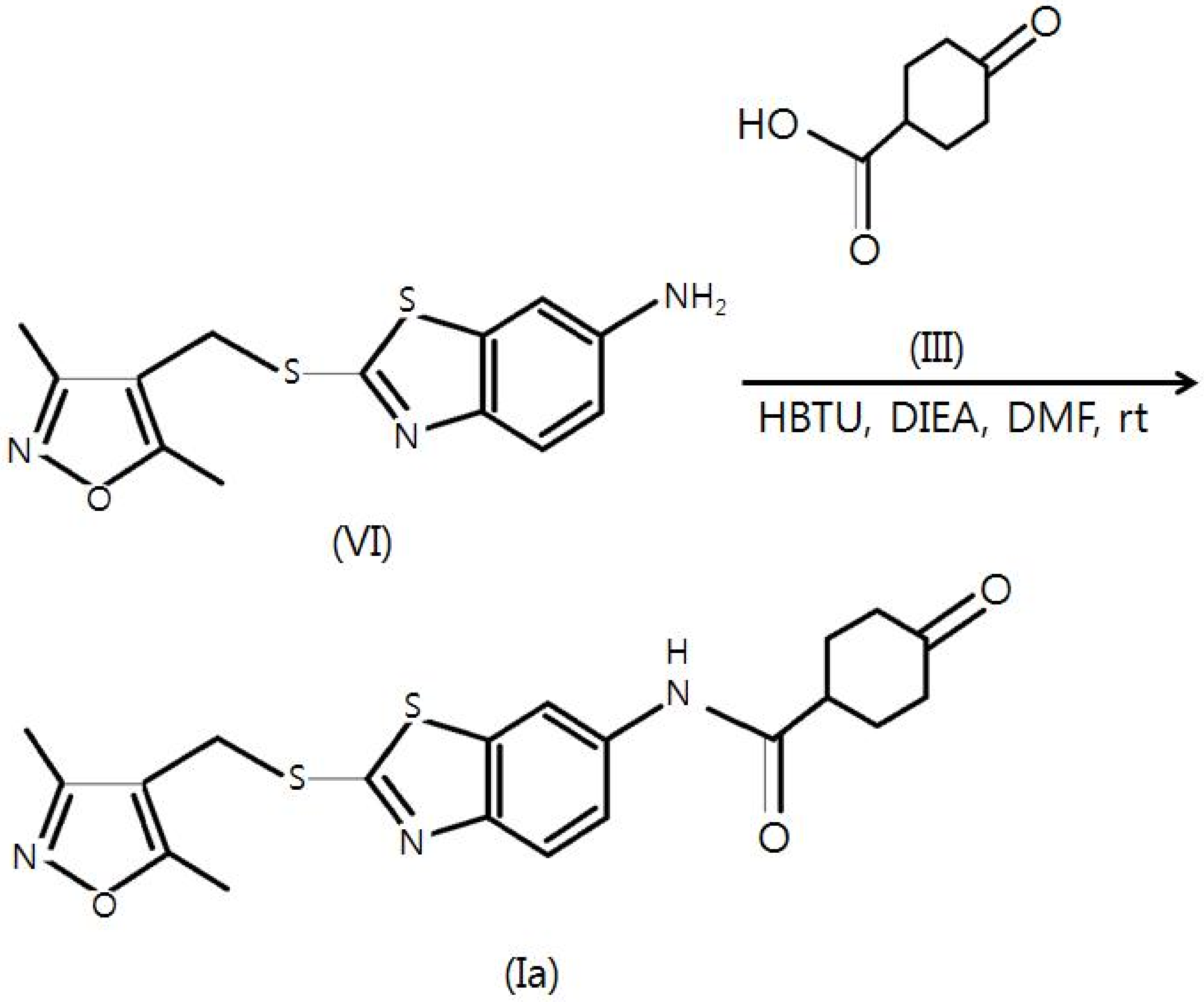

Step 3. Formula III (3.12 g, 21.9 mmol), HBTU (9.169 g, 24.1 mmol), and N,N-diisopropylethylamine (DIEA) (7.65 mL, 43.8 mmol) were added to a solution of Formula VI (6.4 g, 21.9 mmol) in dimethylformate (DMF). The resulting reaction mixture was stirred at room temperature overnight. After removing DMF under vacuum, the residue was diluted in ethyl acetate, washed with sodium carbonate and then brine, dried using anhydrous sodium sulfate, and then filtered. After removing the solvent under vacuum, the resulting crude product was purified using silica gel column chromatography to obtain 7 g 4-oxo-cyclohexanecarboxylic acid in white solid as a compound Formula Ia (Yield: 77%).

Step 4.

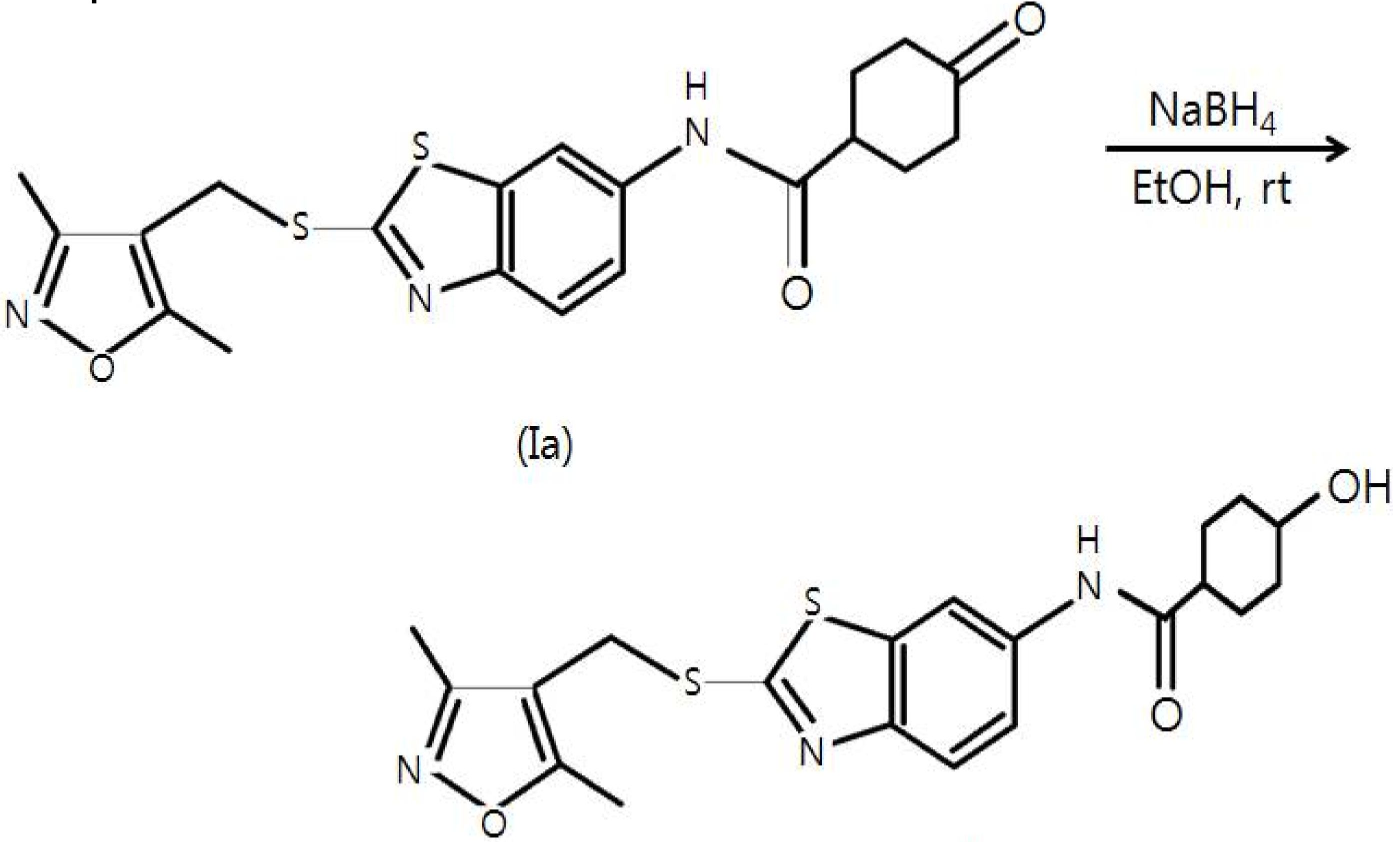

(Ia)

(Ib)

Step 4. $NaBH_4$ (0.76g, 2.02 mmol) was added to a solution of Fomula Ia (0.7 g, 1.68 mmol) in ethanol. The resulting reaction mixture was stirred at room temperature. After completion of the reaction, the reaction solvent was removed under vacuum to obtain a crude product. This crude product was dissolved in water, and pH -adjusted with 1N HCl to a pH 6 to 7. The resulting crude product was extracted with ethyl acetate, dried using sodium sulfate (Na_2SO_4), and then concentrated. The resulting crude product was purified using silica gel column chromatography to obtain 0.3 g of 4-hydroxy-cyclohexanecarboxylic acid in white solid as a compound of Formula Ib (Yield: 43%).

Step 5.

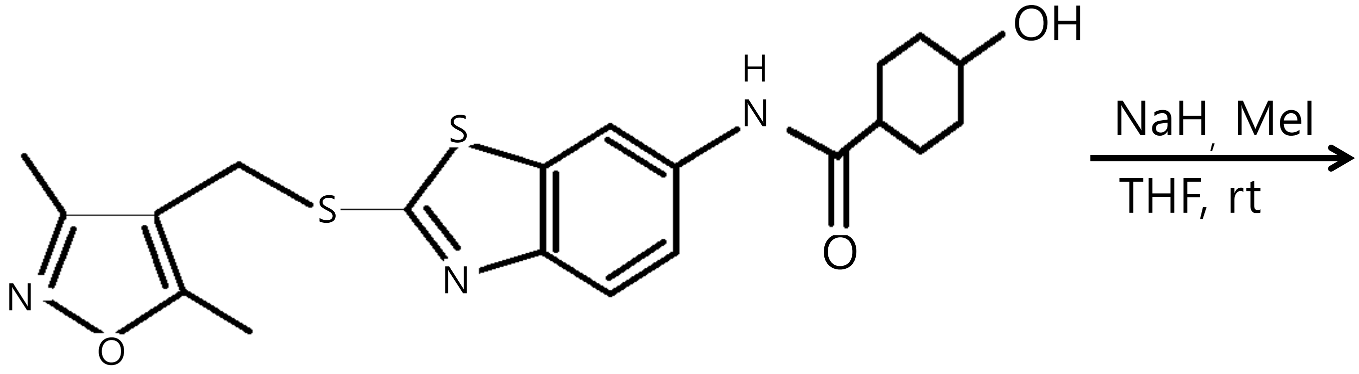

(Ib)

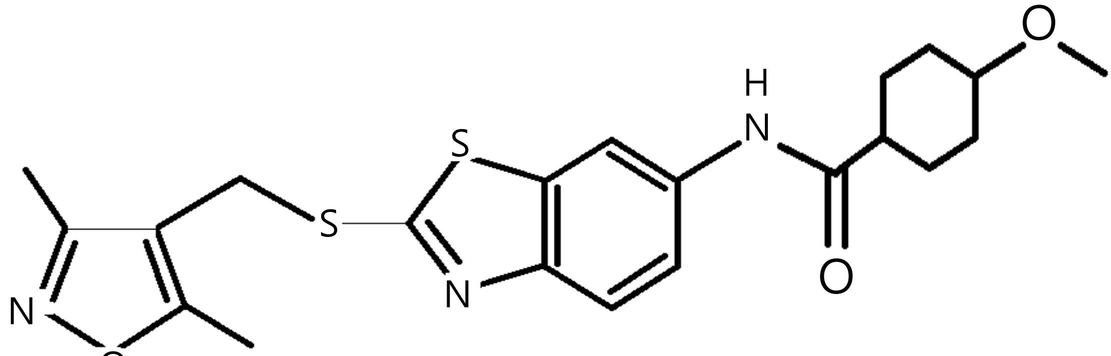

(PB01)

Step 5. A mixture NaH (0.021 g, 0.528 mmol), methyl iodide (0.029 mL, 0.48 mmol), and Formula Ib (0.2 g, 0.48 mmol) in tetrahydrofuran was stirred at room temperature. After completion of the reaction, the reaction solvent was removed by rotary evaporation, followed by dissolving the resulting mixture in water. The resulting mixture was extracted with ethyl acetate, washed with sodium hydrogen carbonate, dried suing sodium sulfate (Na_2SO_4), and then concentrated. The resulting crude product was purified using silica gel column chromatography to obtain 0.080 g of 4-methoxy-cyclohexanecarboxylic acid [2-(3,5-dimethyl-isoxazole-4-yl)sulfanyl-benzothiazole-6-ya]-amide in white solid as a compound of PB01 (Figure S1).

Figure S2. SB600125, a JNK inhibitor, inhibits cell death in PB01-treated NSCLC cells.

(A) Western blot analysis examining of p-JNK, p-ERK, p-p38, and p-AKT levels was performed in PB01 (100 nM, 0, 8, 16, and 24 h)-treated A549 and H460 cells. β-actin was used as the protein loading control. (B-D) A549 and H460 cells were pretreated with SP600125 (10 μM), U0126 (10 μM), and SB203580 (10 μM) for 4 h and subsequently treated with PB01 (100 nM, 24 h). Cell viability was determined using a WST-1 assay, and cell cycle arrest was observed using FACs. Western blot examining of cleaved caspase-3 levels was performed using these samples; *, $p<0.05$. β-actin was used as the protein loading control.

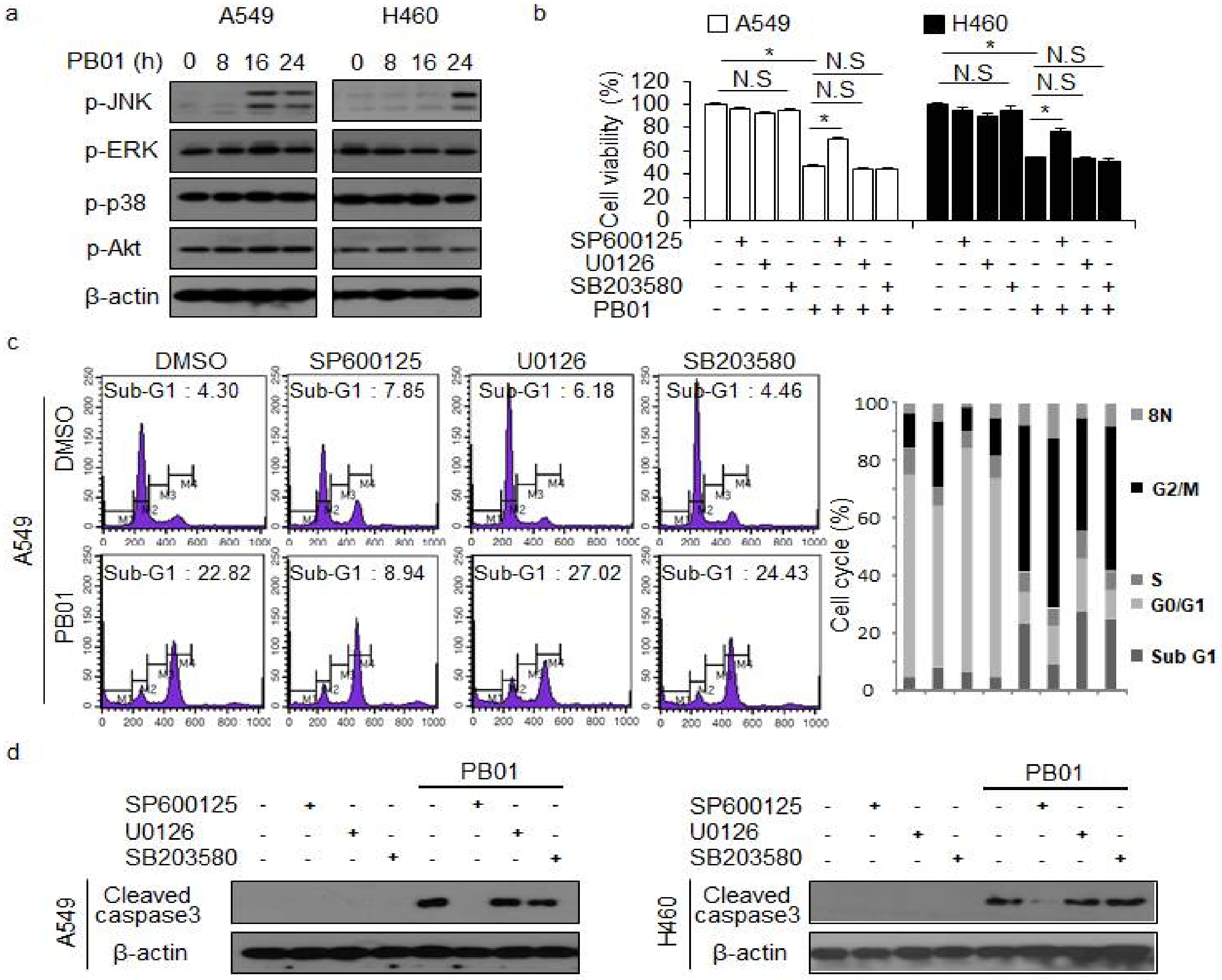

Figure S3. mathematical isobologram analyses (dose-effect curves) obtained from the CompuSyn software for PB01 (100 nM, 24h) and Gy (0, 2, 4 and 6, 24h) combinations in A549, A549R, H460 and H460R cells.

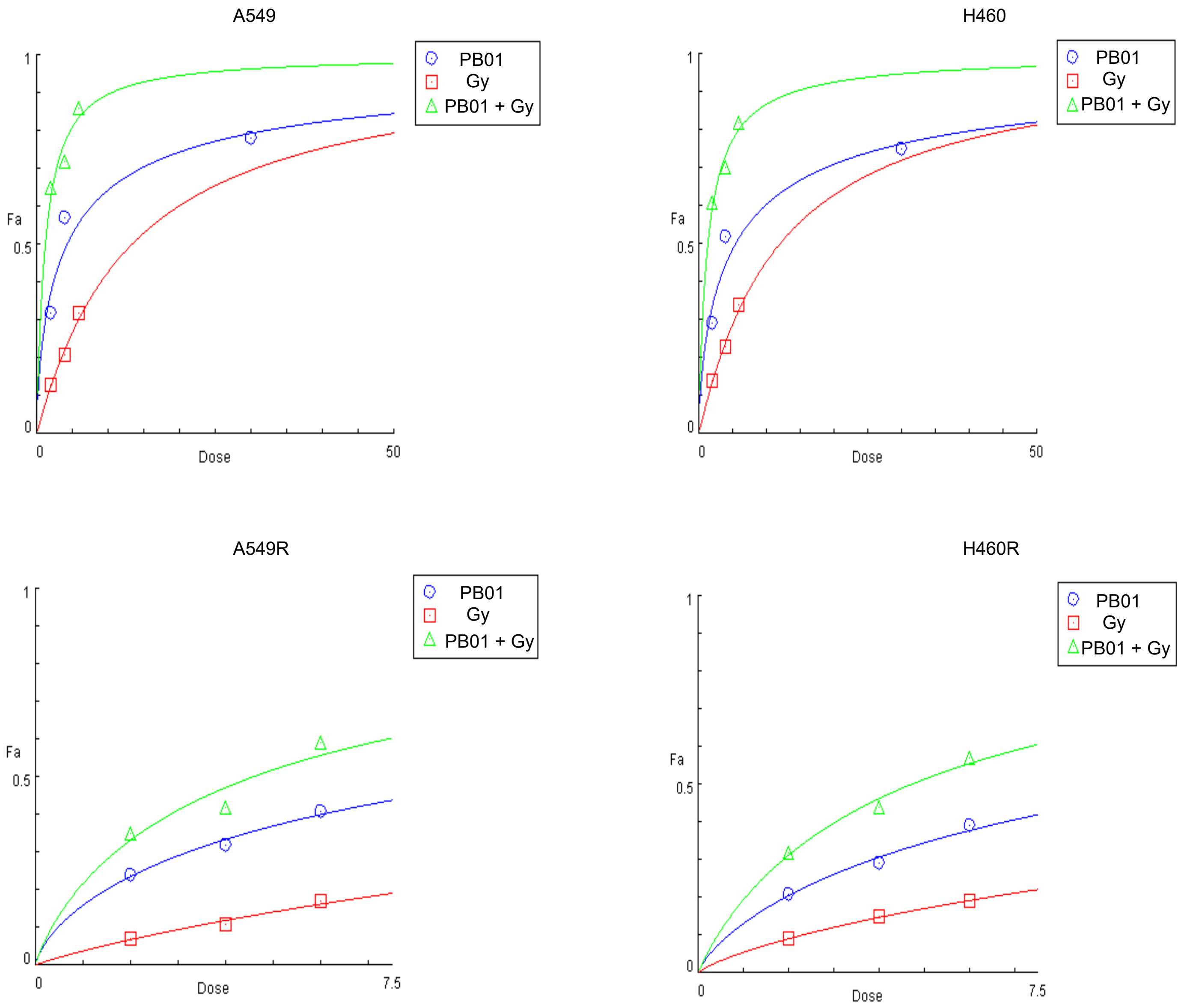

Figure S4. 2Gy in combination with PB01 inhibits EMT Phenotype.

Real-time RT-PCR was used to measure the expression of N-cadherin and vimentin in A549, A549R, H460 and H460R cells; *, $p<0.05$. β-actin was used as the RNA loading control.

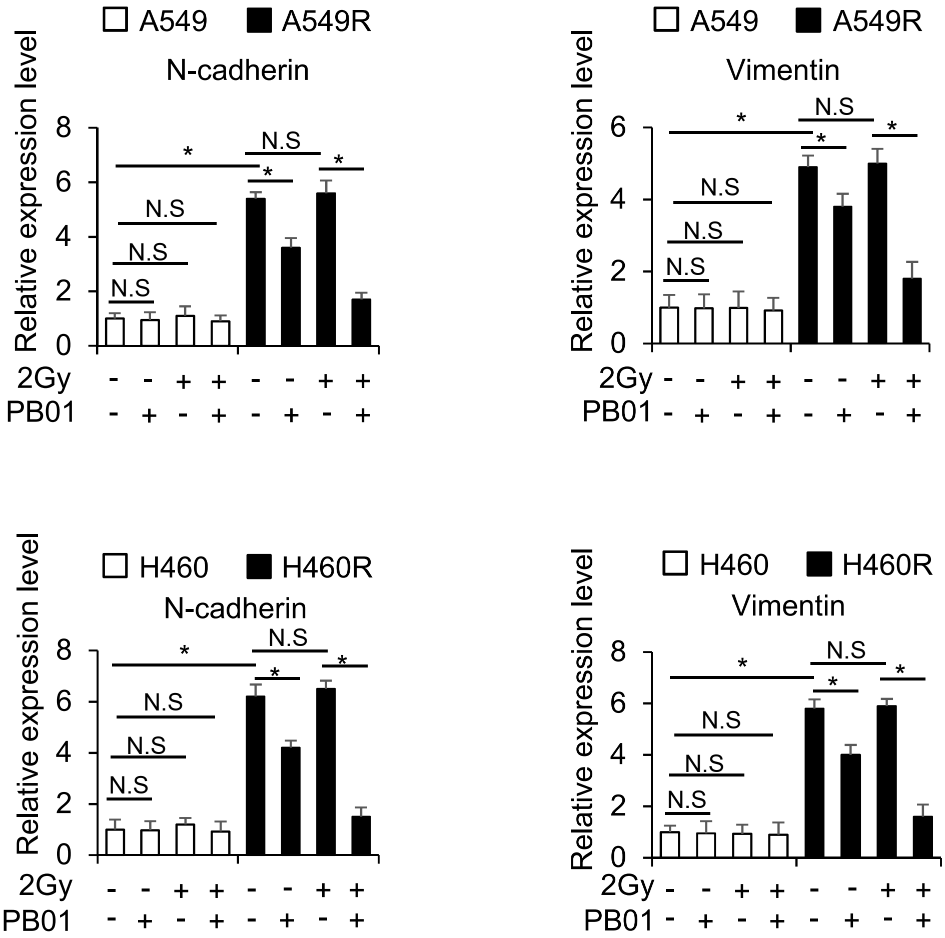

Figure S5. PB01 induces multiple ROS-mediated cell death pathways and overcomes resistance to radiotherapy.

Schematic representation of multiple PB01-induced cell death signaling pathways via ROS in NSCLC and radio-resistant NSCLC cells.

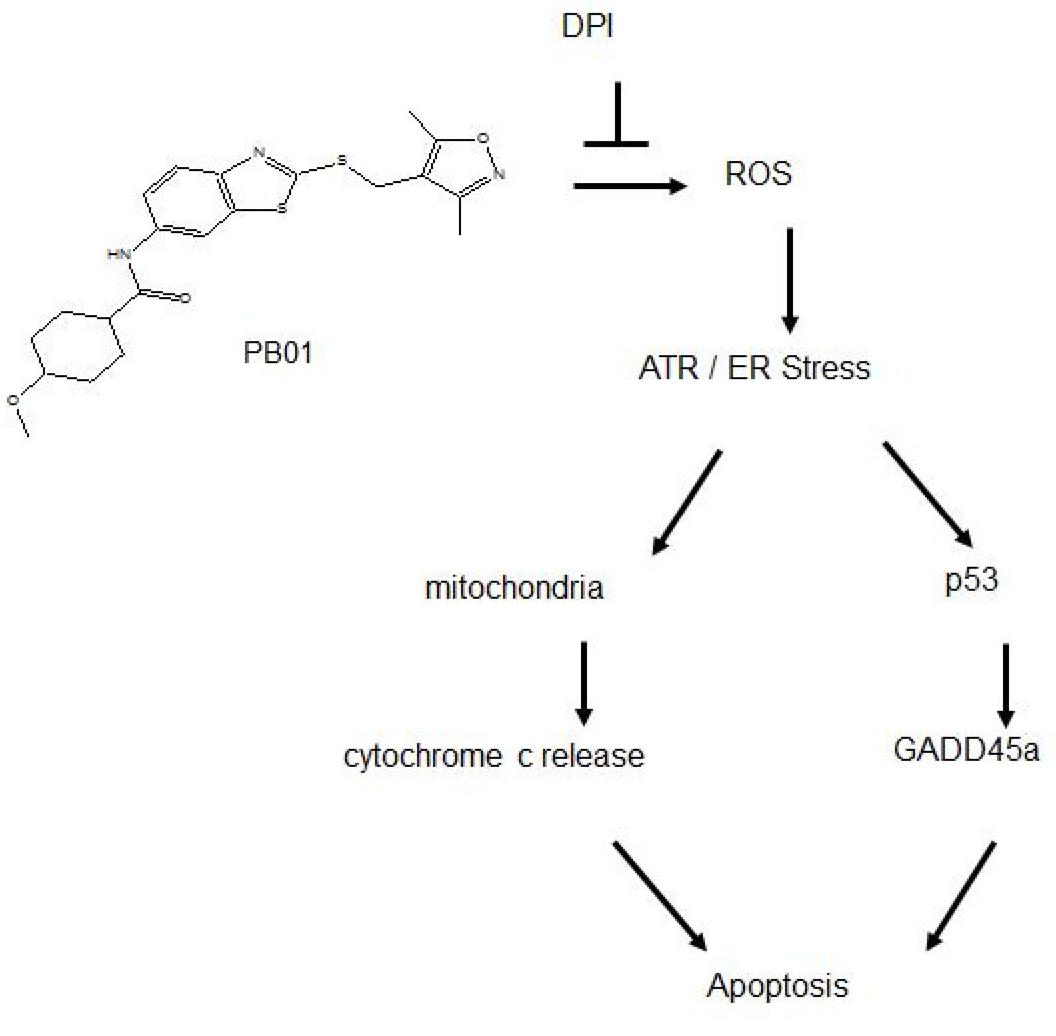

Figure S6-S23. Whole blots and intensity ratio of each band relative to the Western Blotting analyses.

Figure S6. Supplementary materials for Figure 2e

S6-1

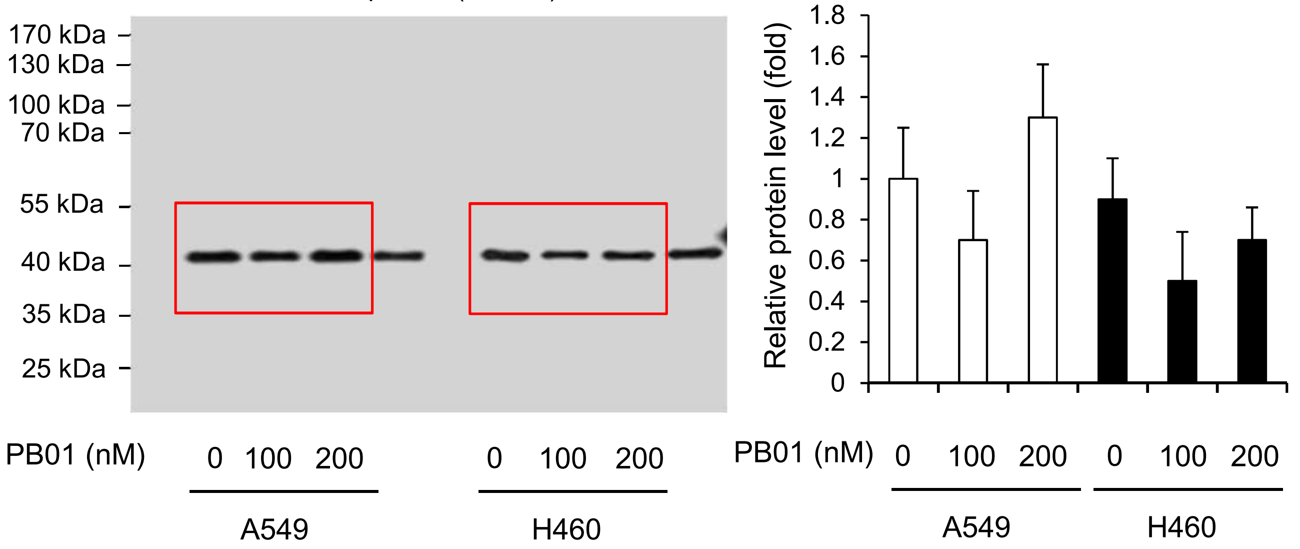

S6-2

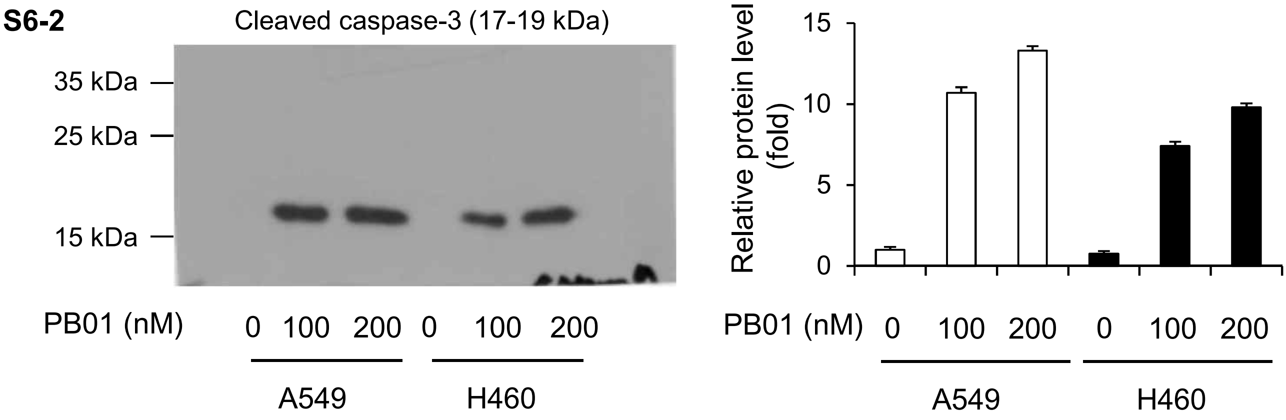

S6-3

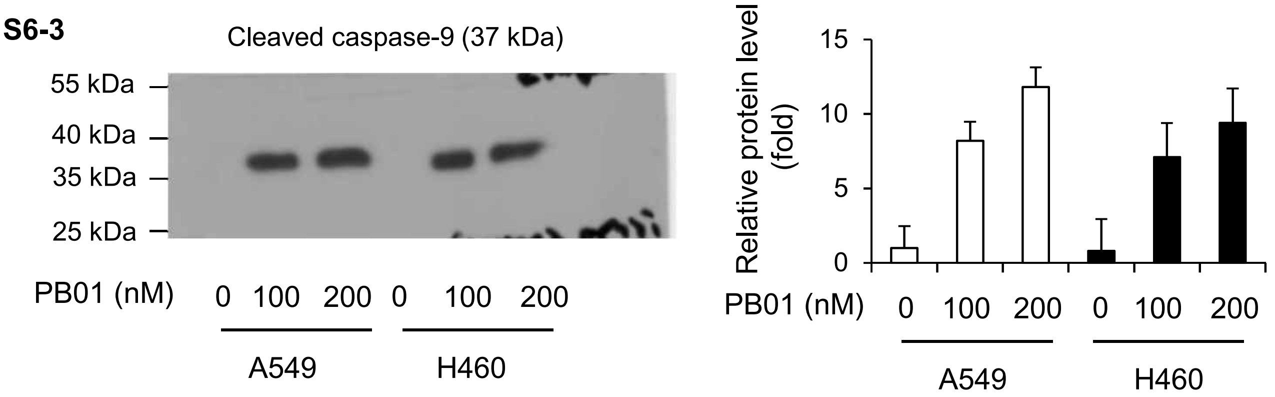

Figure S7. Supplementary materials for Figure 2h

S7-1 β-actin (43 kDa)

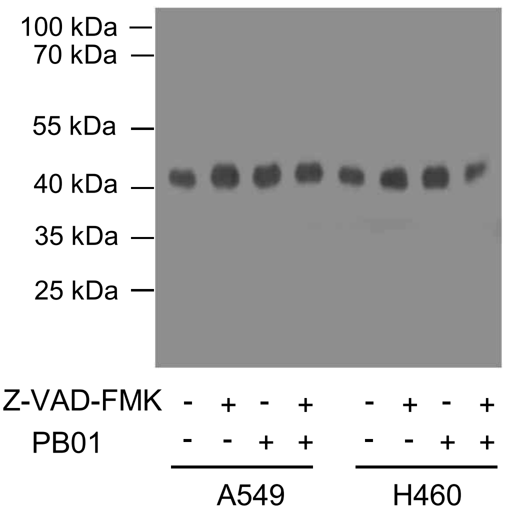

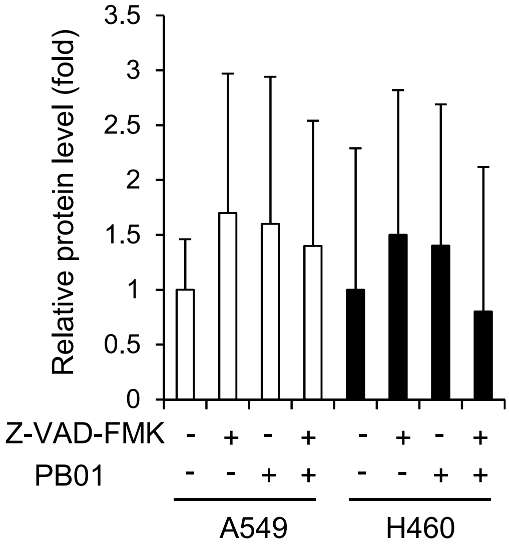

S7-2 Cleaved caspase-3 17-19 (kDa)

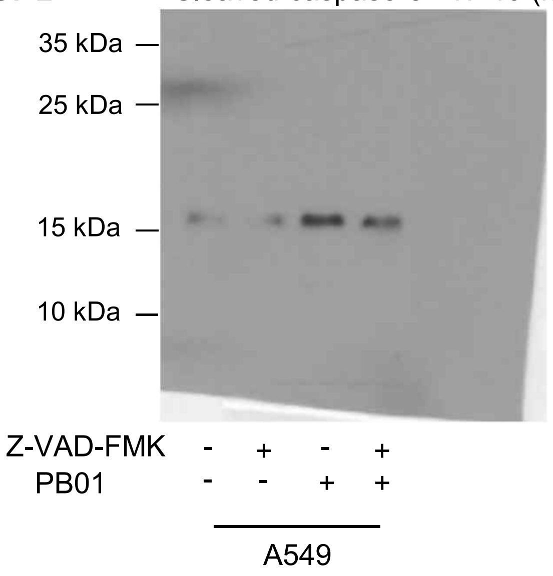

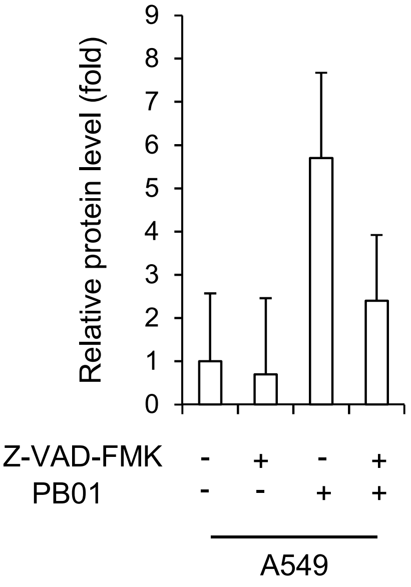

S7-3 Cleaved caspase-3 17-19 (kDa)

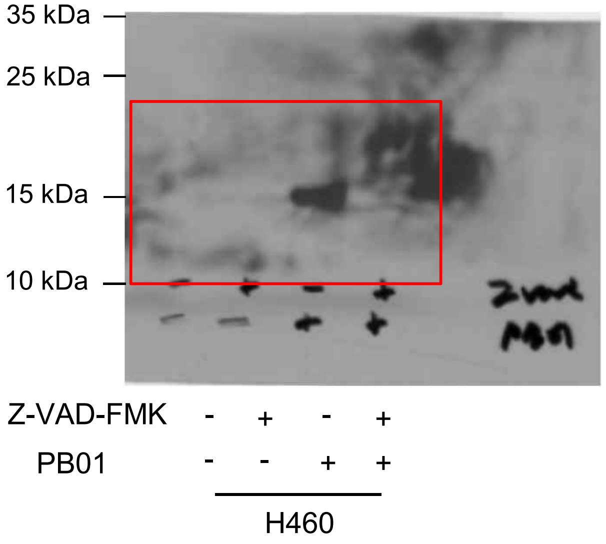

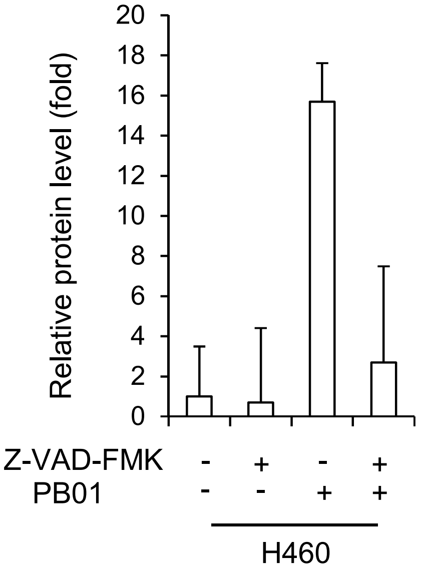

Figure S8. Supplementary materials for Figure 3a

S8-1

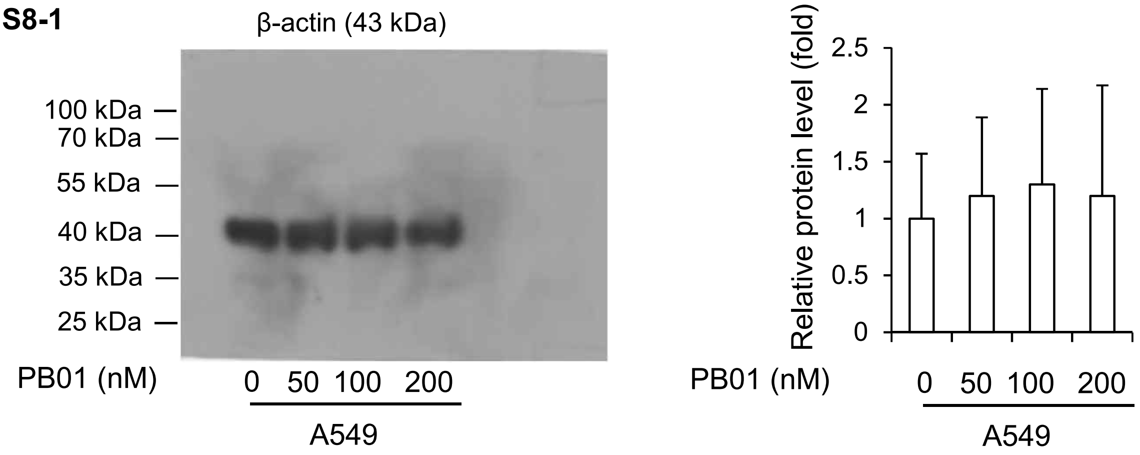

S8-2

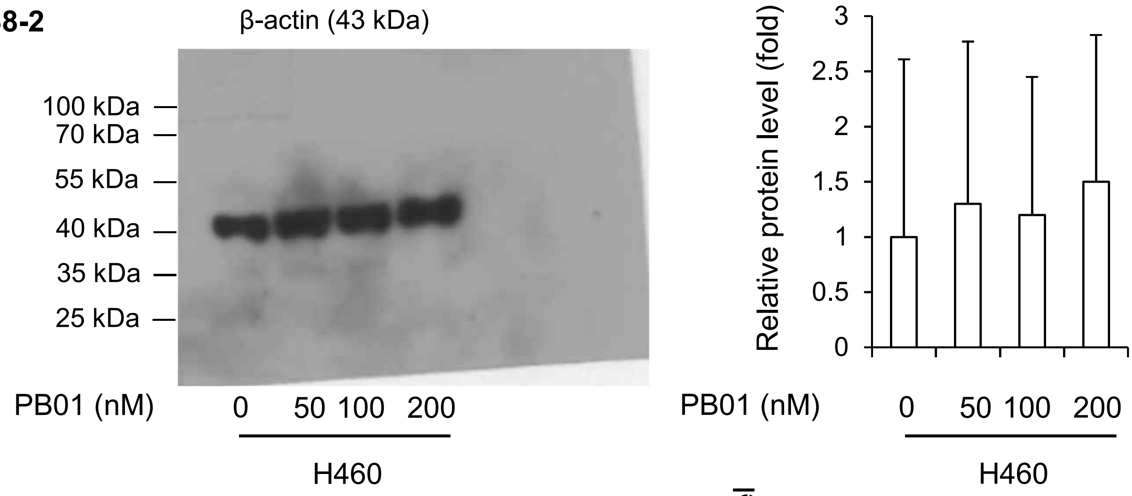

S8-3

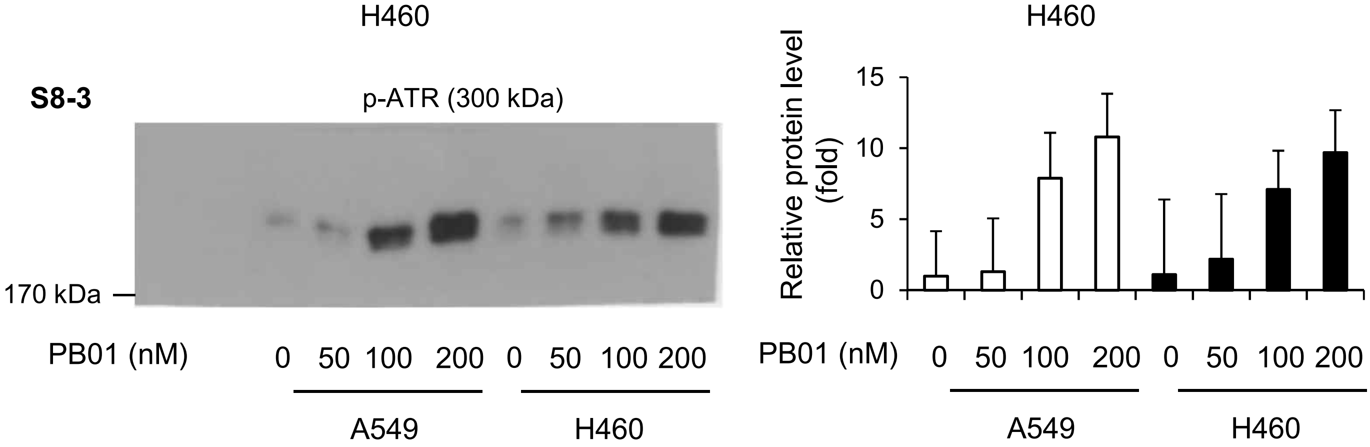

S8-4

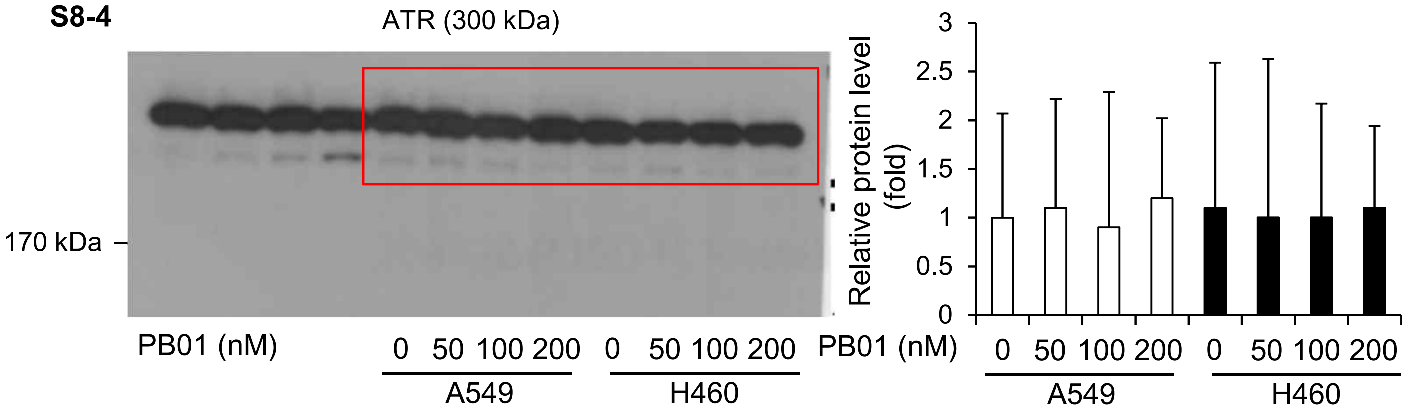

S8-6

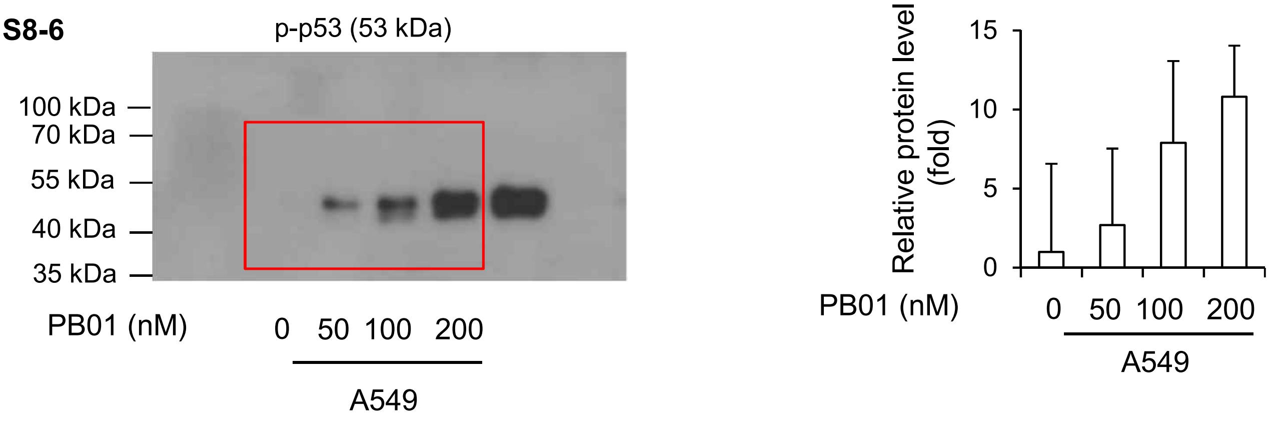

S8-7

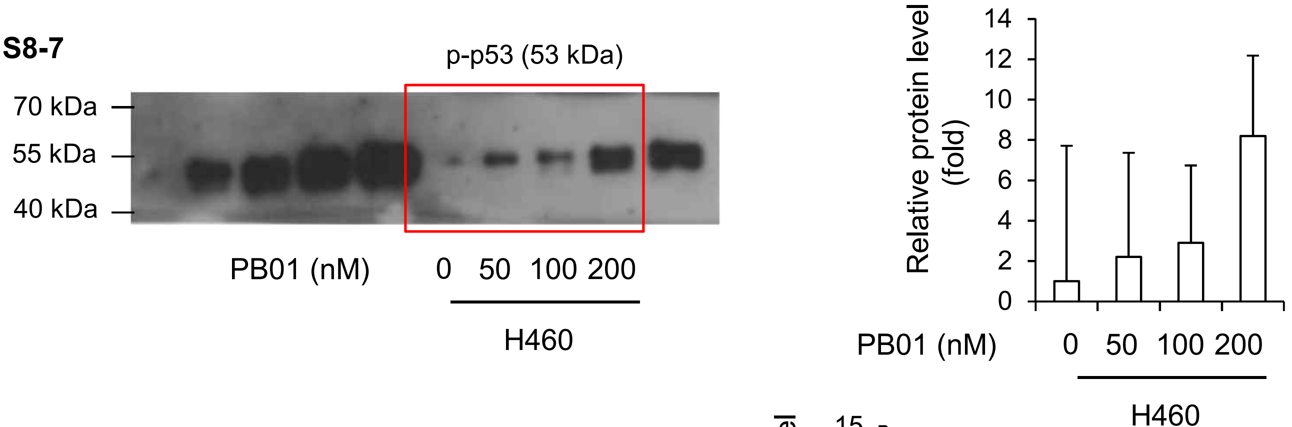

S8-8

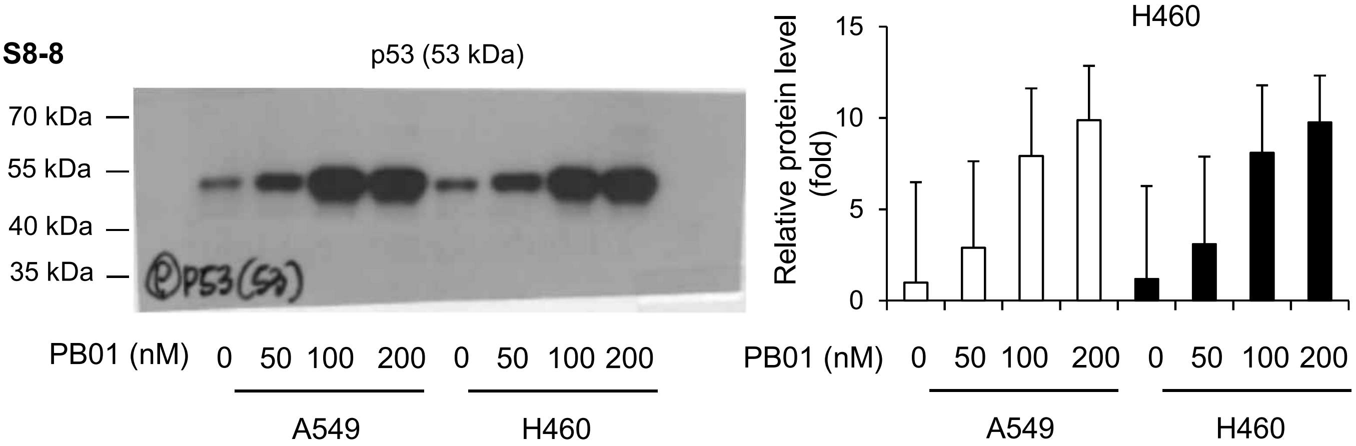

S8-9

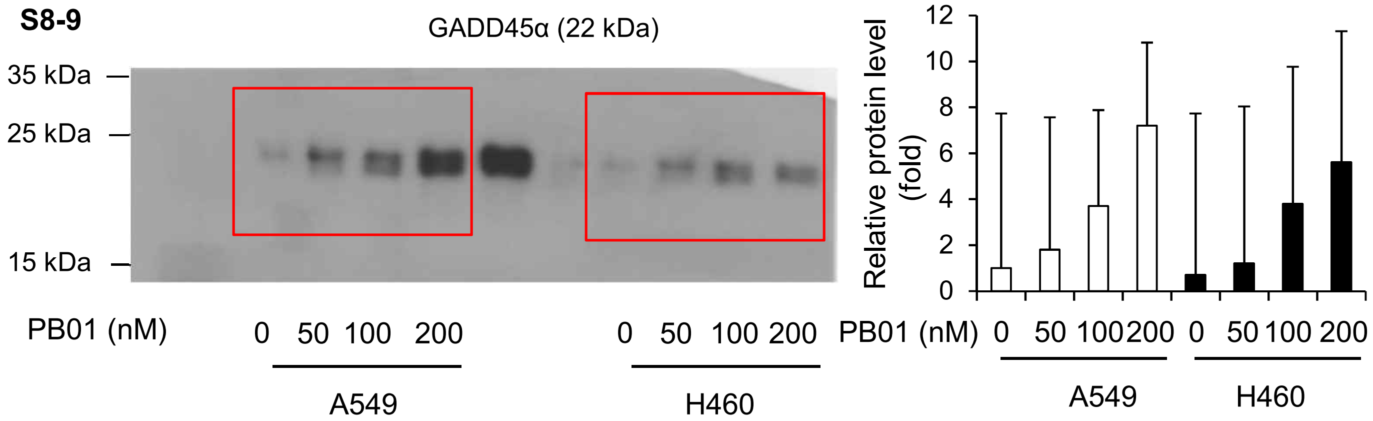

S8-10

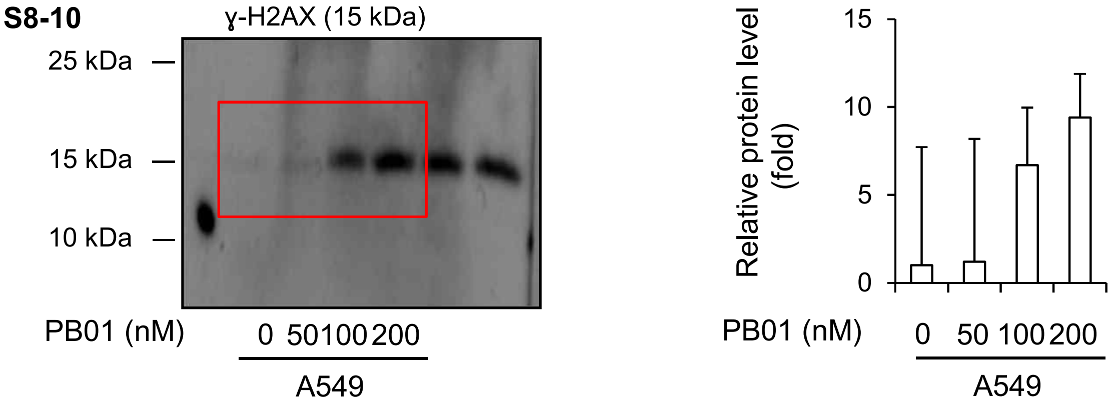

S8-11

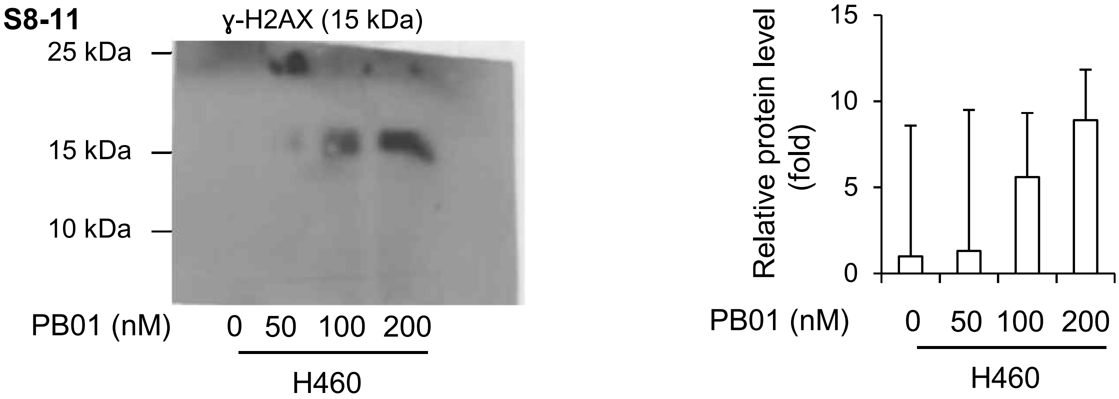

S8-12

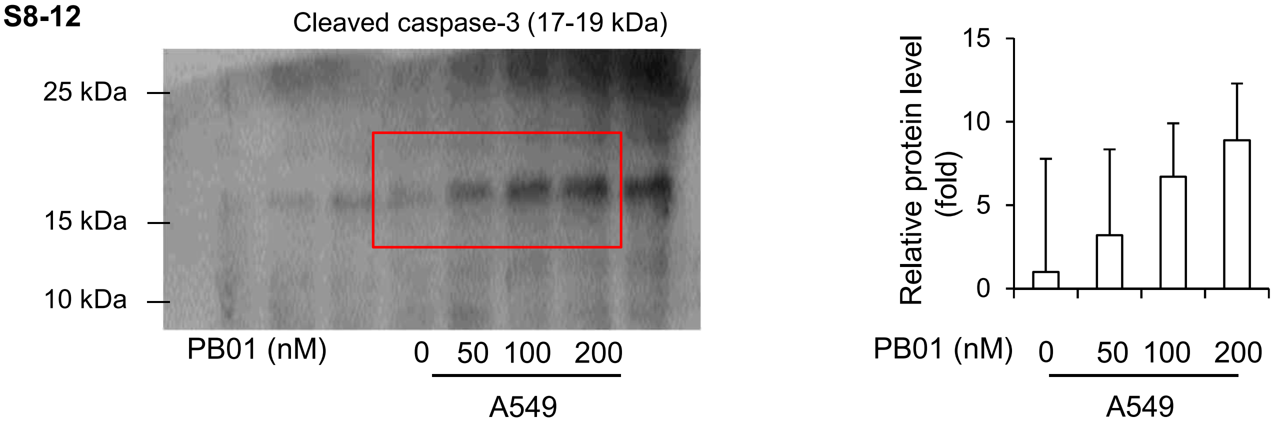

S8-13

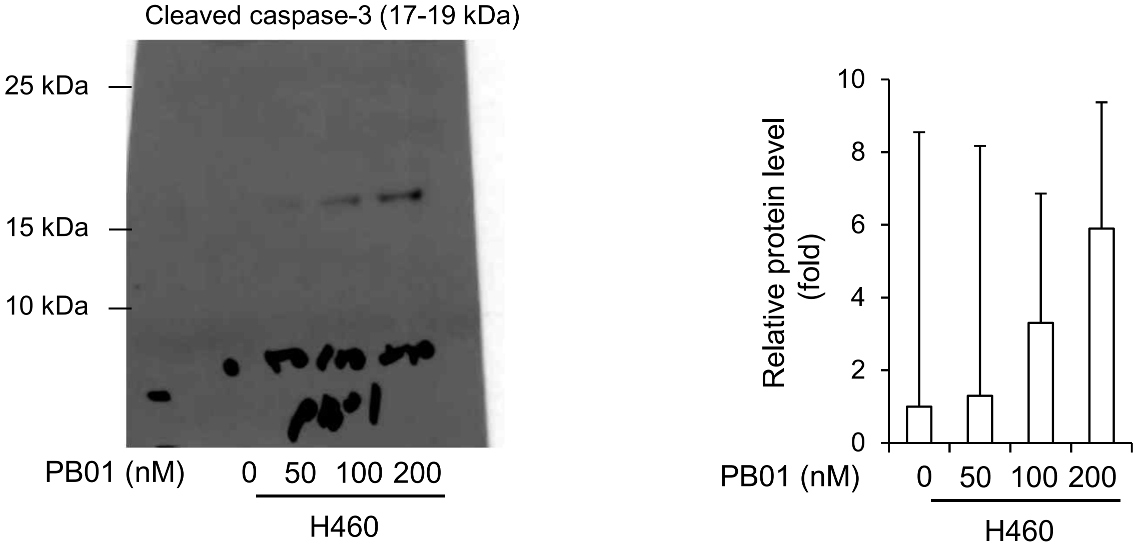

S8-14

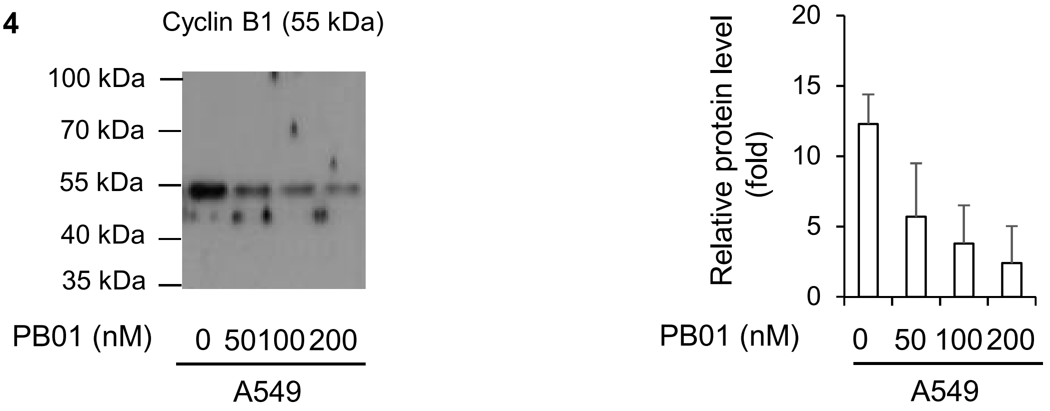

Cyclin B1 (55 kDa)
100 kDa
70 kDa
55 kDa
40 kDa
35 kDa
PB01 (nM)
0 50100200
A549
Relative protein level (fold)
20
15
10
5
0
PB01 (nM)
0 50 100200
A549

S8-15

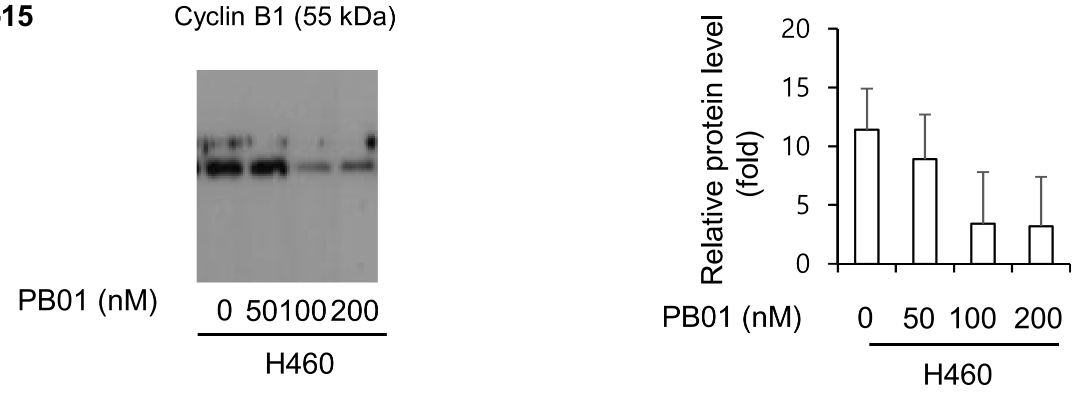

Cyclin B1 (55 kDa)
PB01 (nM)
0 50100200
H460
Relative protein level (fold)
20
15
10
5
0
PB01 (nM)
0 50 100 200
H460

Figure S9. Supplementary materials for Figure 3b

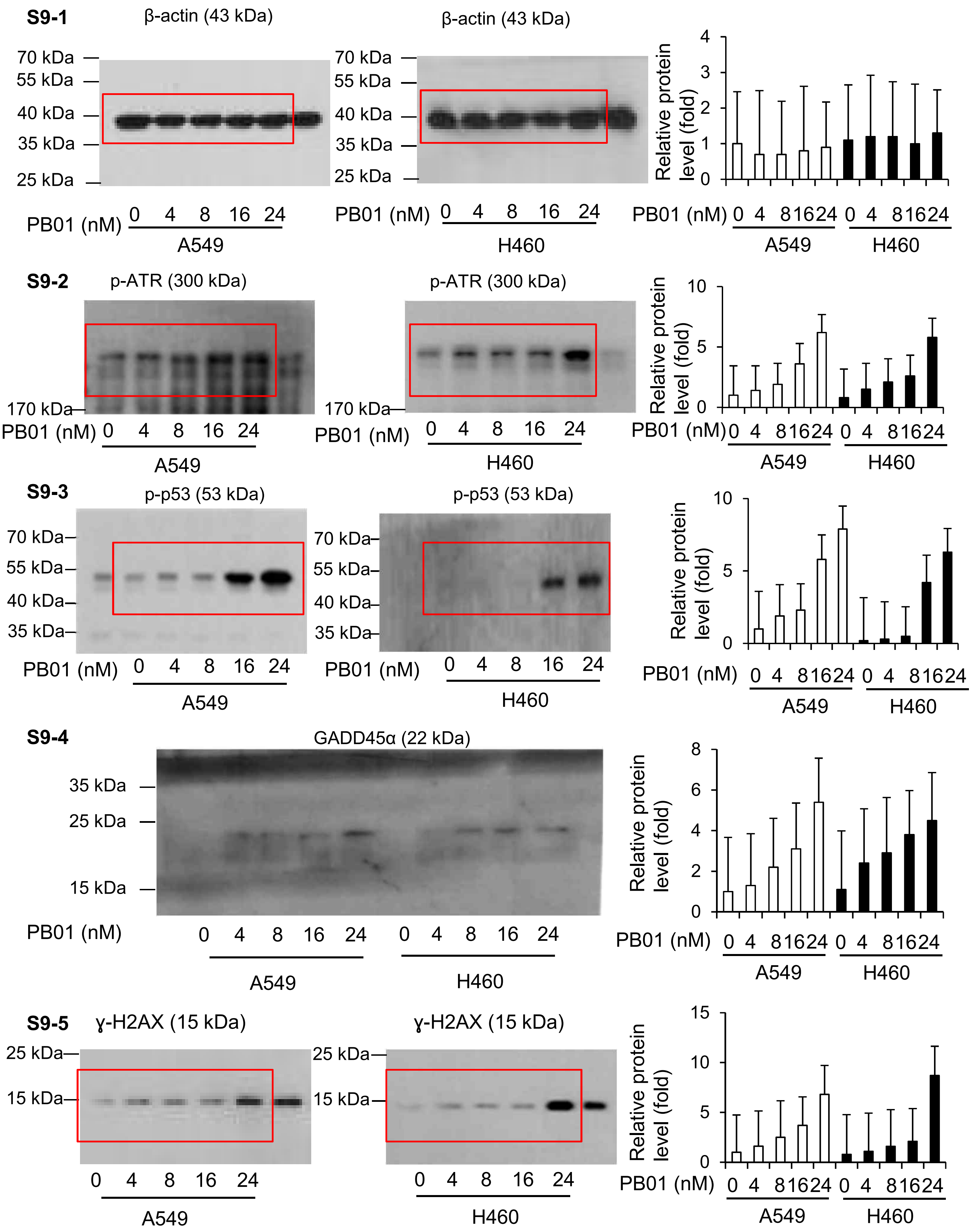

S9-6

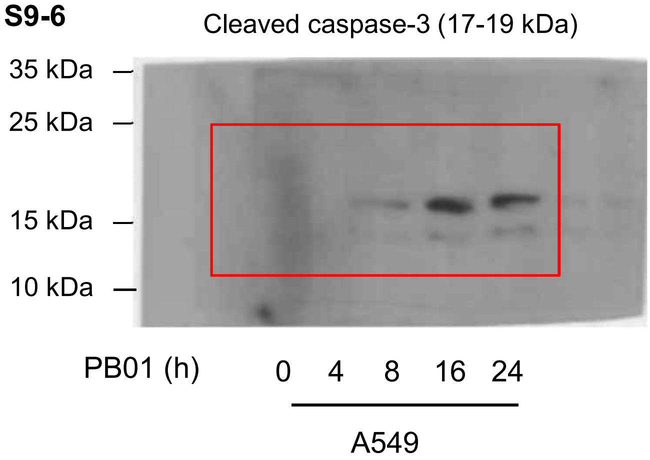
Cleaved caspase-3 (17-19 kDa)
35 kDa
25 kDa
15 kDa
10 kDa
PB01 (h)
0 4 8 16 24
A549

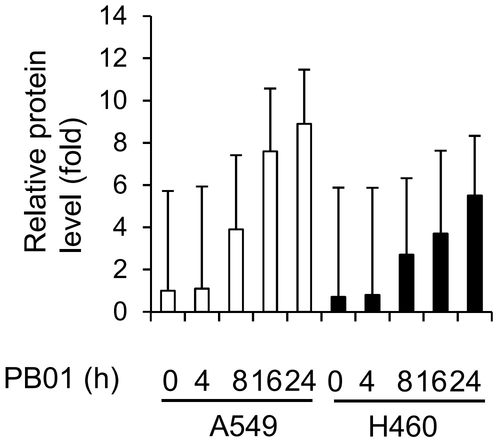
Relative protein level (fold)
14
12
10
8
6
4
2
0
PB01 (h)
0 4 8 16 24 0 4 8 16 24
A549
H460

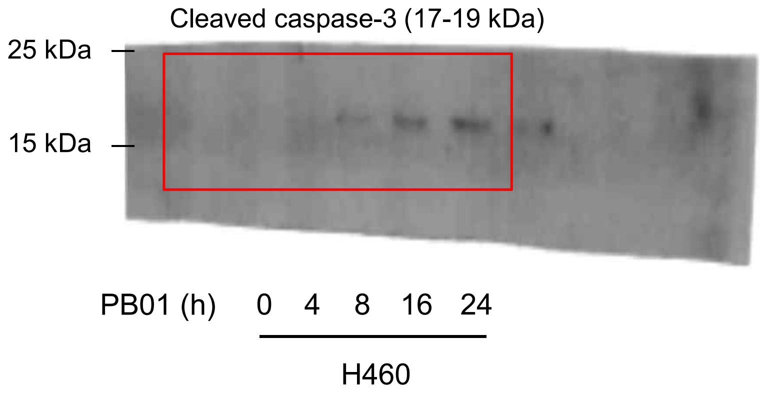
Cleaved caspase-3 (17-19 kDa)
25 kDa
15 kDa
PB01 (h)
0 4 8 16 24
H460

S9-7

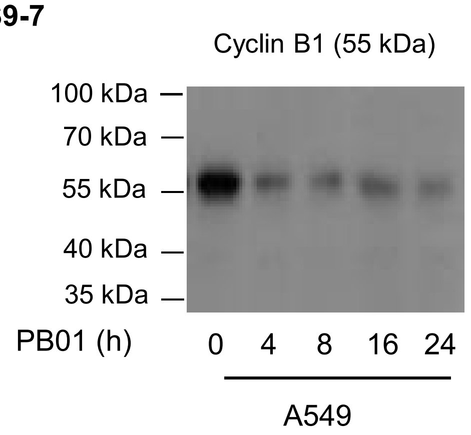
Cyclin B1 (55 kDa)
100 kDa
70 kDa
55 kDa
40 kDa
35 kDa
PB01 (h)
0 4 8 16 24
A549

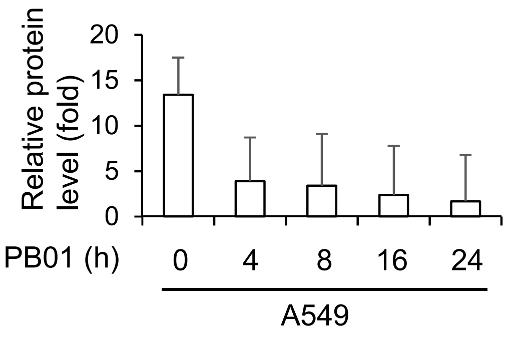
Relative protein level (fold)
20
15
10
5
0
PB01 (h)
0 4 8 16 24
A549

S9-8

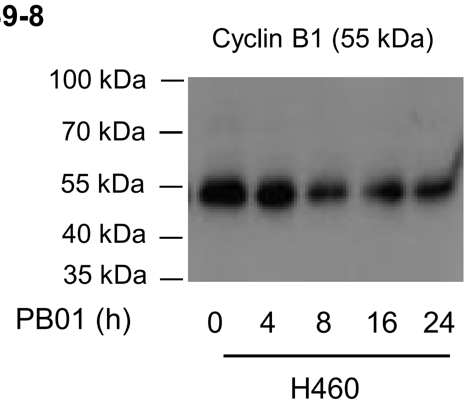
Cyclin B1 (55 kDa)
100 kDa
70 kDa
55 kDa
40 kDa
35 kDa
PB01 (h)
0 4 8 16 24
H460

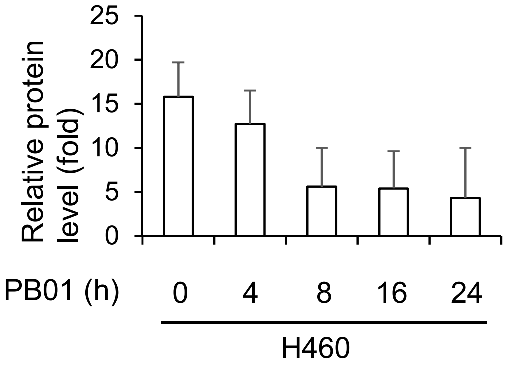
Relative protein level (fold)
25
20
15
10
5
0
PB01 (h)
0 4 8 16 24
H460

Figure S10. Supplementary materials for Figure 3f

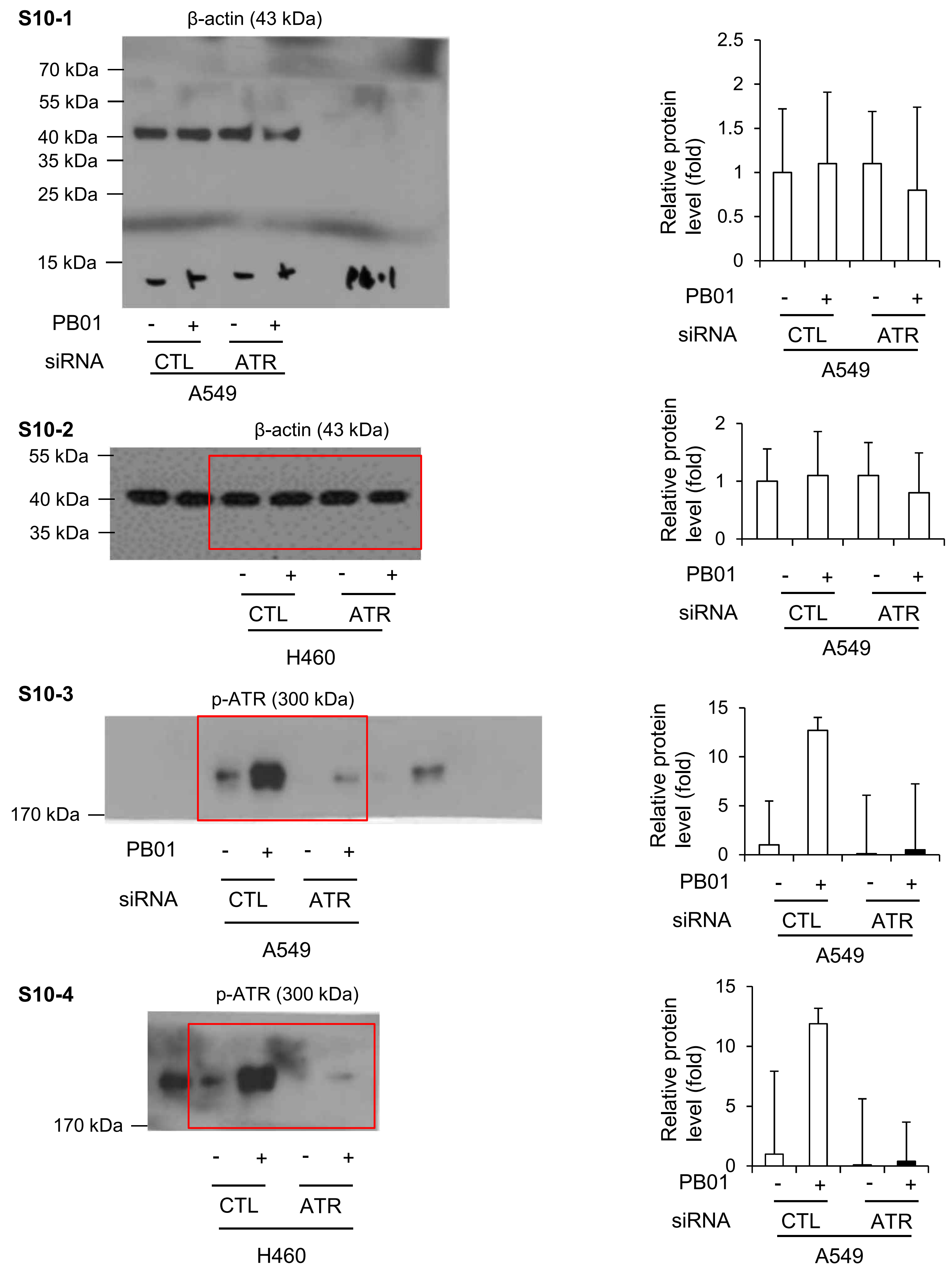

S10-5

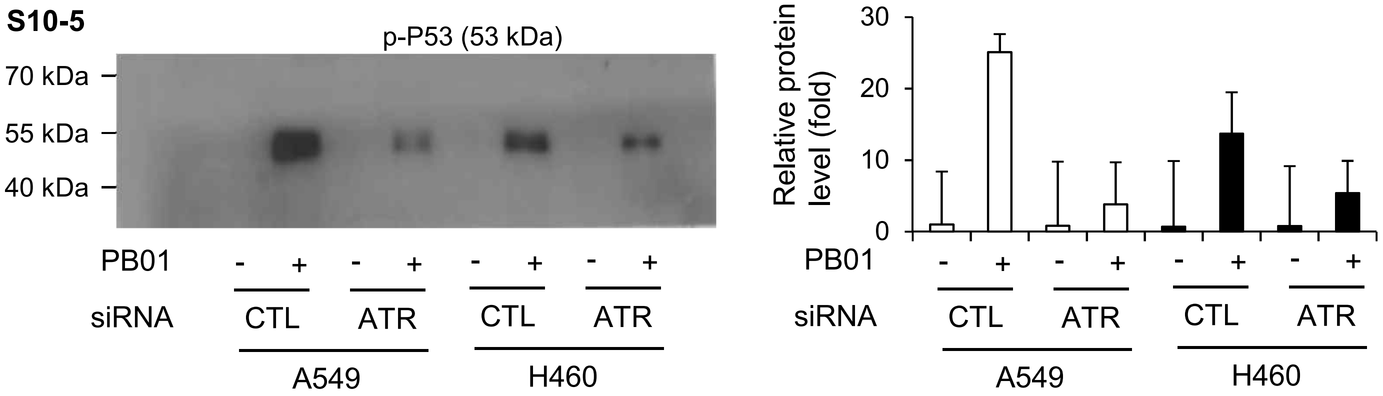

S10-6

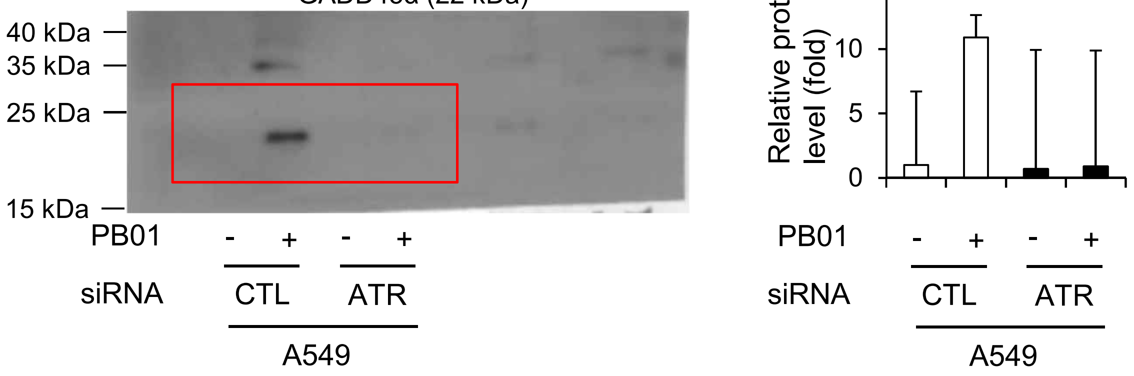

S10-7

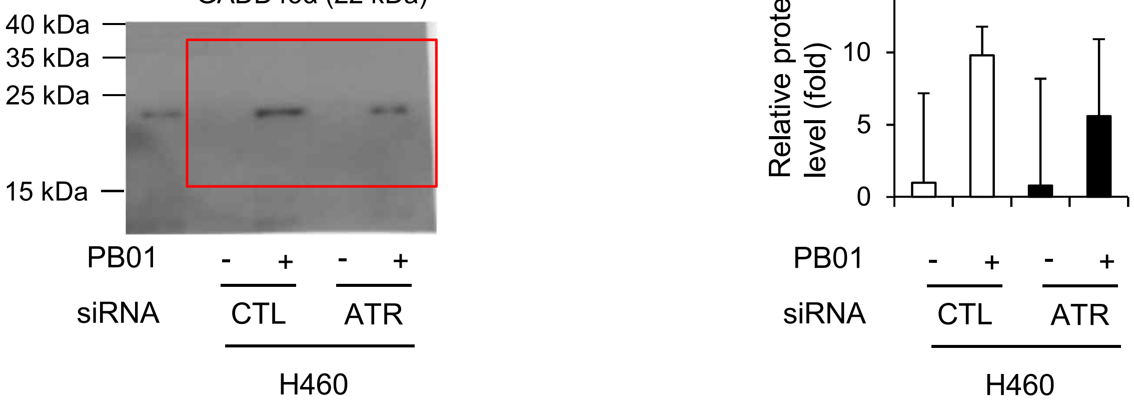

S10-8

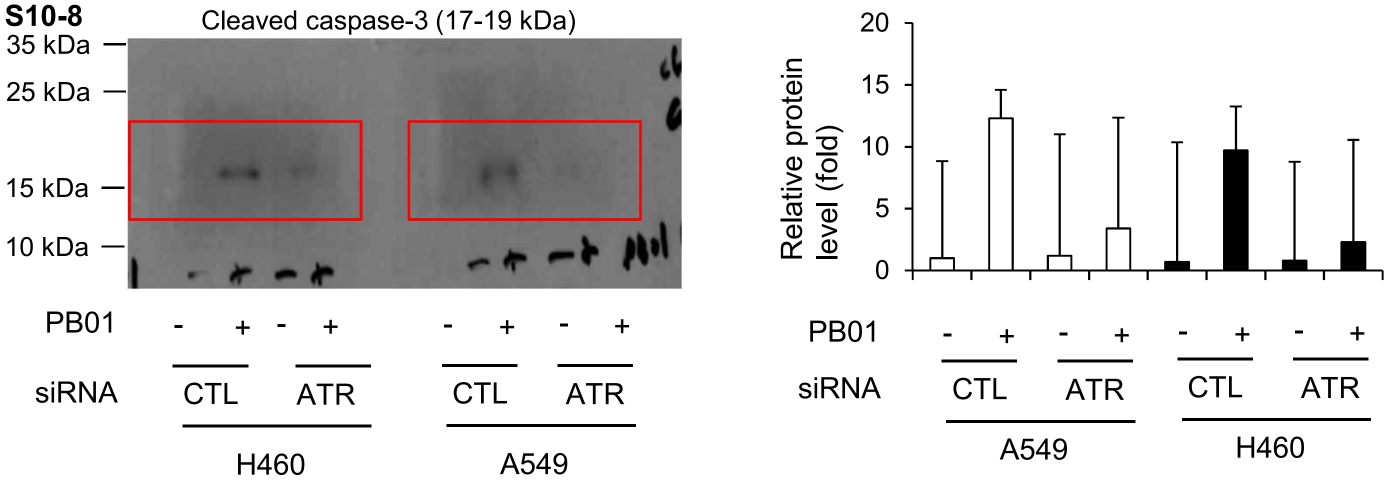

Figure S11 Supplementary data for Figure 3i

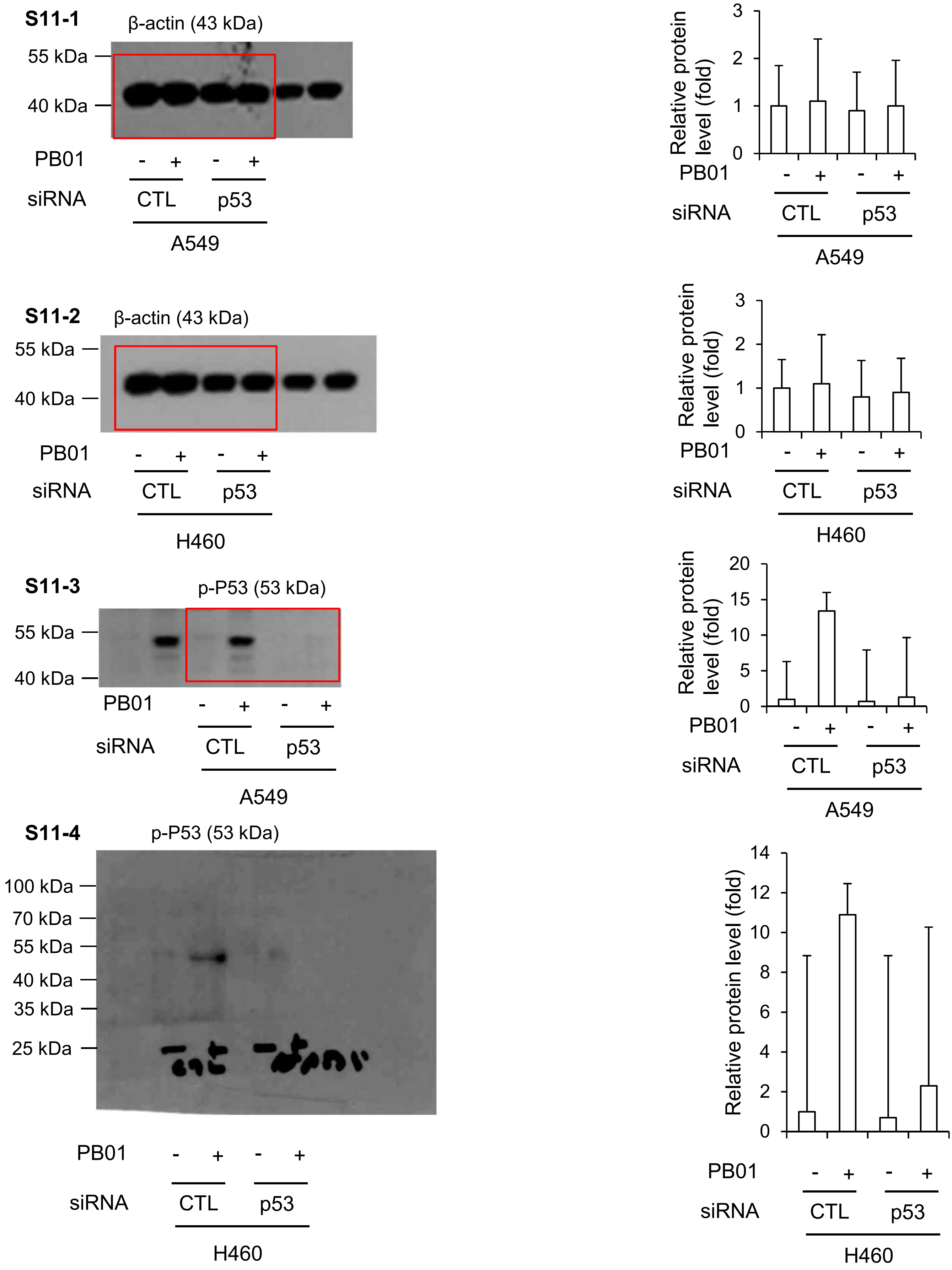

S11-5

GADD45α (22 kDa)

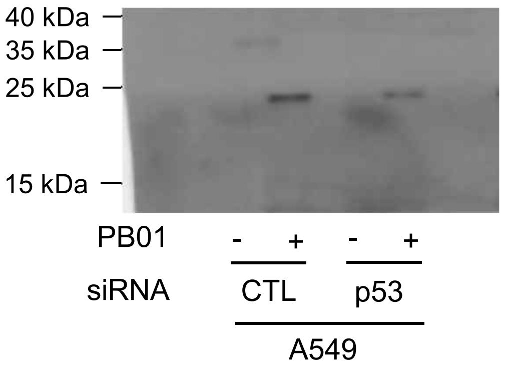

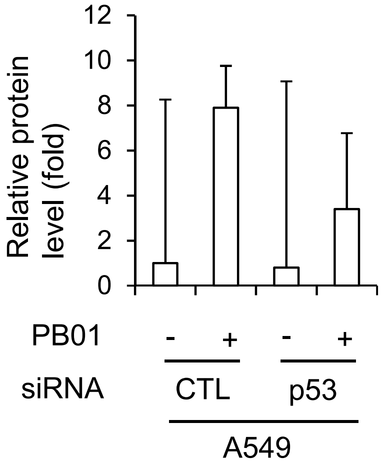

S11-6

GADD45α (22 kDa)

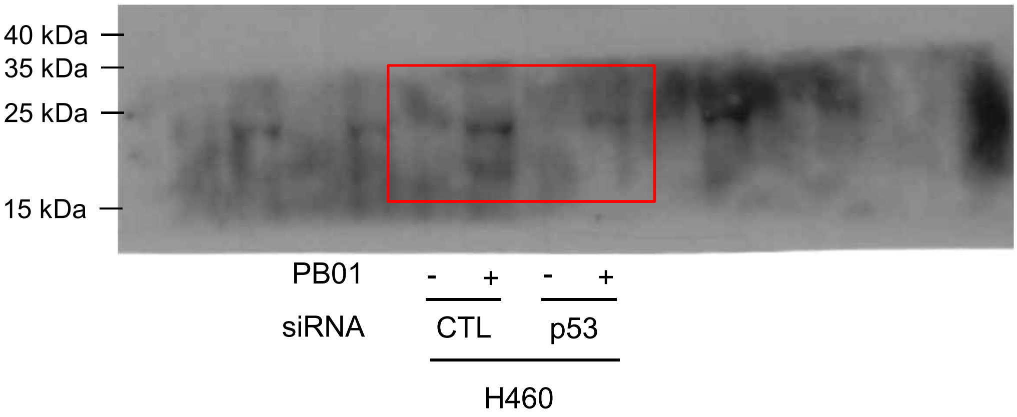

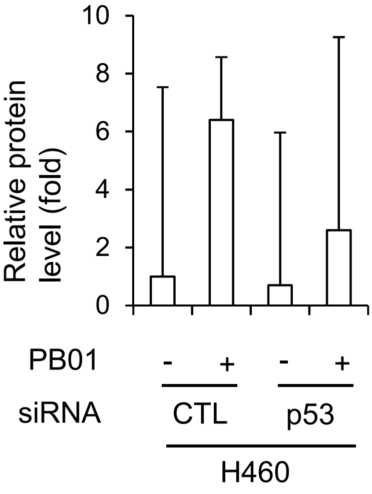

S11-7

Cleaved caspase-3 (17-19 kDa)

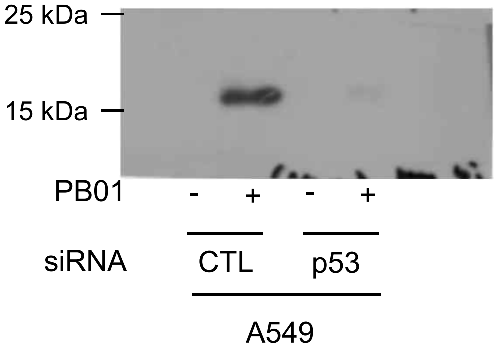

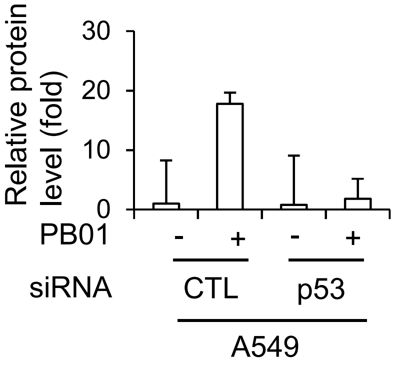

S11-8

Cleaved caspase-3 (17-19 kDa)

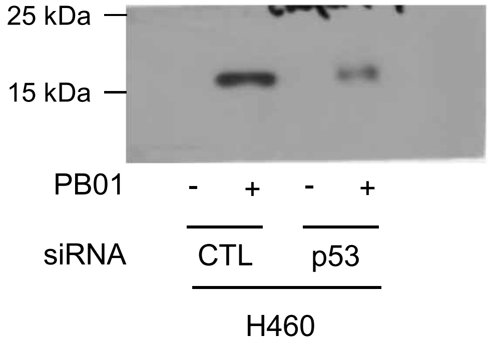

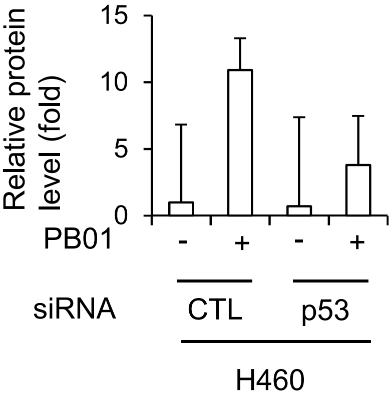

Figure S12. Supplementary materials for Figure 4a

S12-1

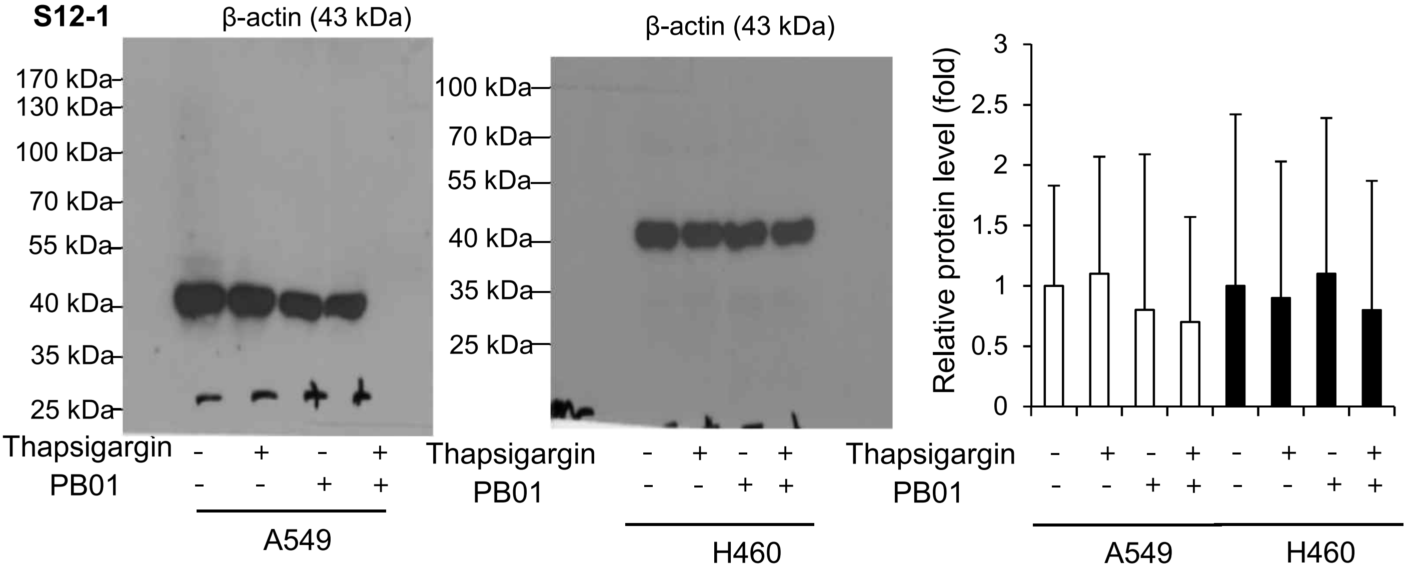

S12-2

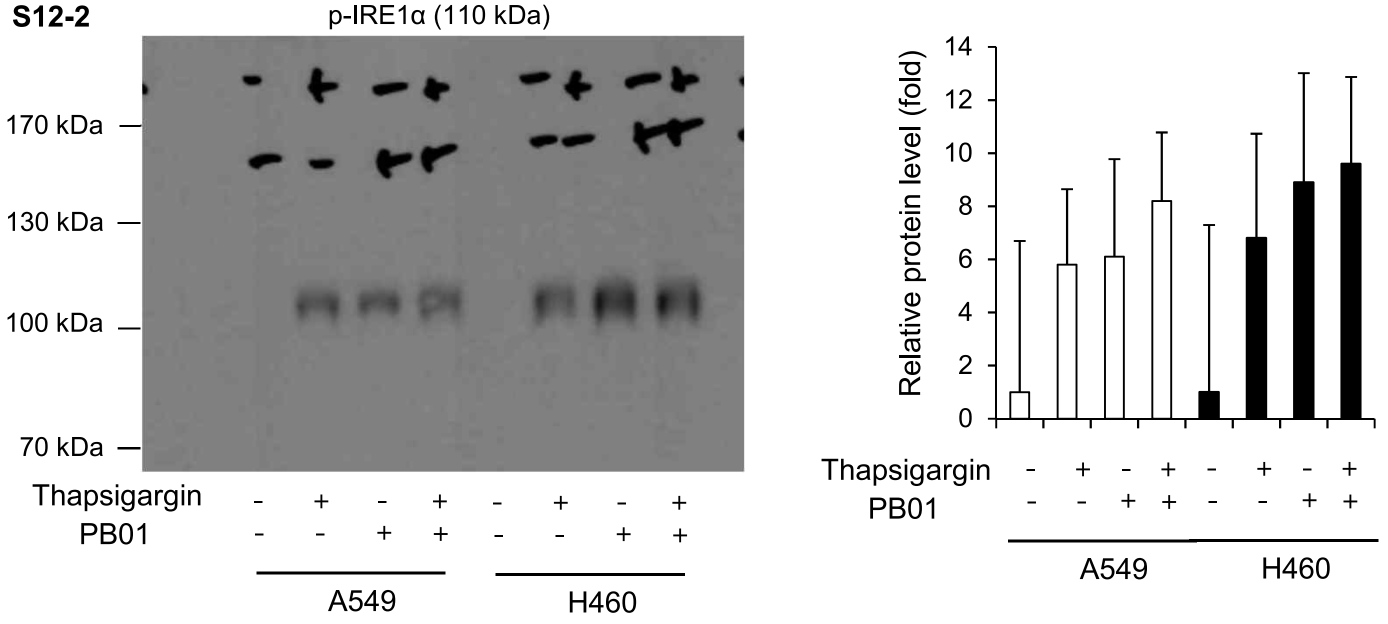

S12-3

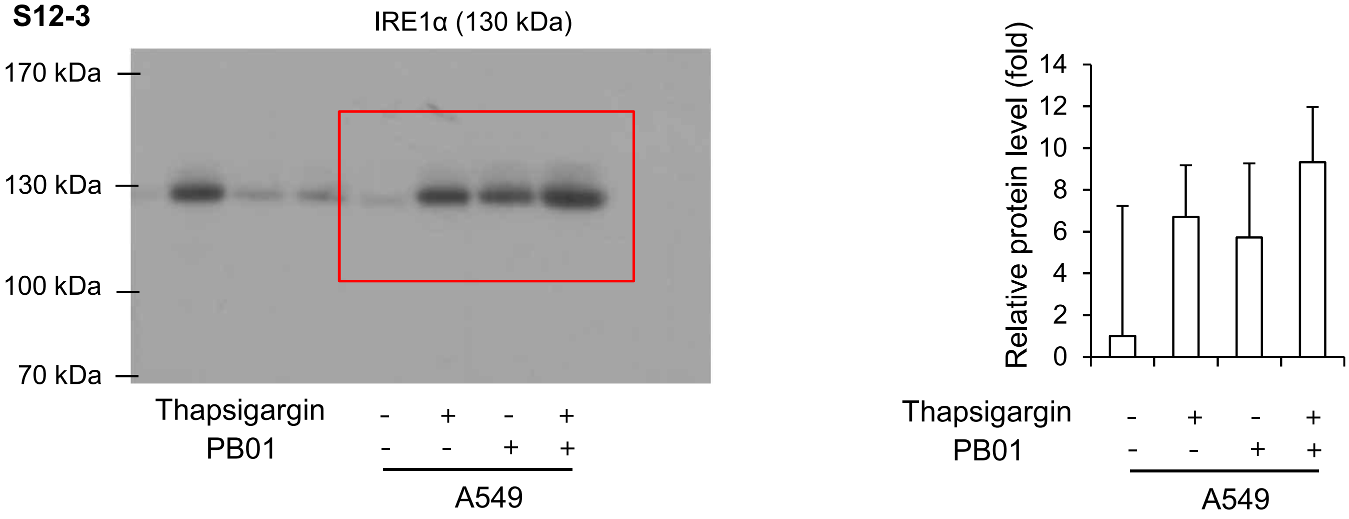

S12-4

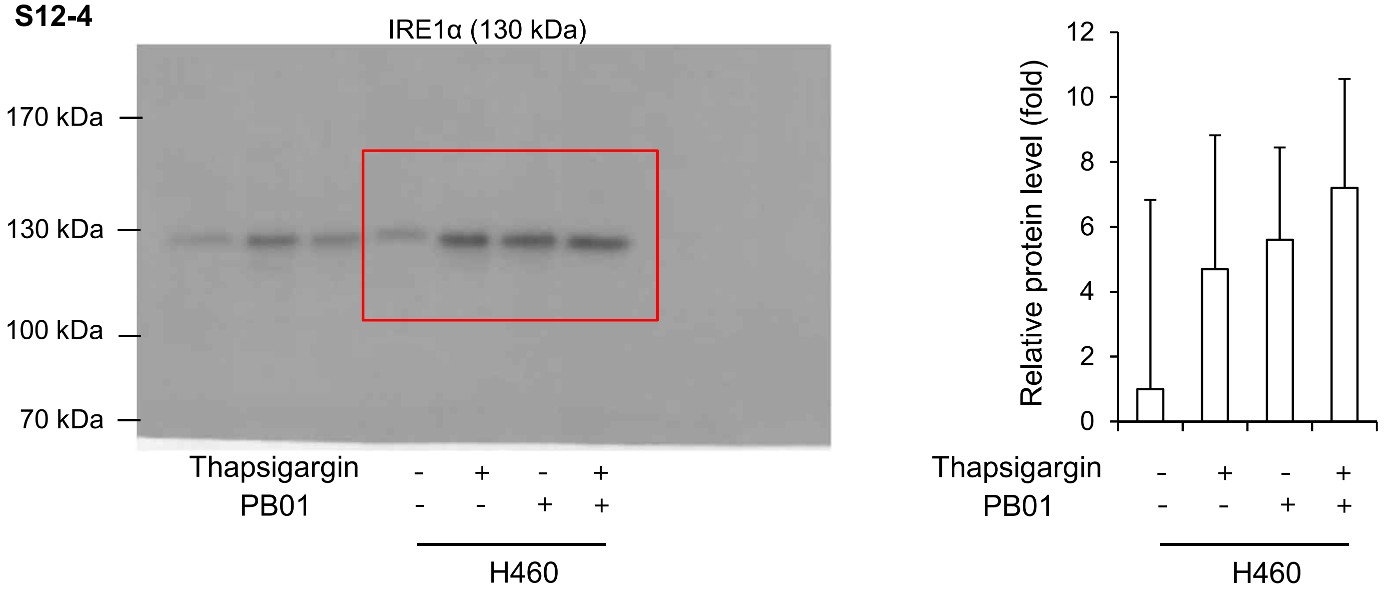

S12-5

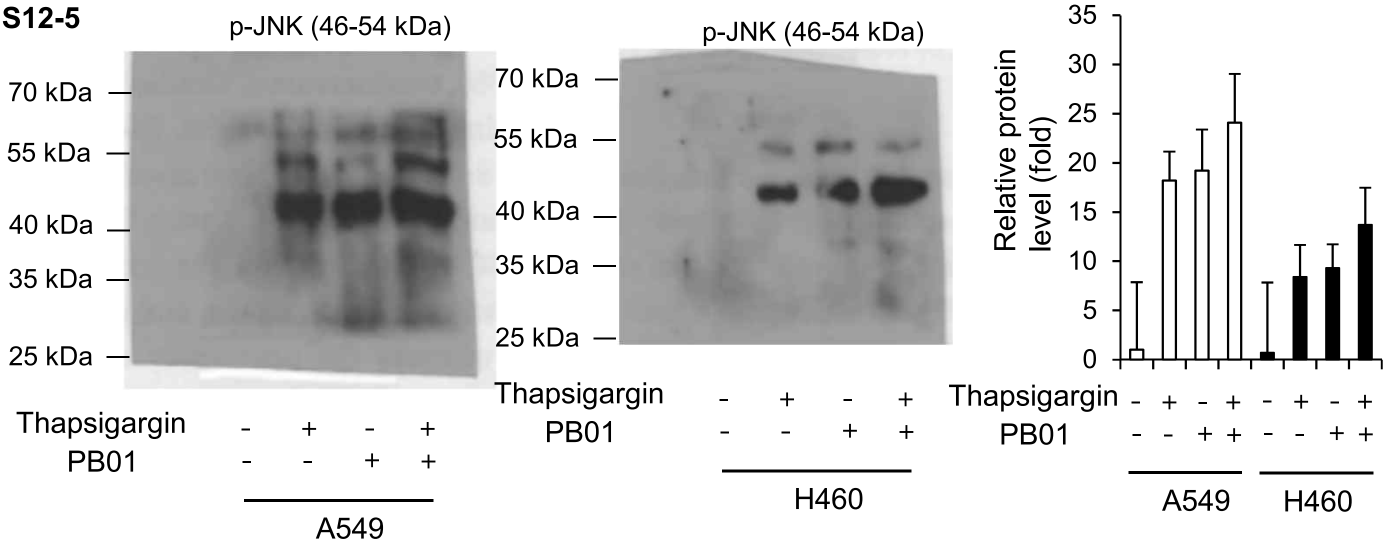

S12-6

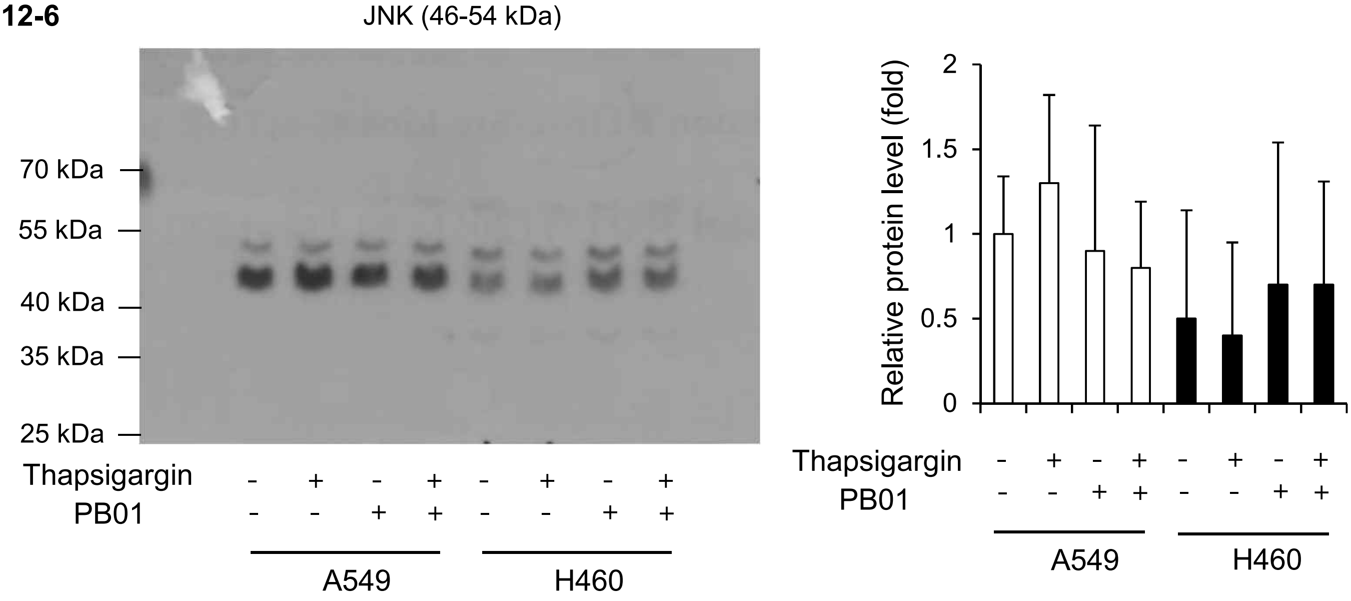

S12-7

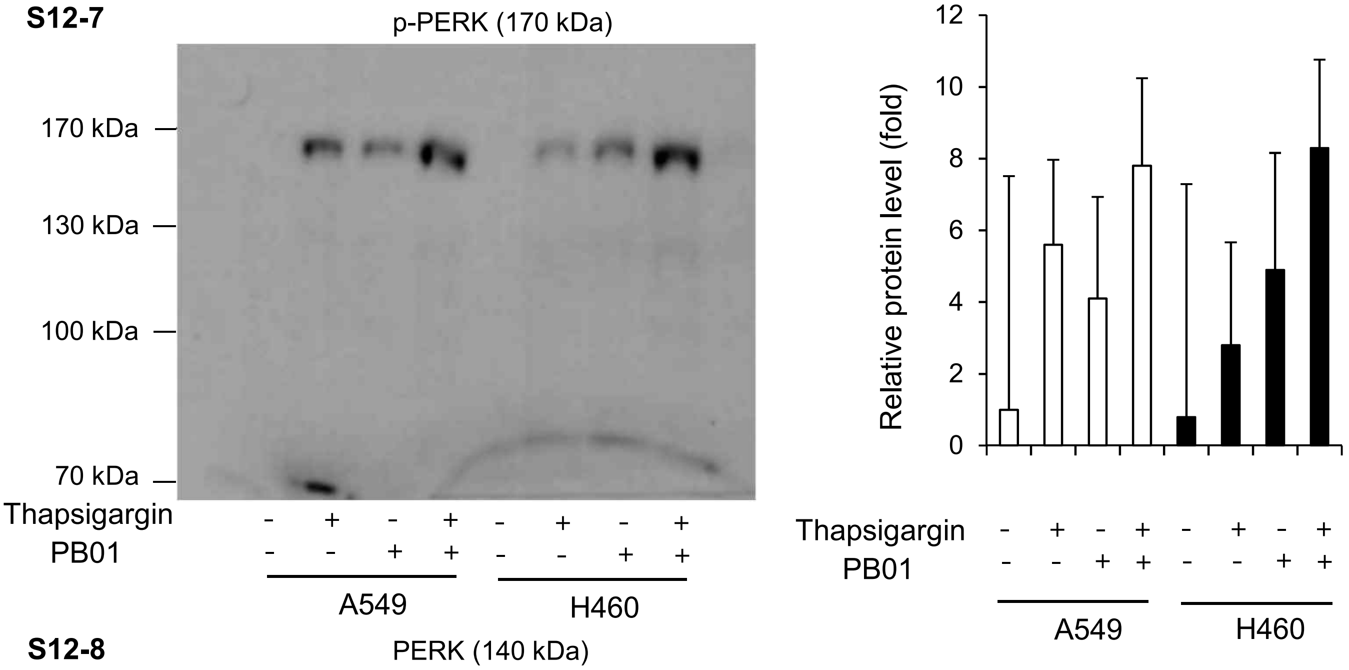

S12-8

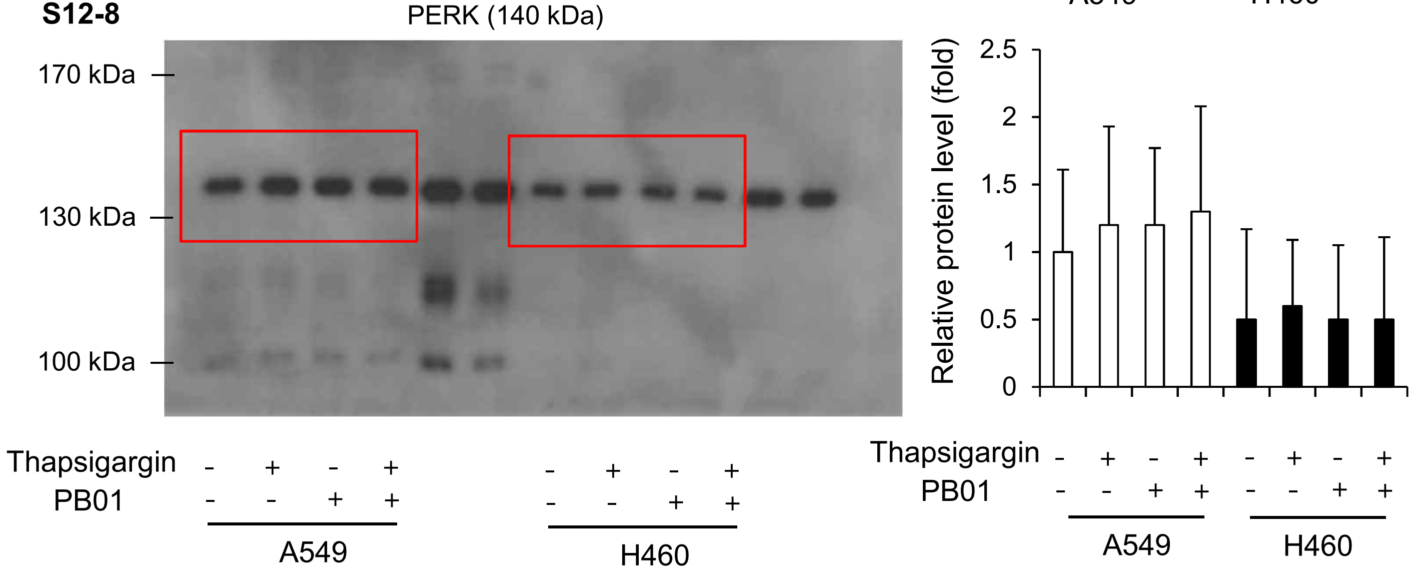

S12-9

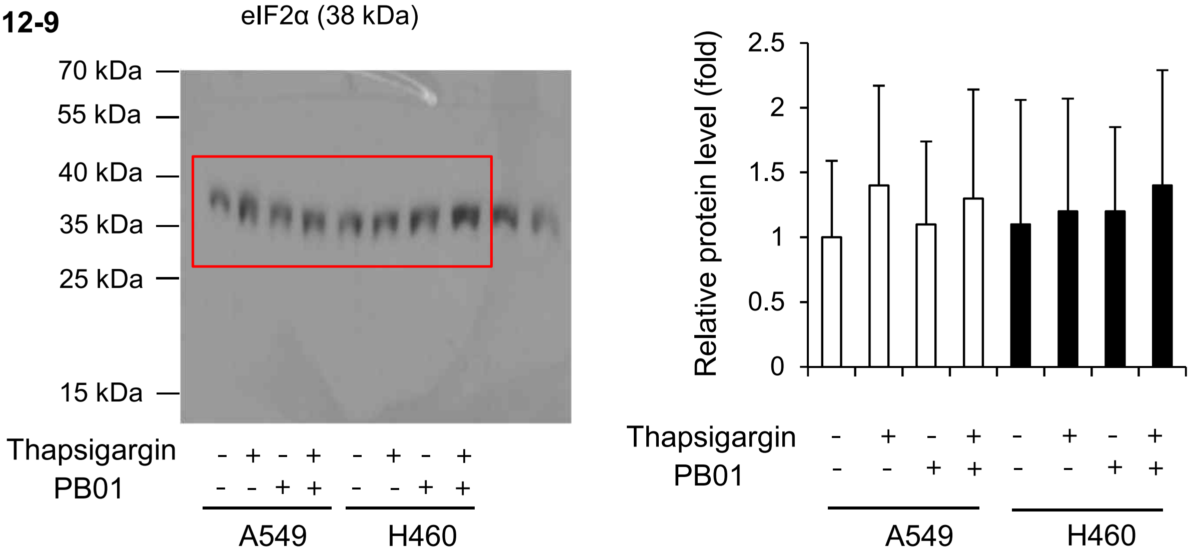

S12-10

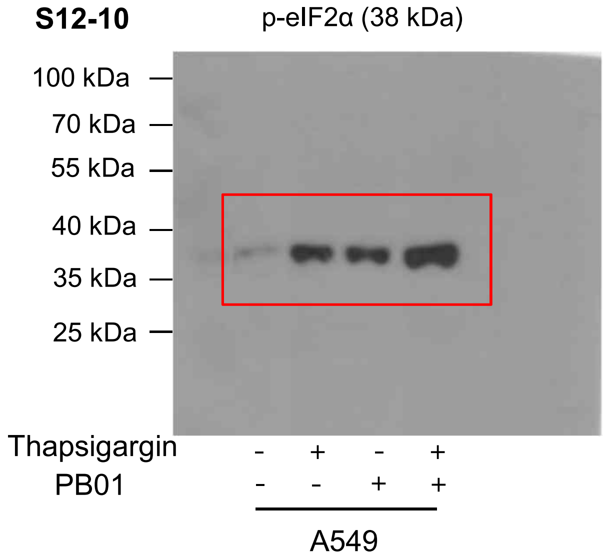

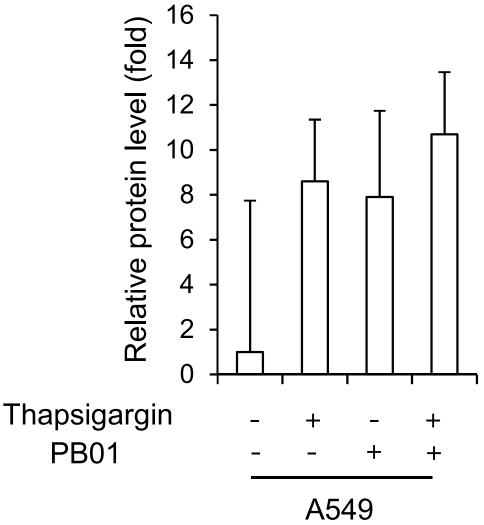

S12-11

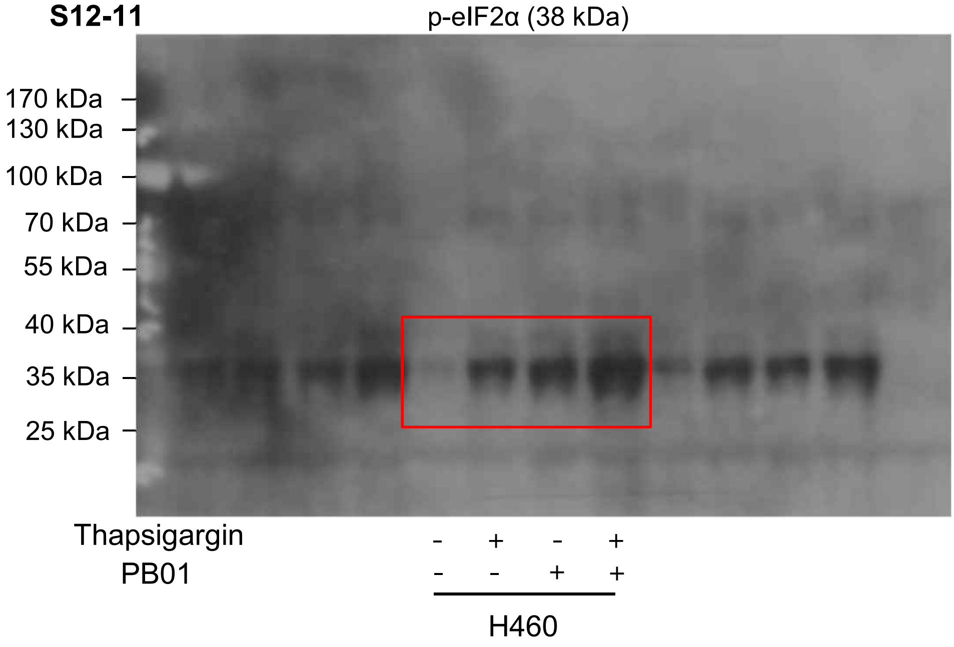

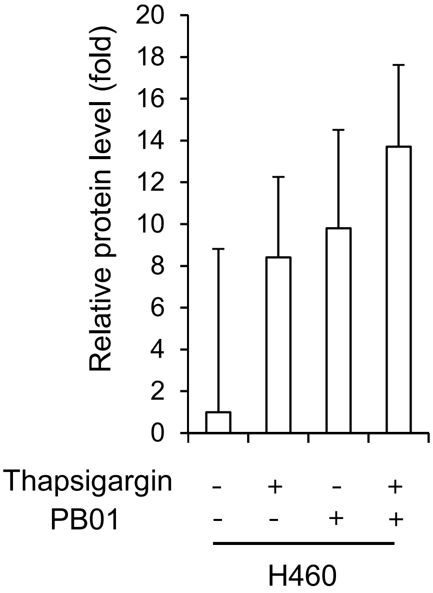

S12-12

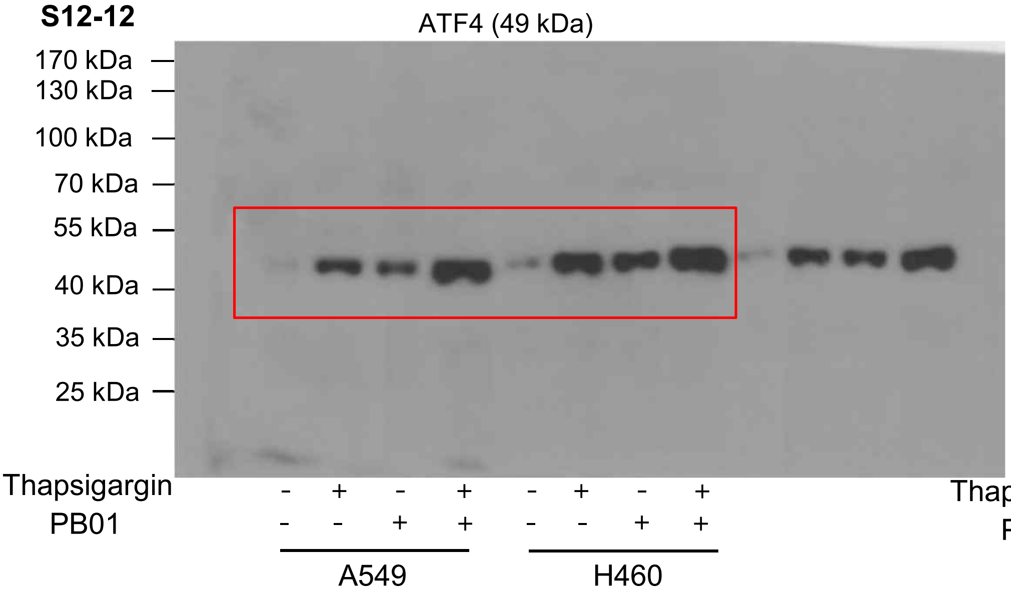

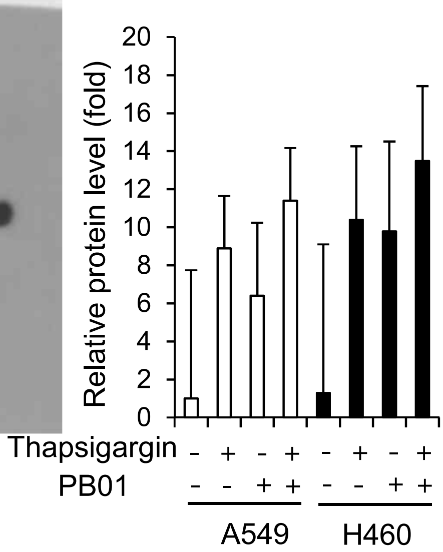

S12-13 CHOP (27 kDa)

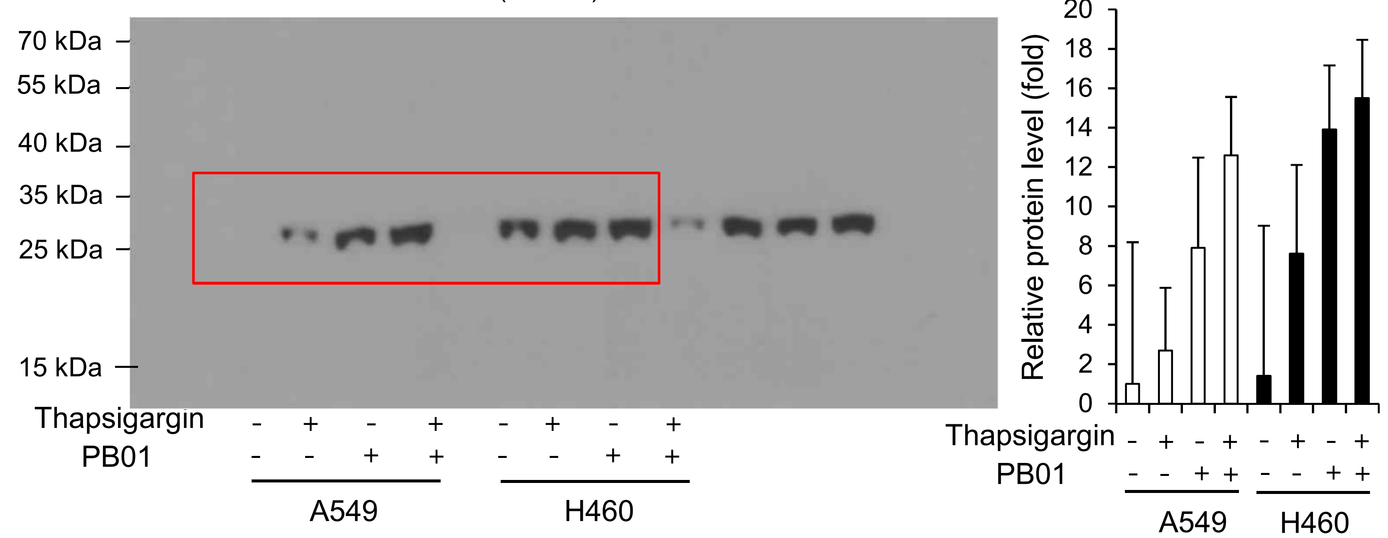

S12-14 GADD45α (22 kDa)

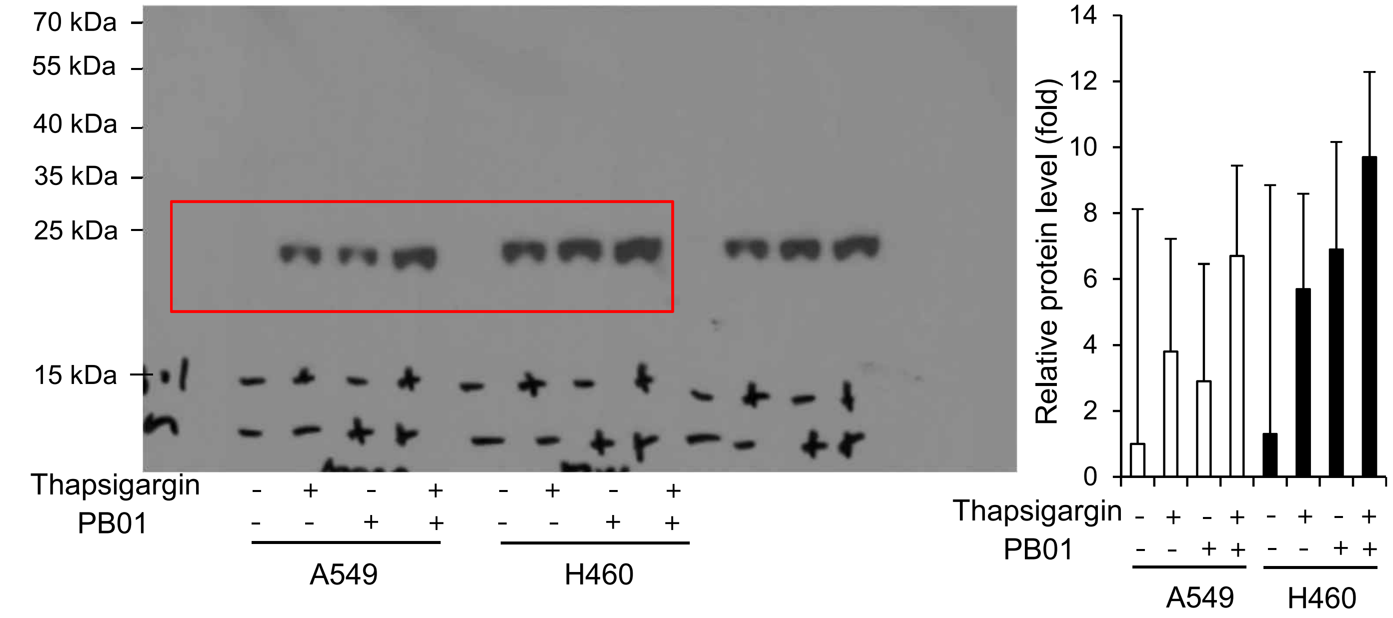

S12-15 Cleaved caspase-3 (17-19 kDa)

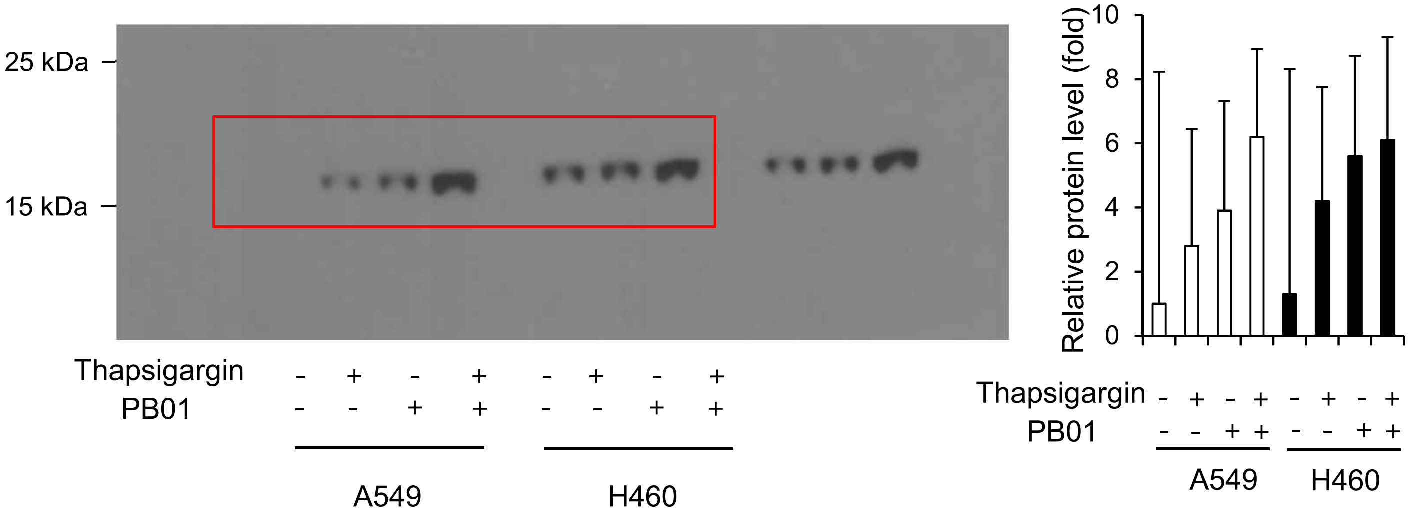

Figure S13. Supplementary materials for Figure 4b

S13-1

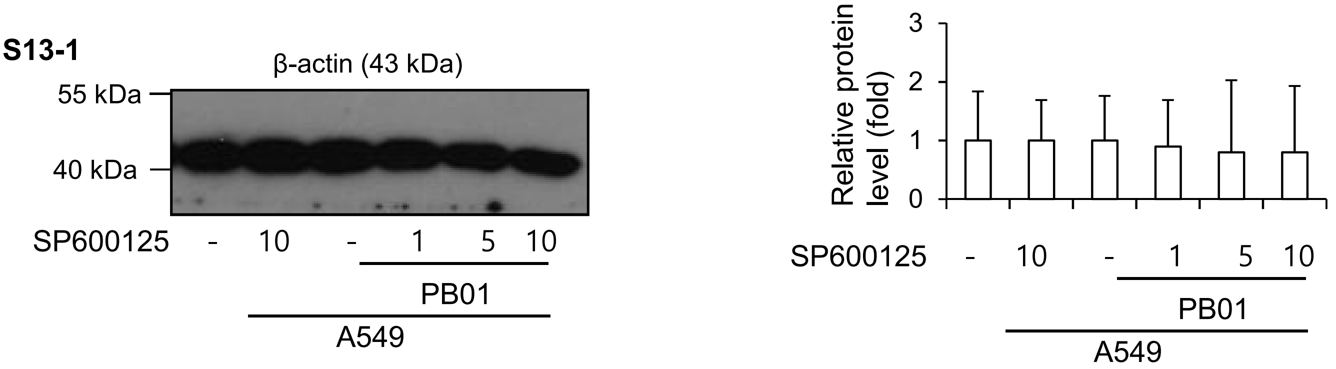

S13-2

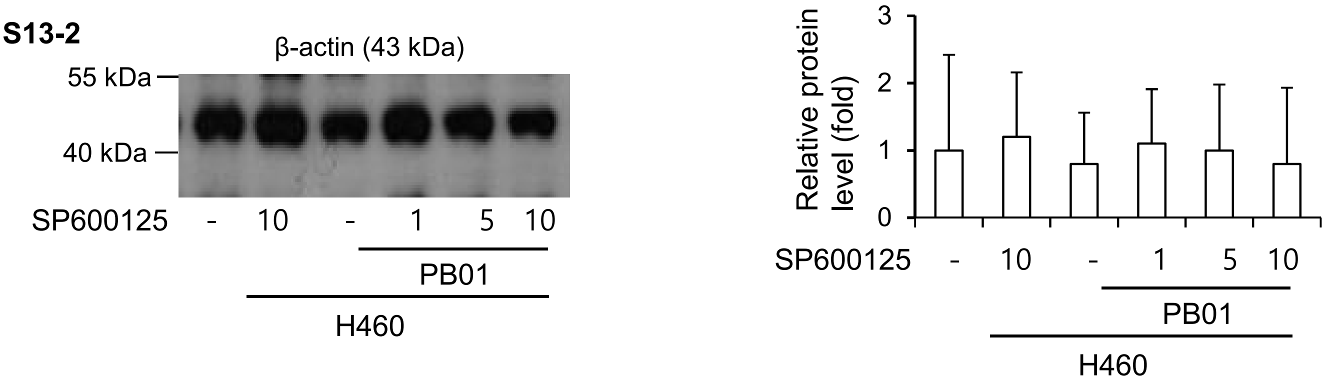

S13-3

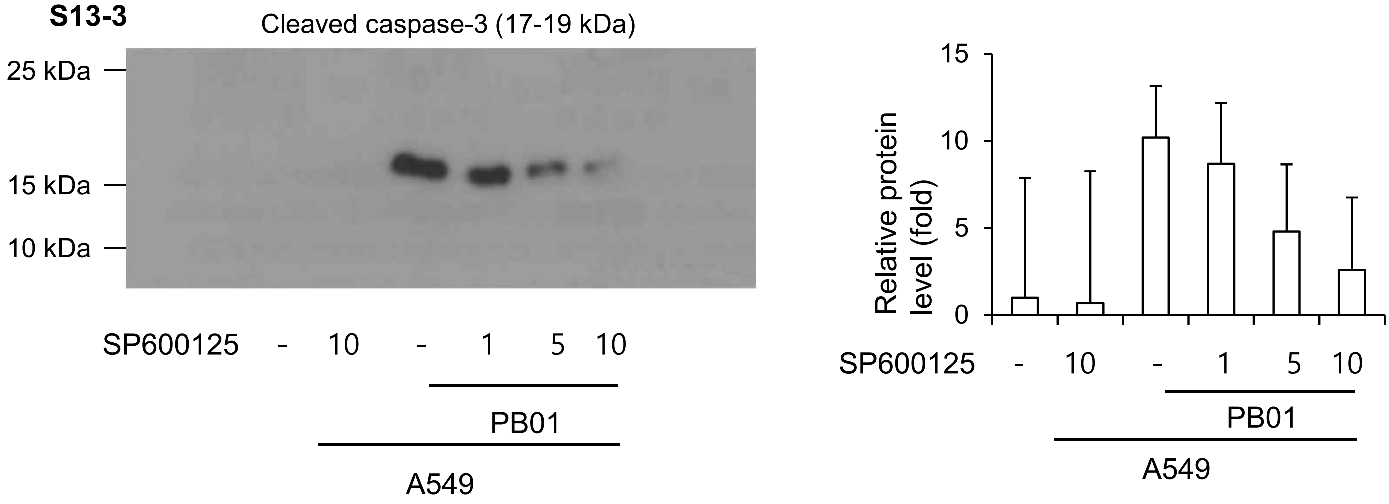

S13-4

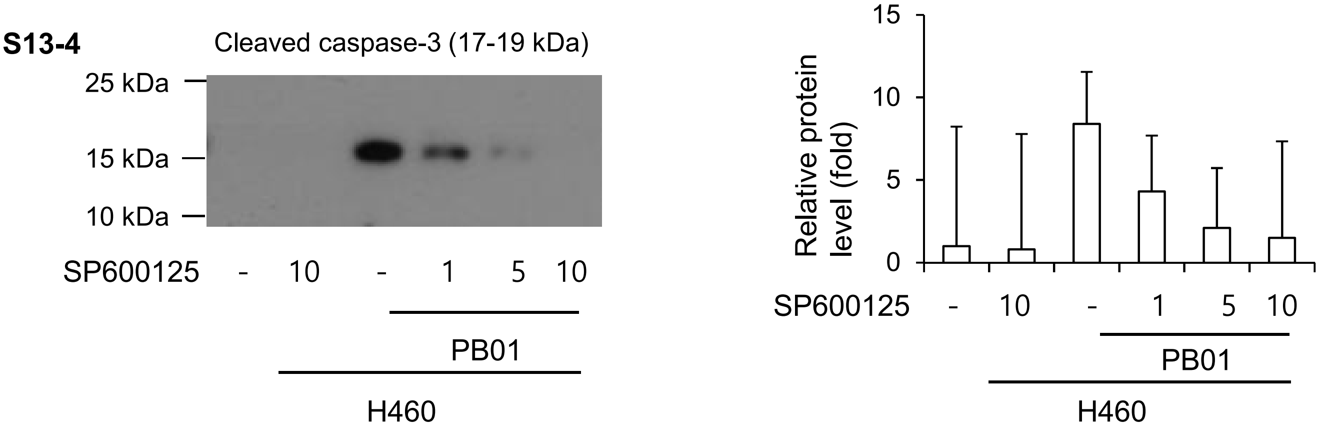

S13-5

Cleaved caspase-9 (37 kDa)

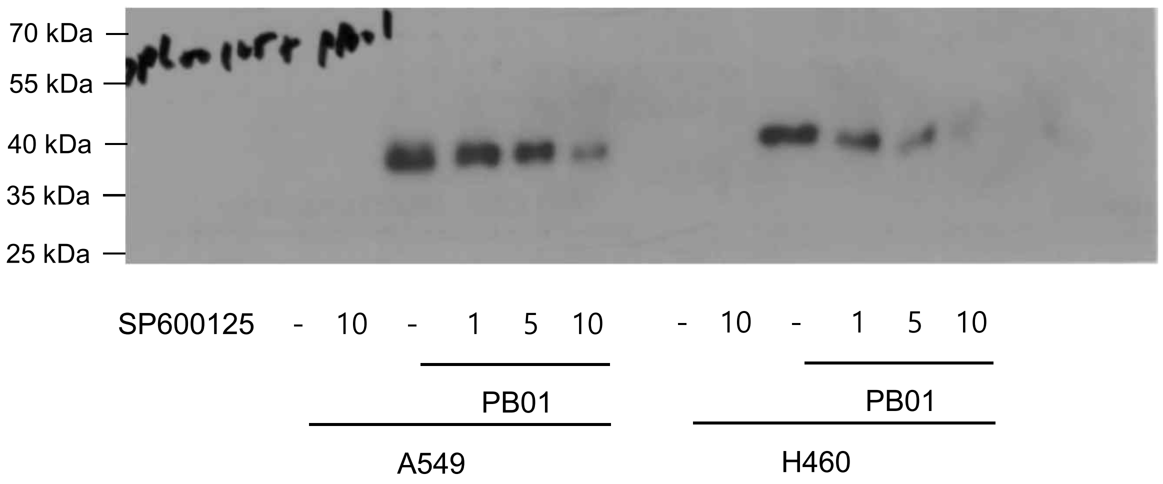

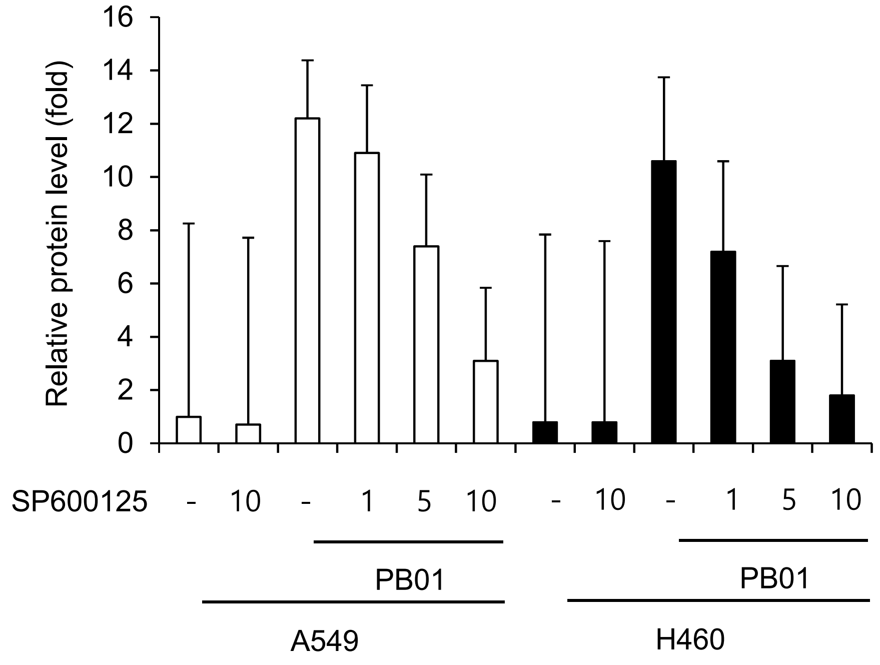

Figure S14. supplementary data for Figure 4d

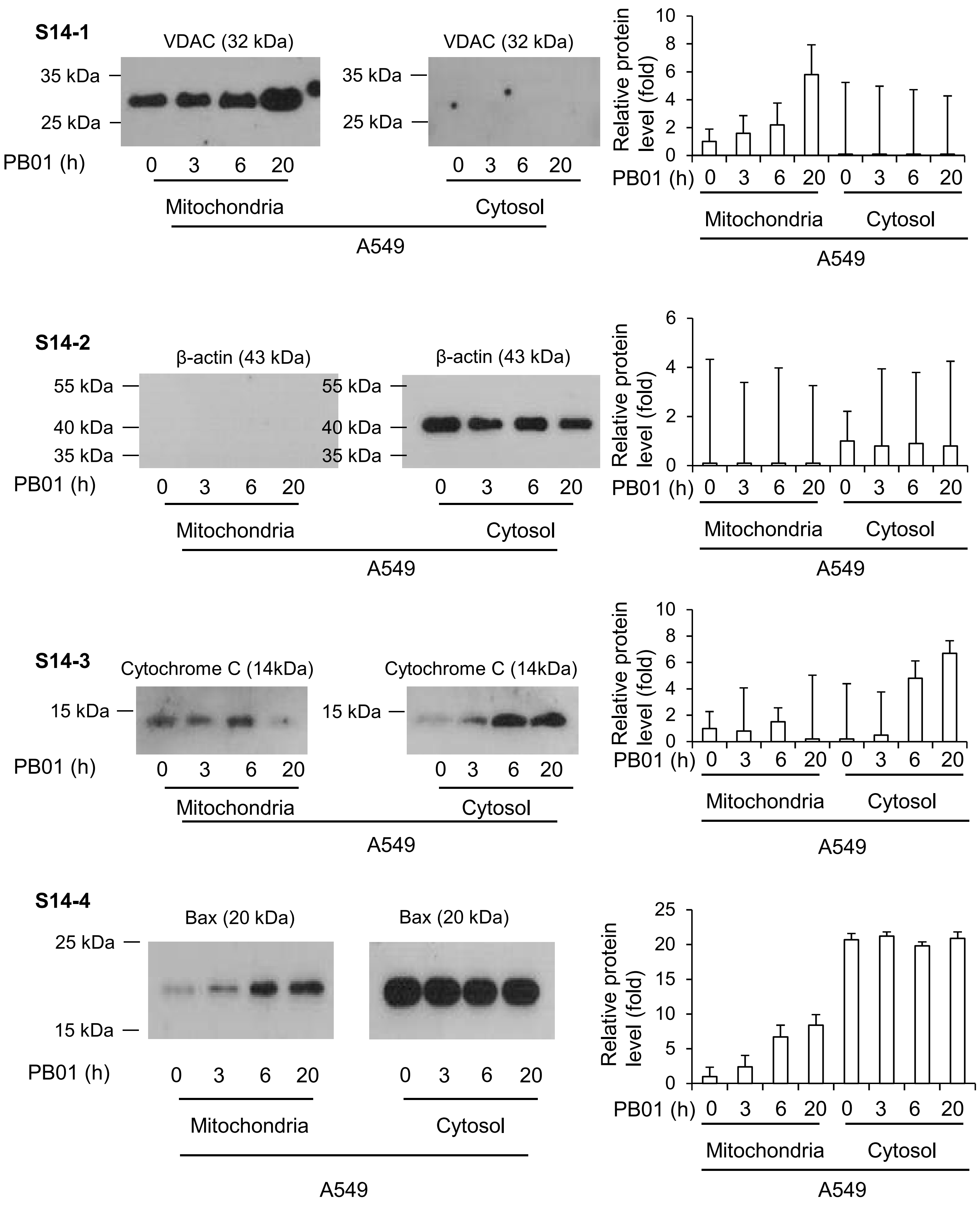

S14-5

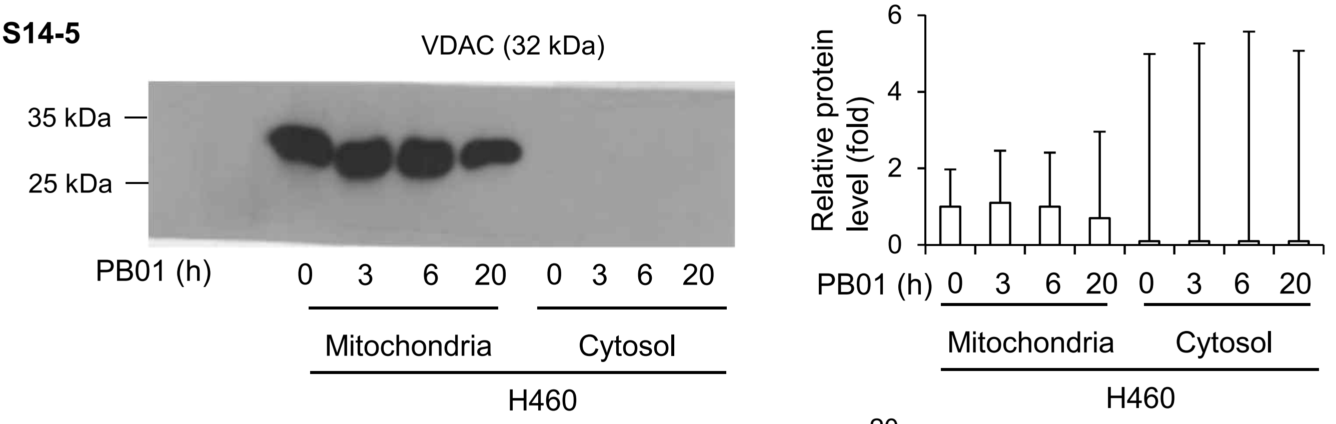

S14-6

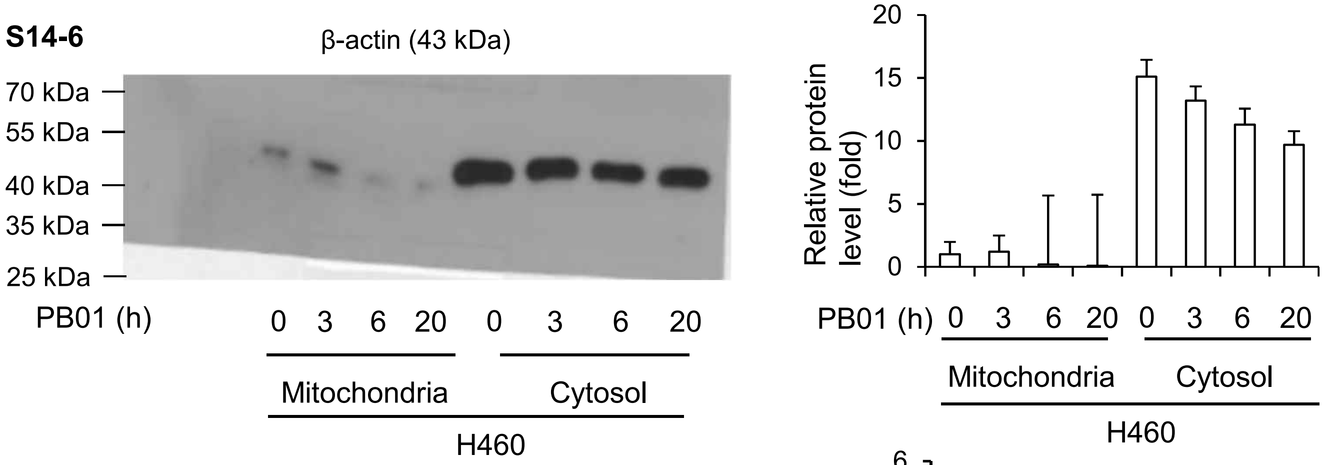

S14-7

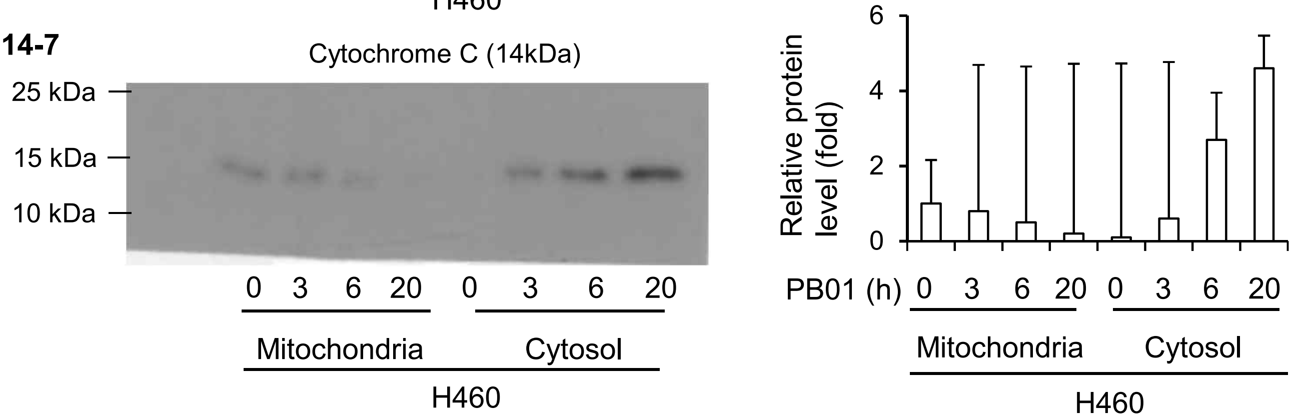

S14-8

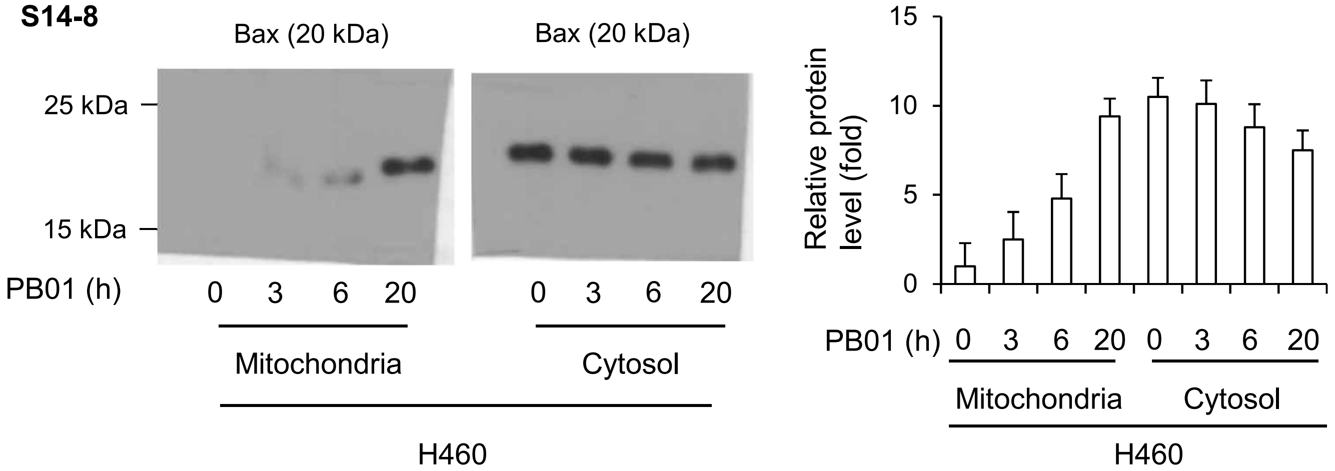

Figure S15. Supplementary data for Figure 4g

S15-1

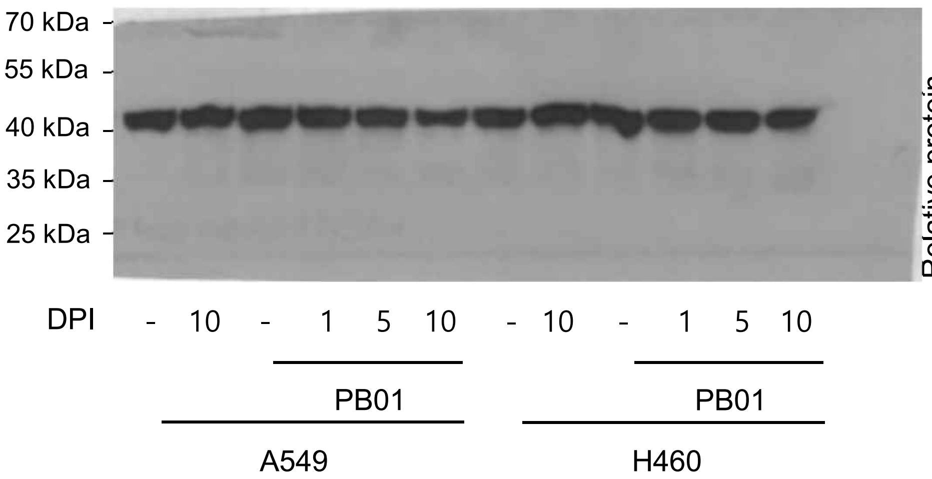

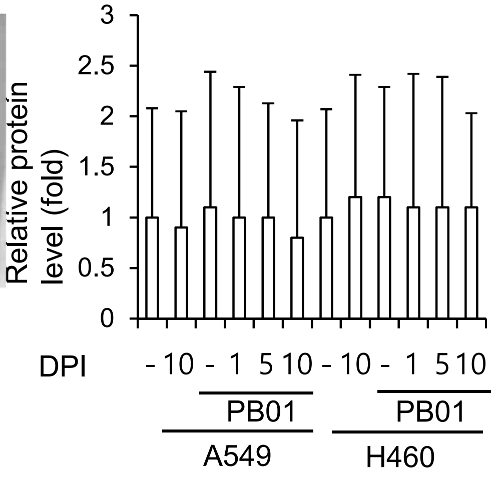

S15-2

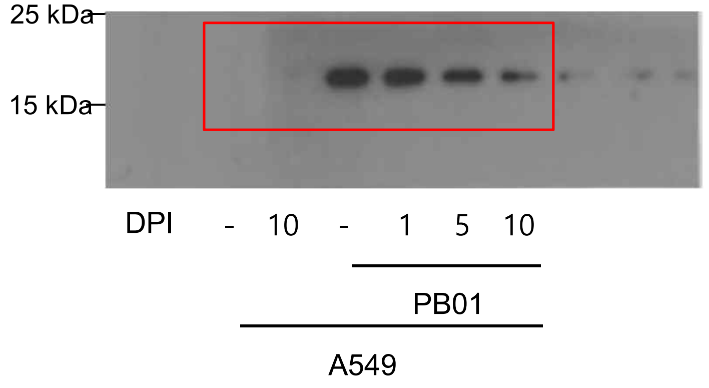

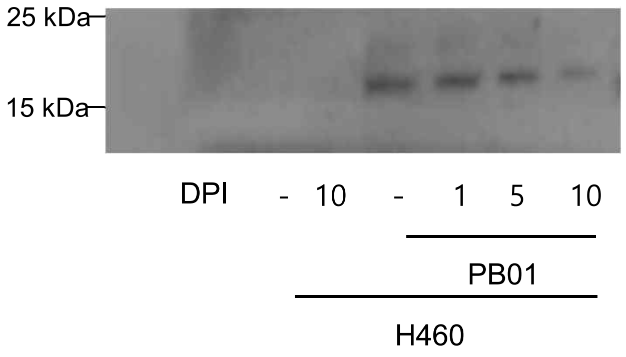

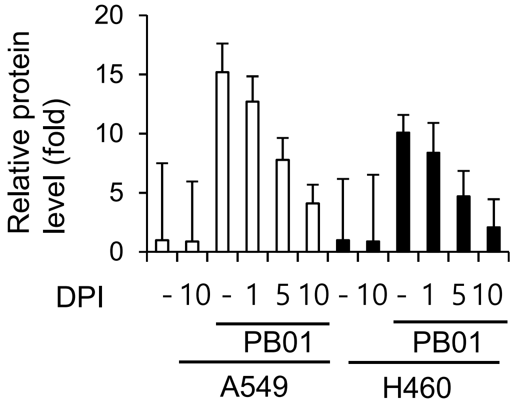

S15-3

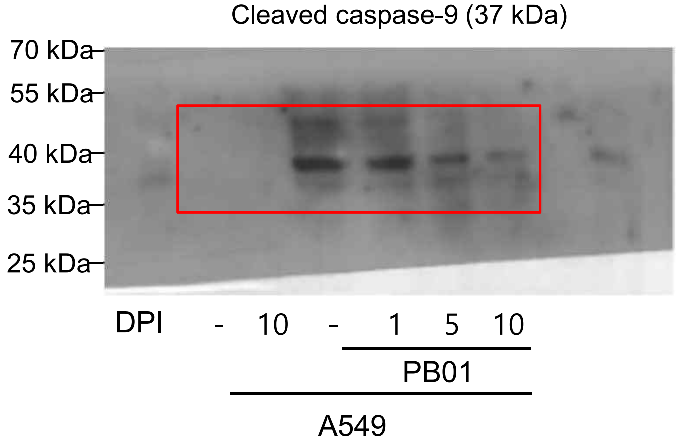

Cleaved caspase-9 (37 kDa)
70 kDa
55 kDa
40 kDa
35 kDa
25 kDa
DPI
- 10 - 1 5 10
PB01
A549

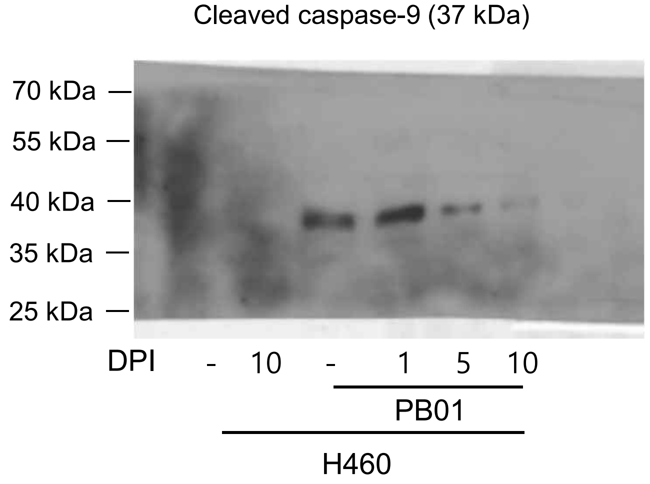

Cleaved caspase-9 (37 kDa)
70 kDa
55 kDa
40 kDa
35 kDa
25 kDa
DPI
- 10 - 1 5 10
PB01
H460

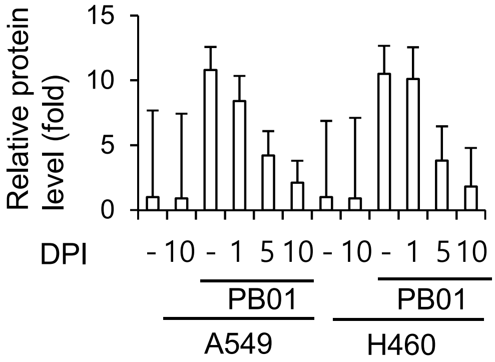

Relative protein level (fold)
15
10
5
0
DPI
- 10 - 1 5 10 - 10 - 1 5 10
PB01
PB01
A549
H460

Figure S16. Supplementary materials for Figure S2a

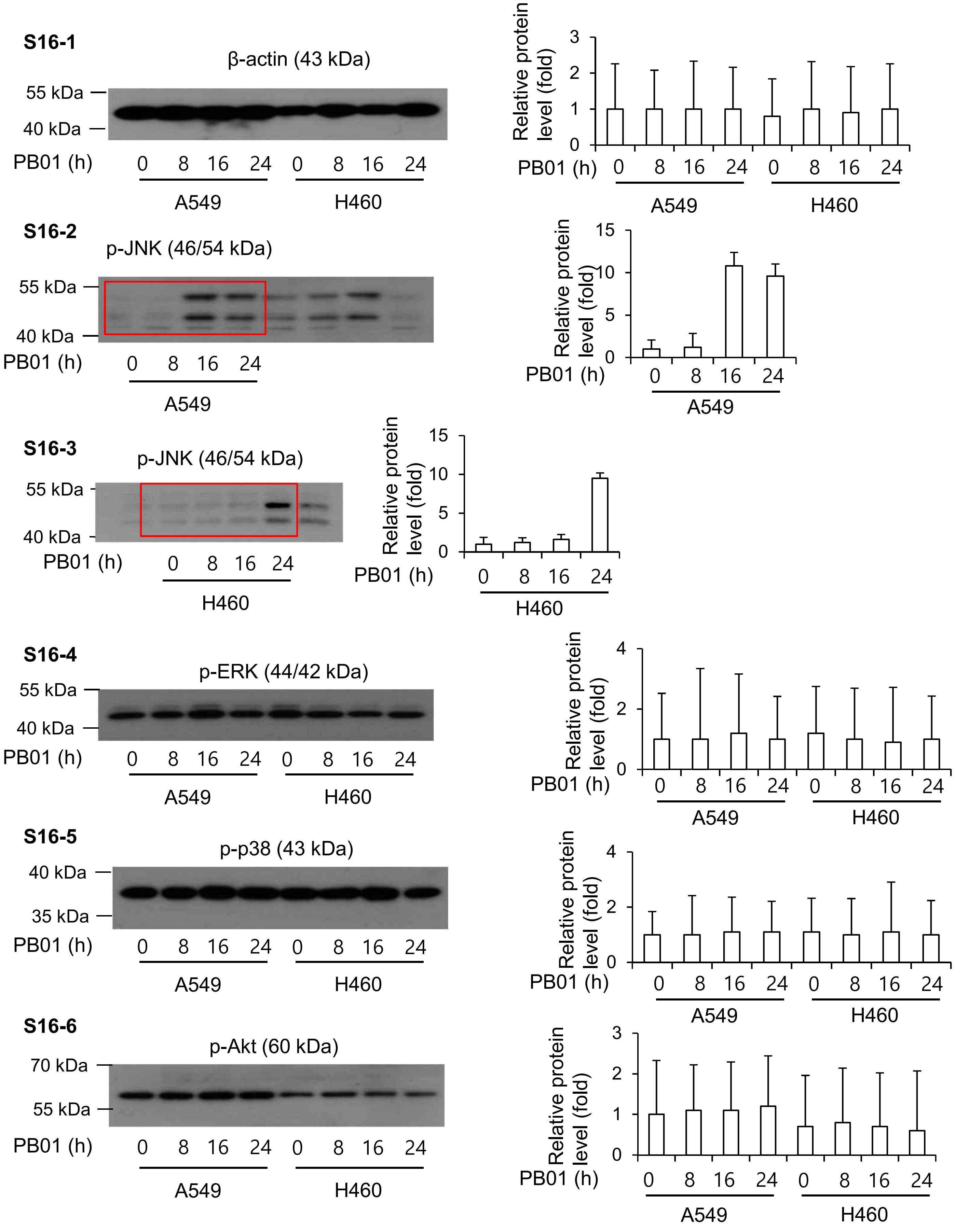

Figure S17. Supplementary materials for Figure S1d

S17-1

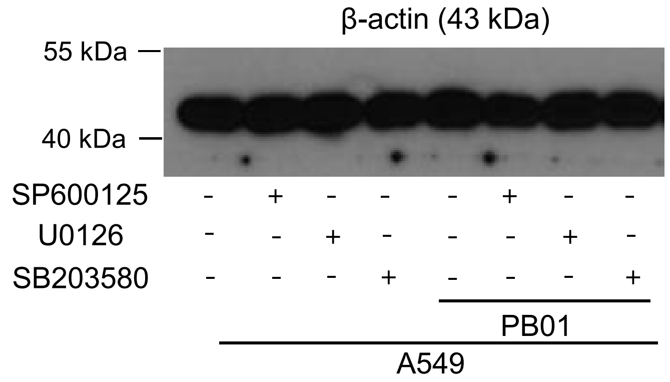

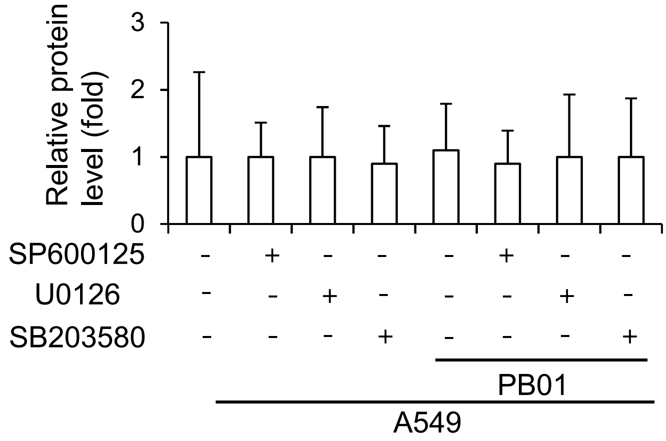

S17-2

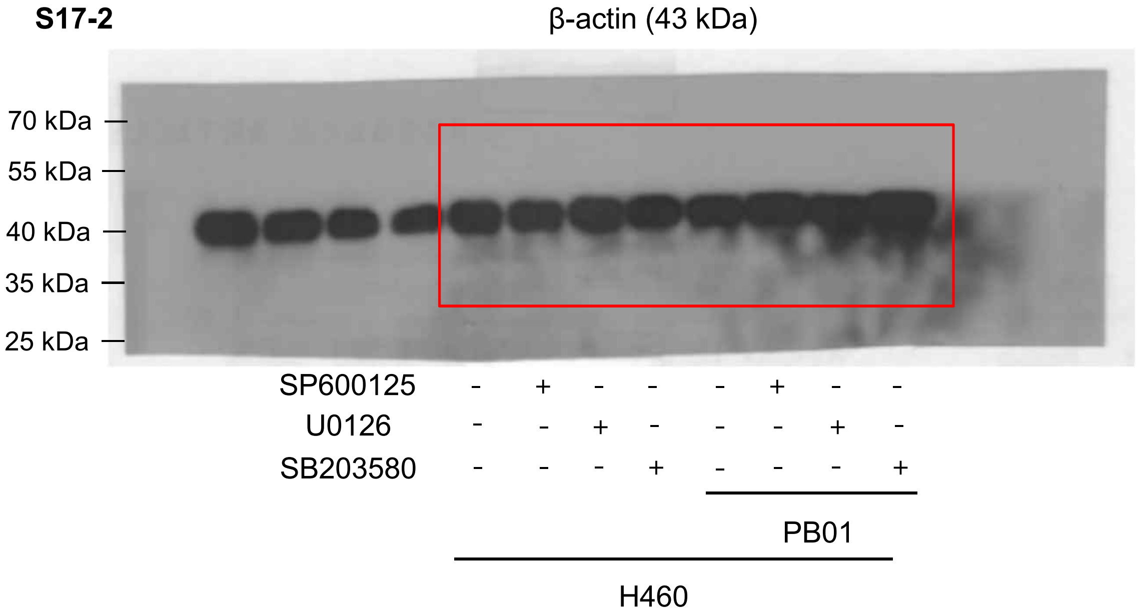

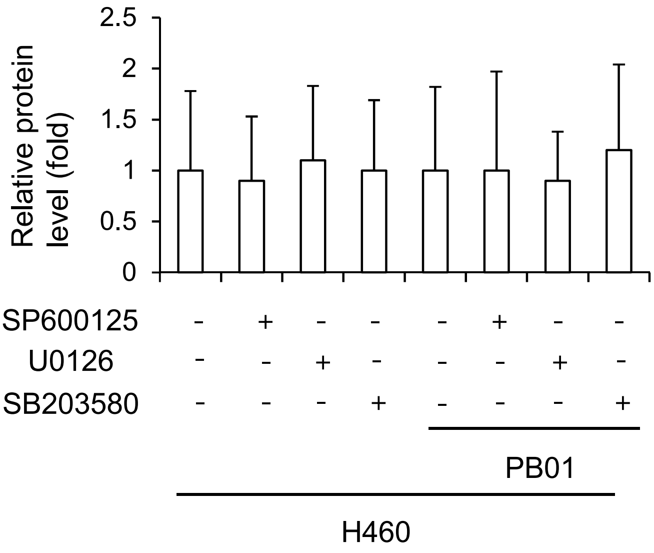

S17-3

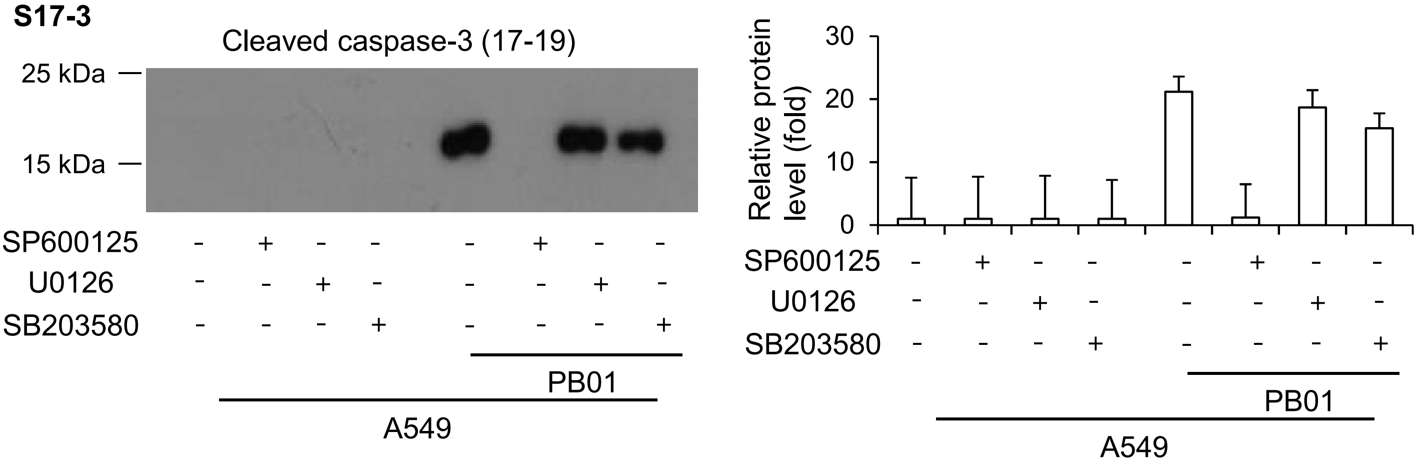
Cleaved caspase-3 (17-19)
25 kDa
15 kDa
SP600125
U0126
SB203580
PB01
A549
Relative protein level (fold)
30
20
10
0

S17-4

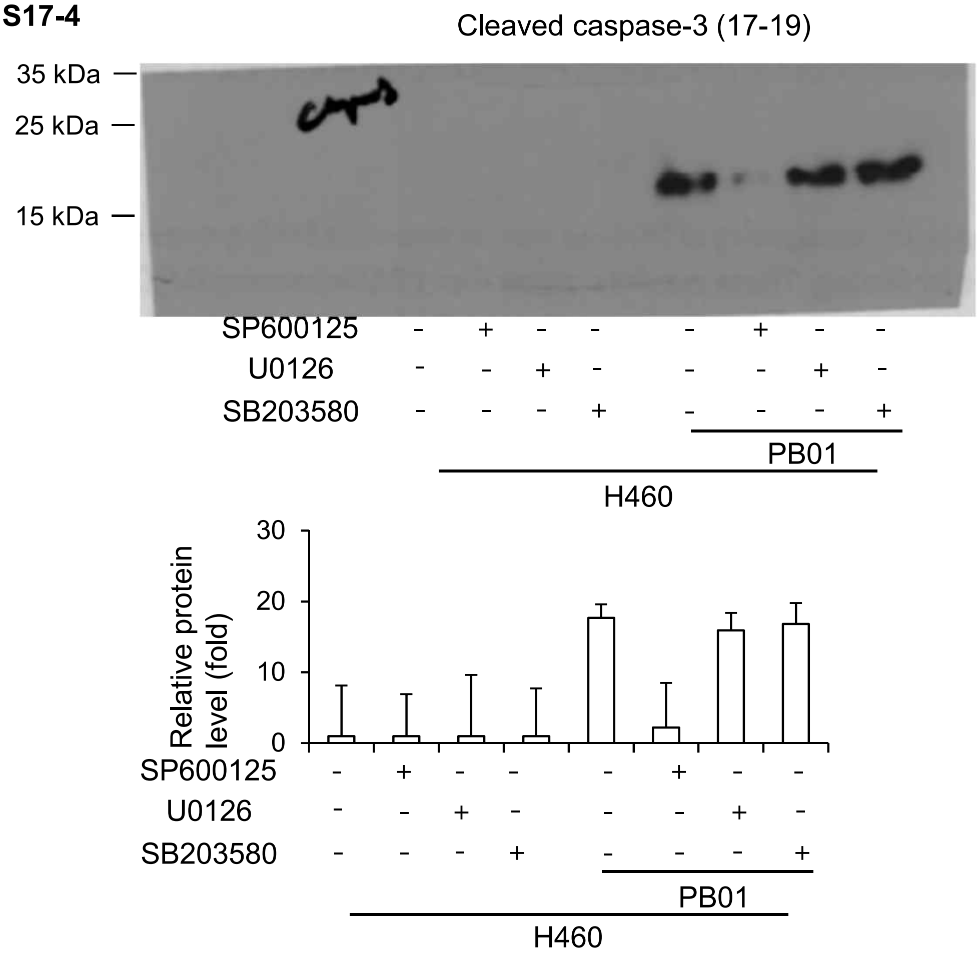
Cleaved caspase-3 (17-19)
35 kDa
25 kDa
15 kDa
SP600125
U0126
SB203580
PB01
H460
Relative protein level (fold)
30
20
10
0

Figure S18. Supplementary materials for Figure 5b

S18-1

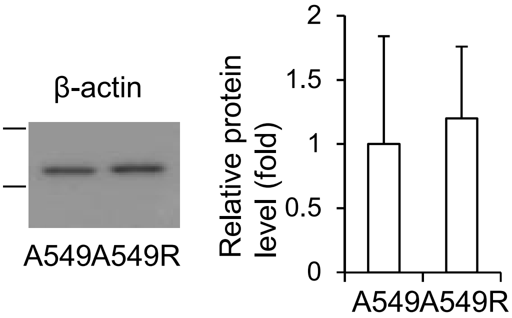

S15-2

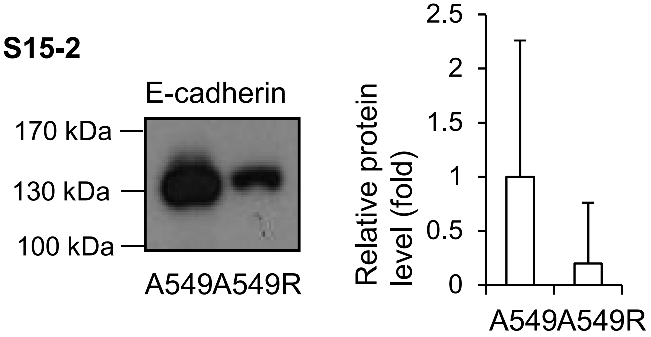

S18-3

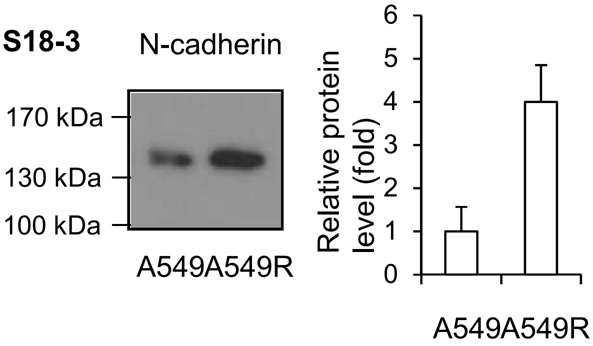

S15-4

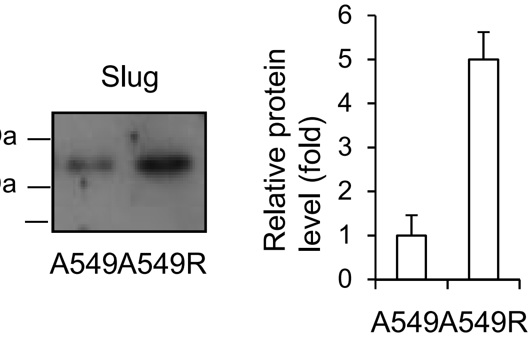

S18-5

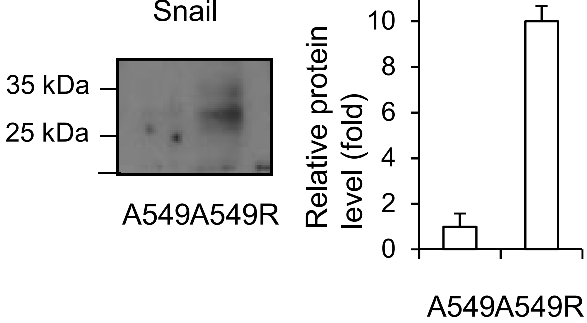

Figure S19. Supplementary materials for Figure 6c

S19-1

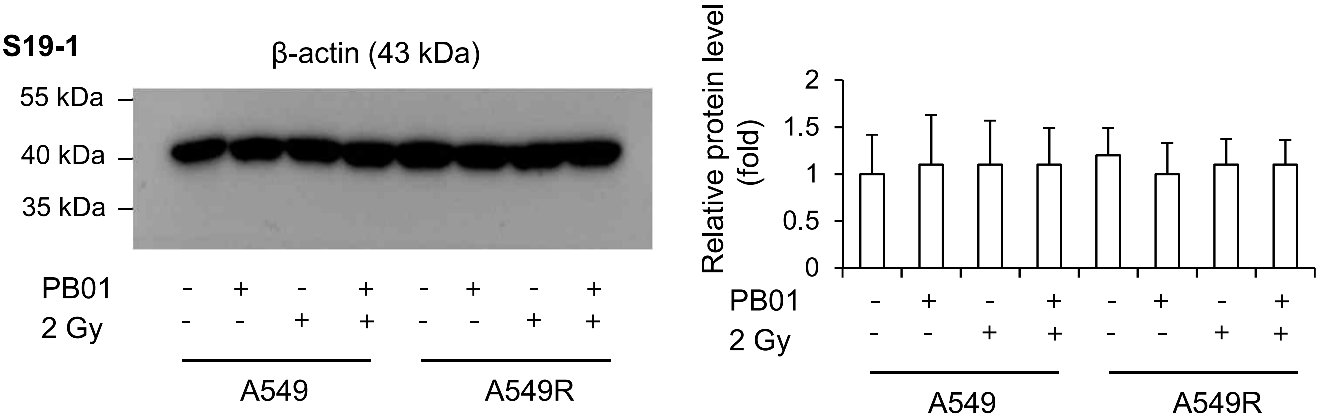

S19-2

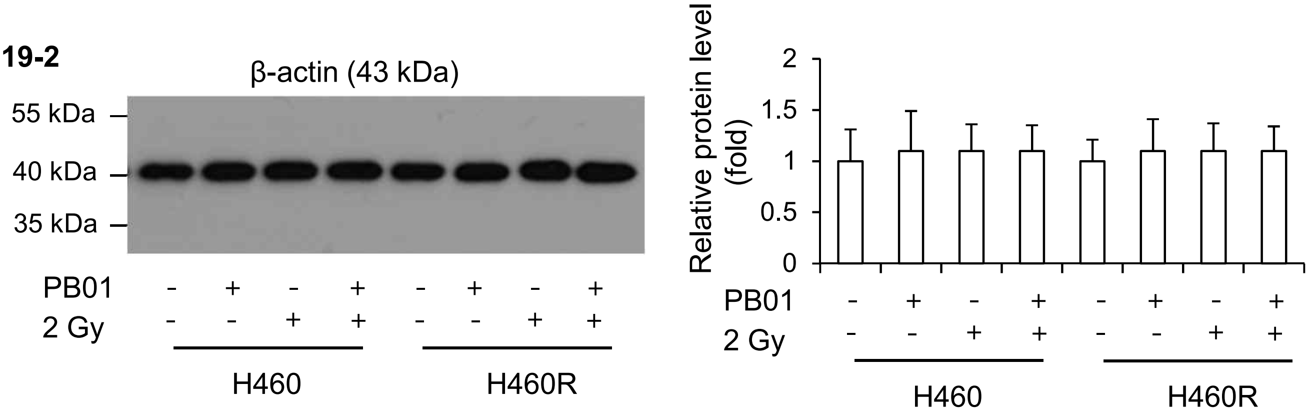

S19-3

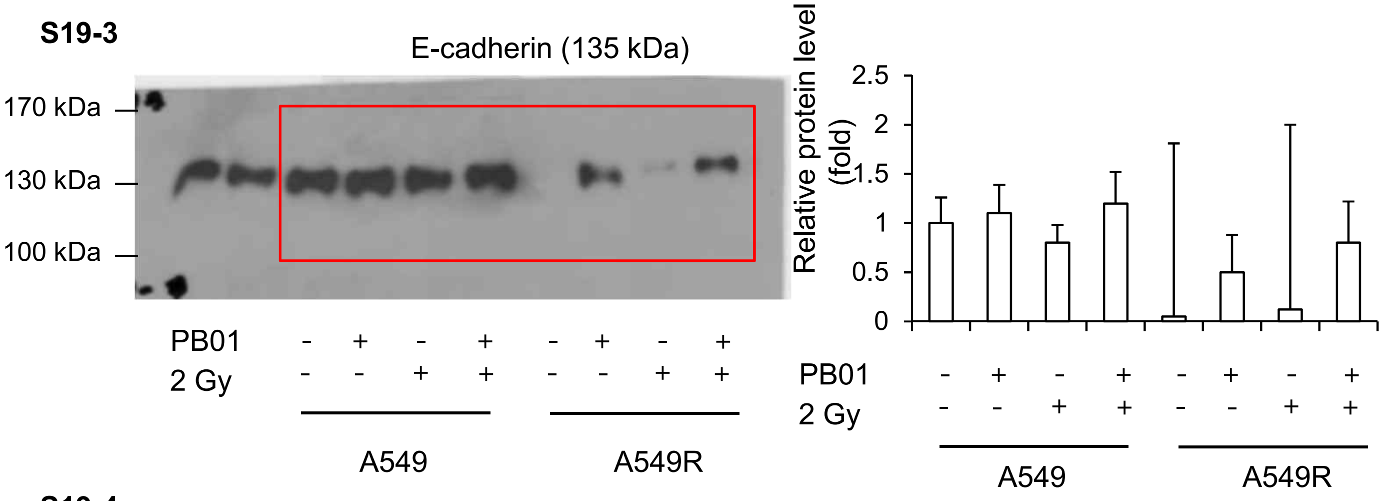

S19-4

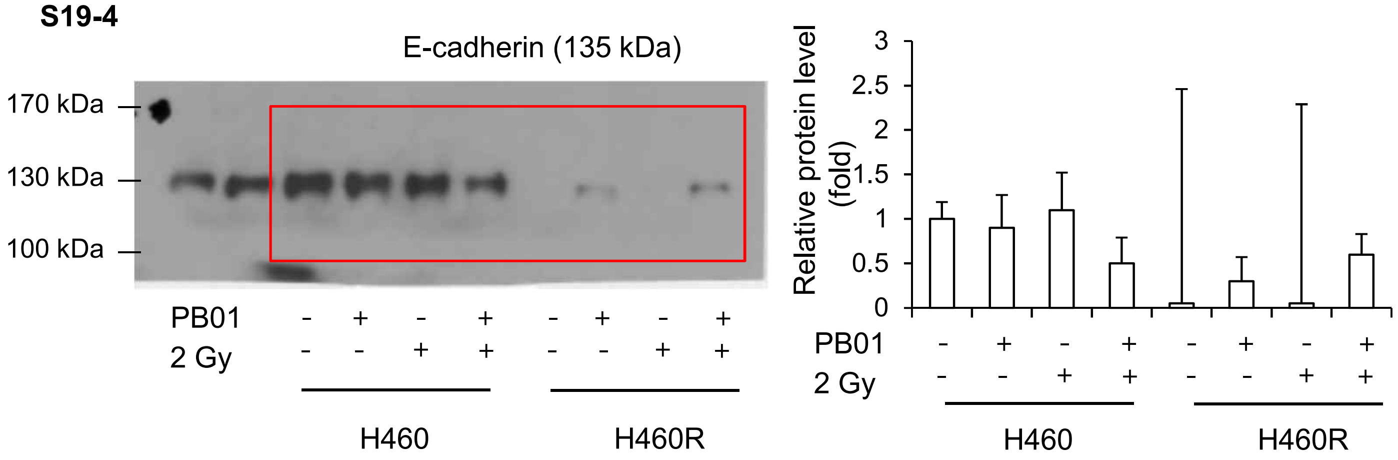

S19-5

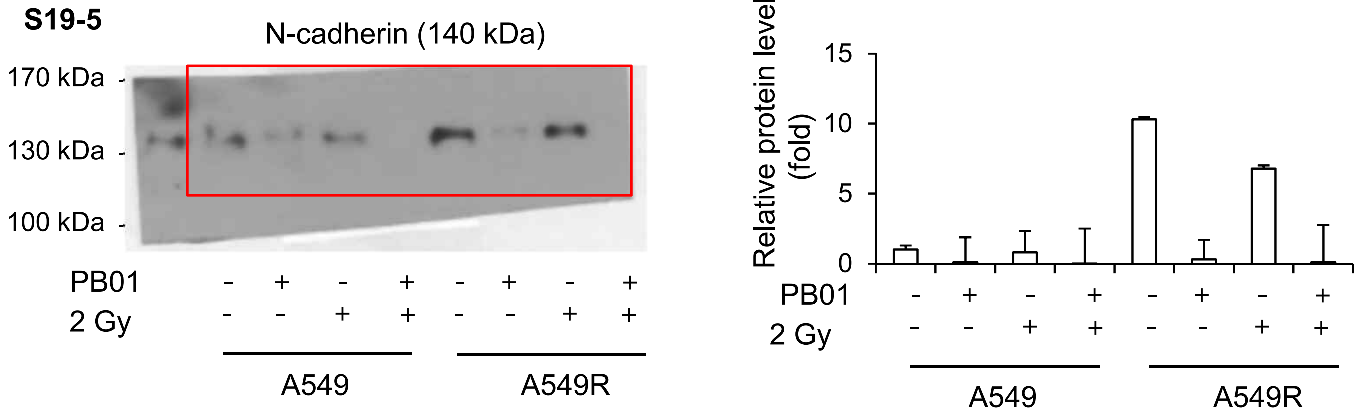

S19-6

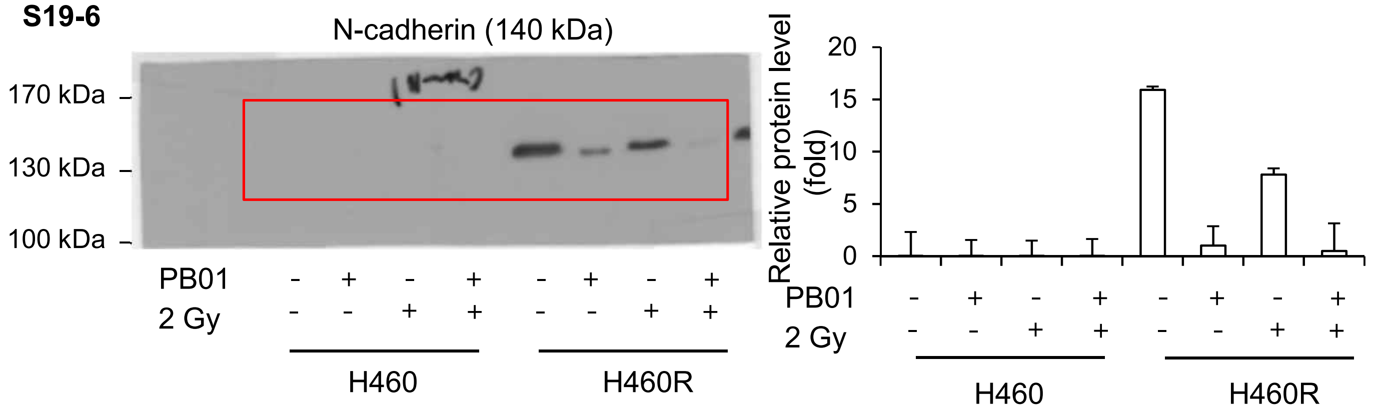

S19-7

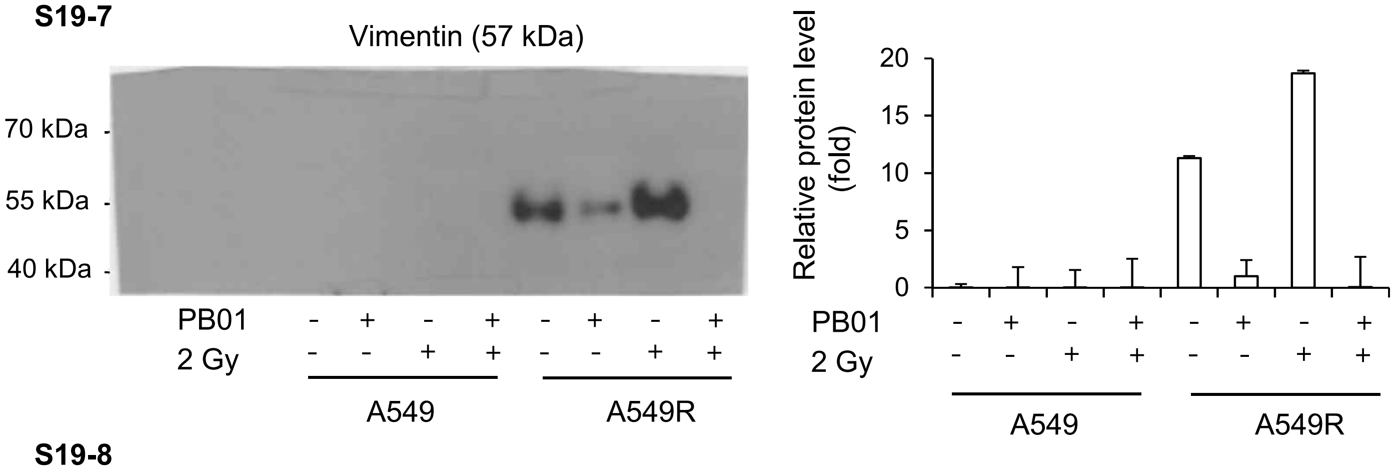

S19-8

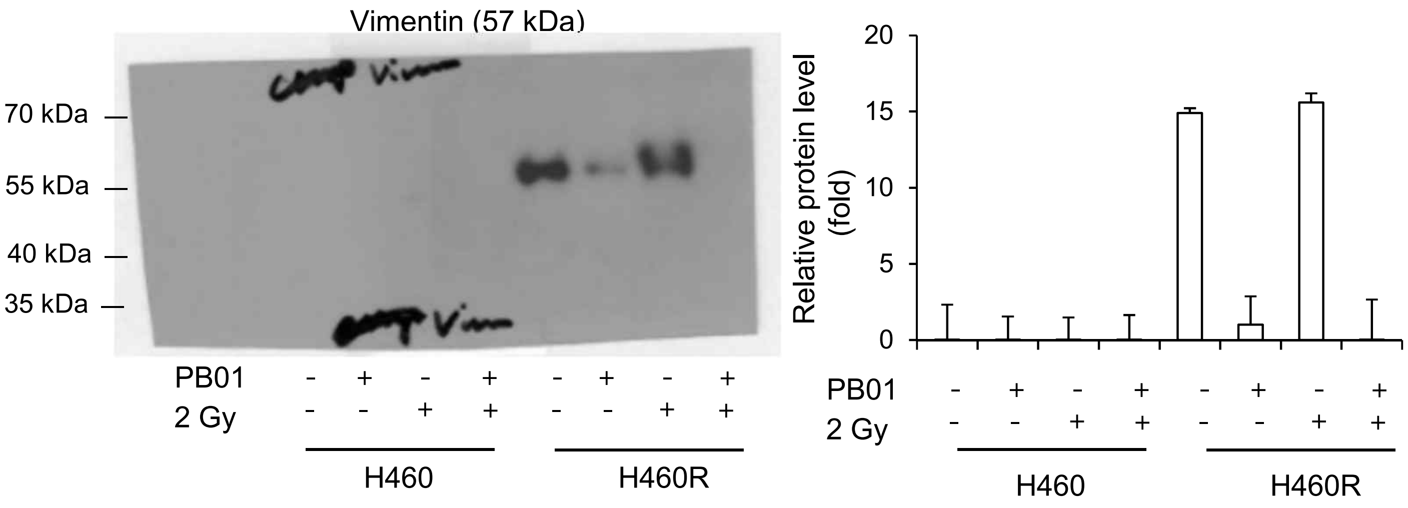

S19-9

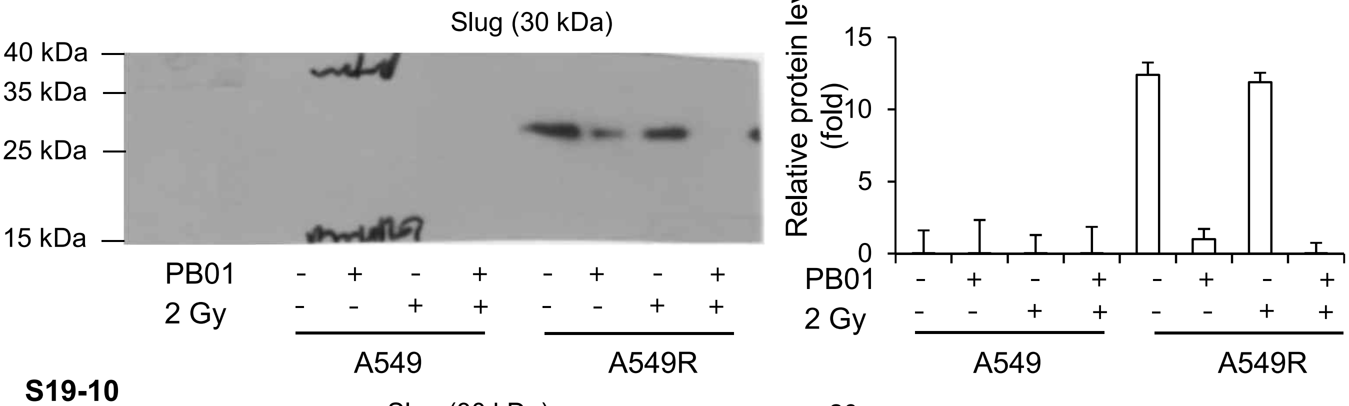

S19-10

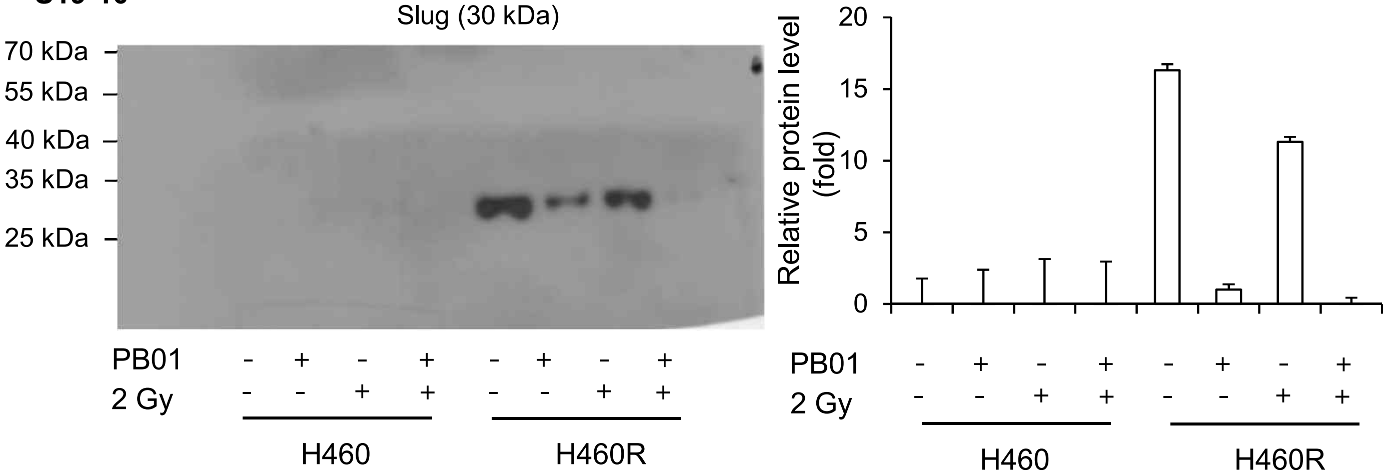

S19-11

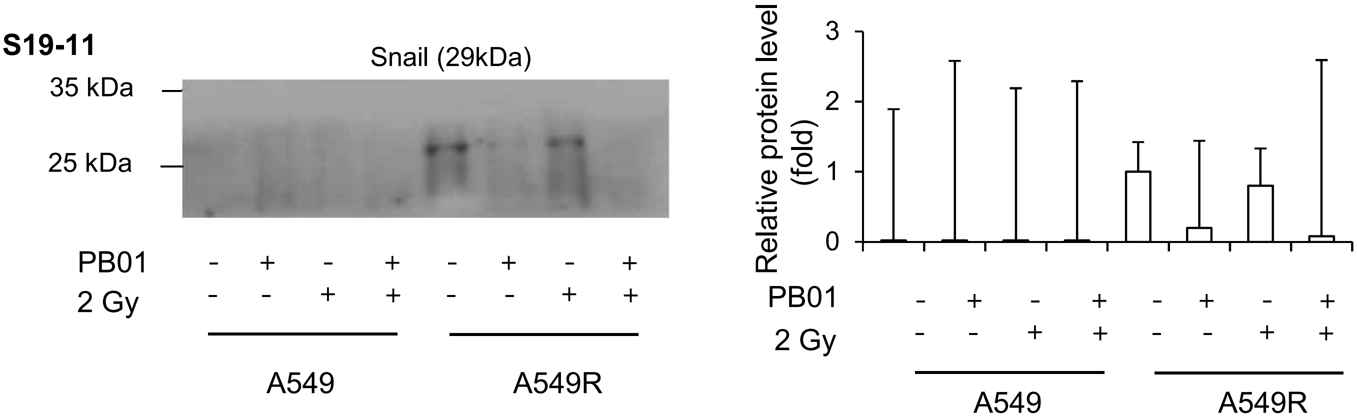

S19-12

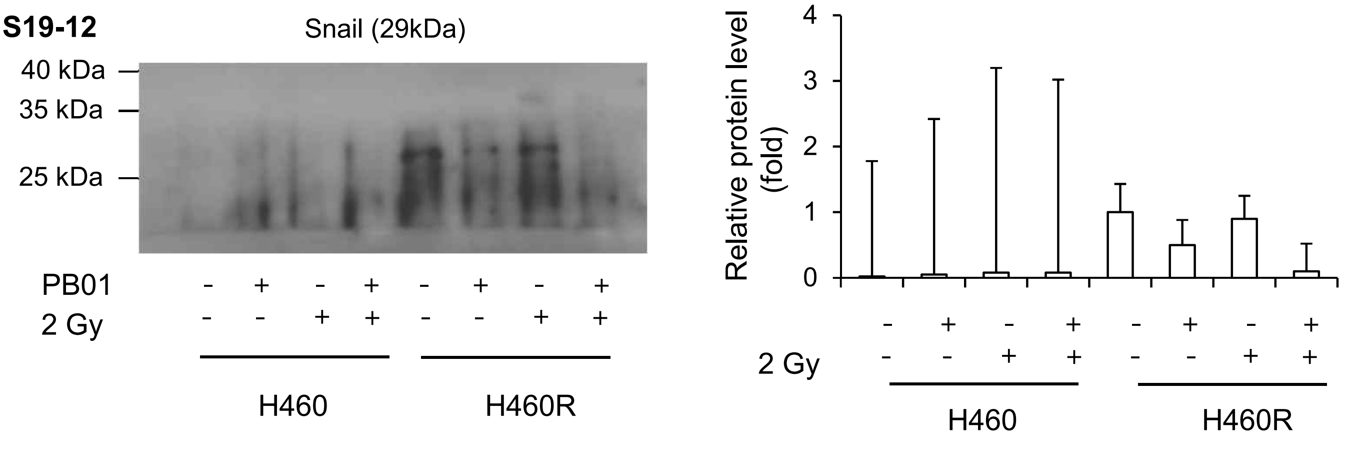

Figure S20. Supplementary materials for Figure 7b

S20-1

β-actin (43 kDa)

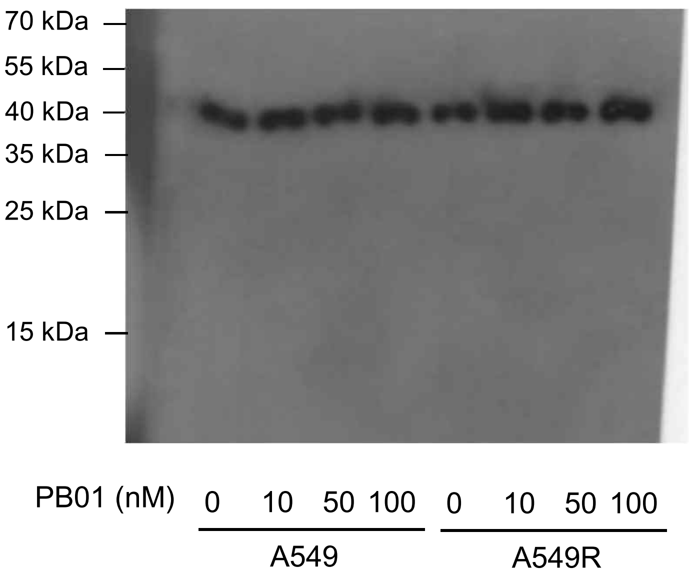

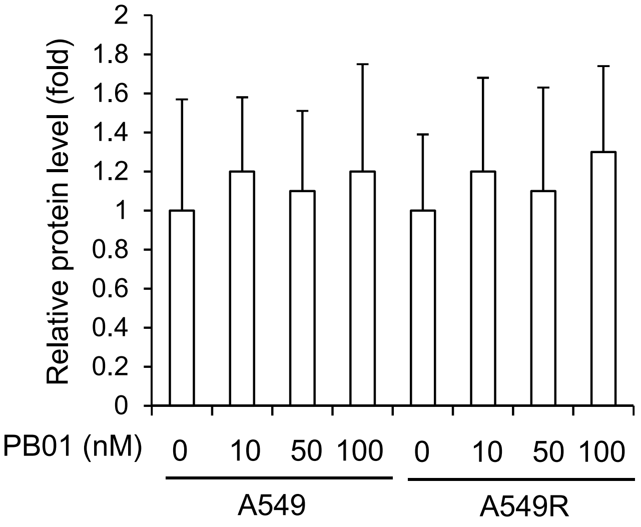

S20-2

β-actin (43 kDa)

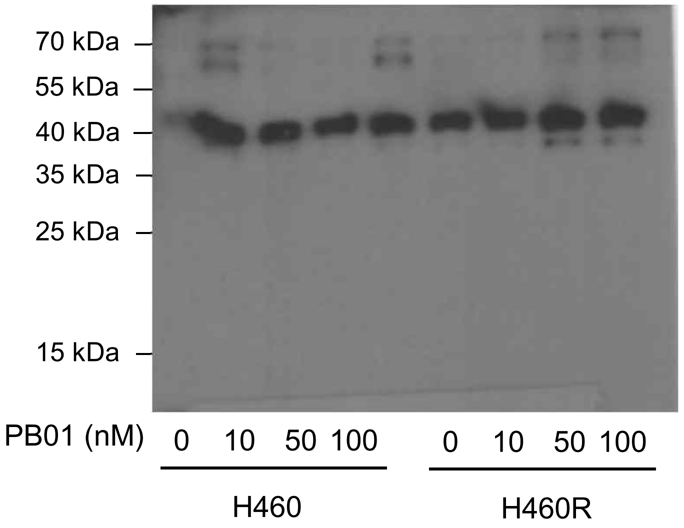

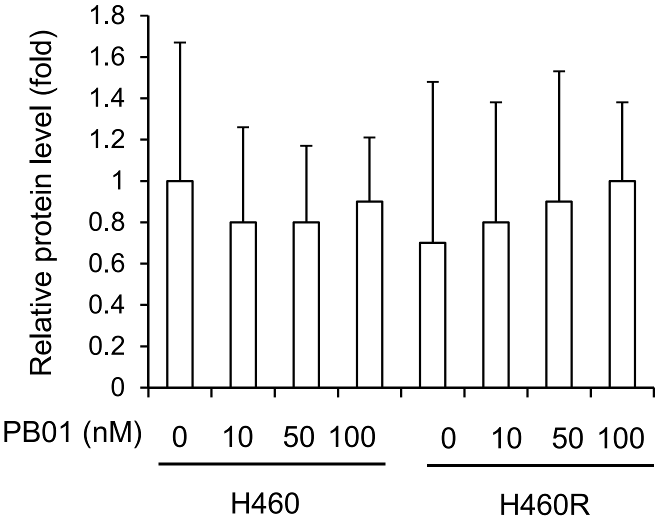

S20-3

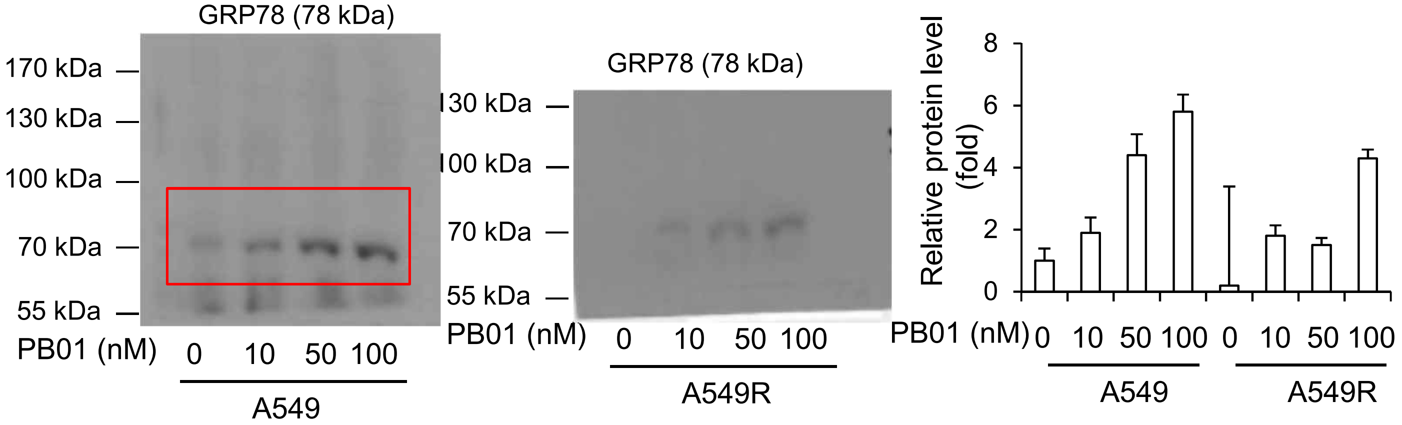

S20-4

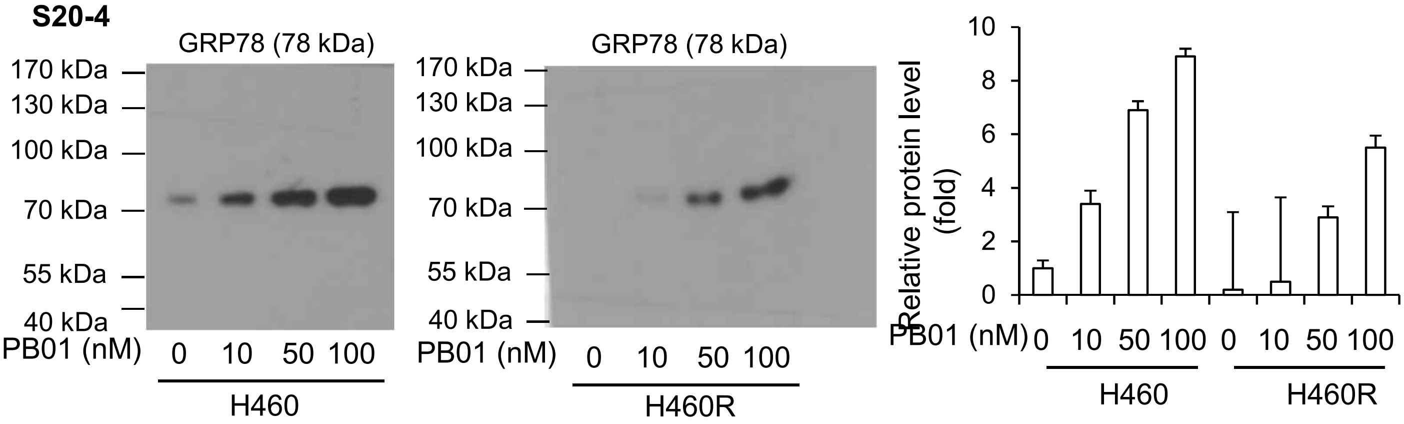

S20-5

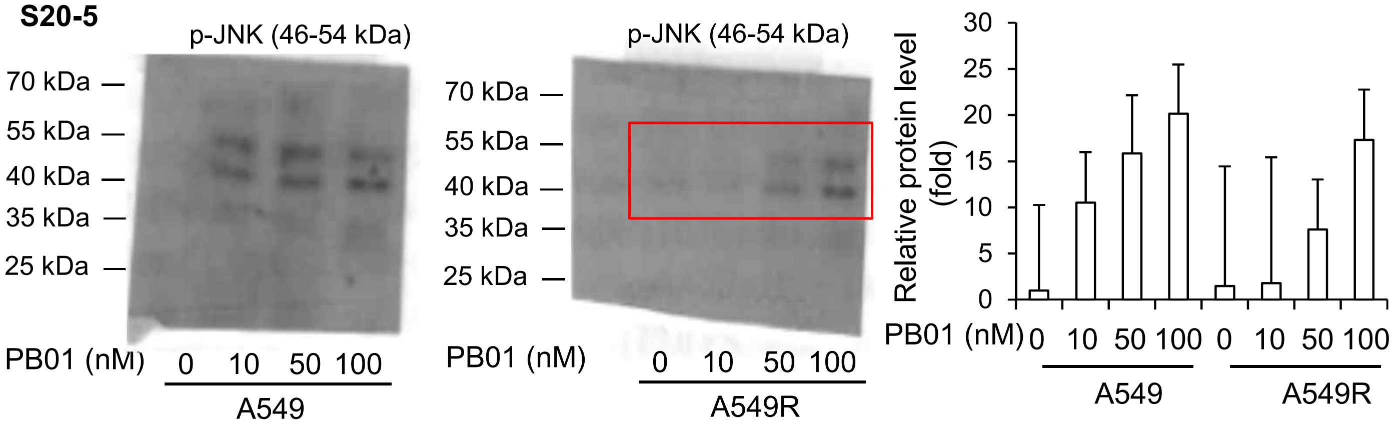

S20-6

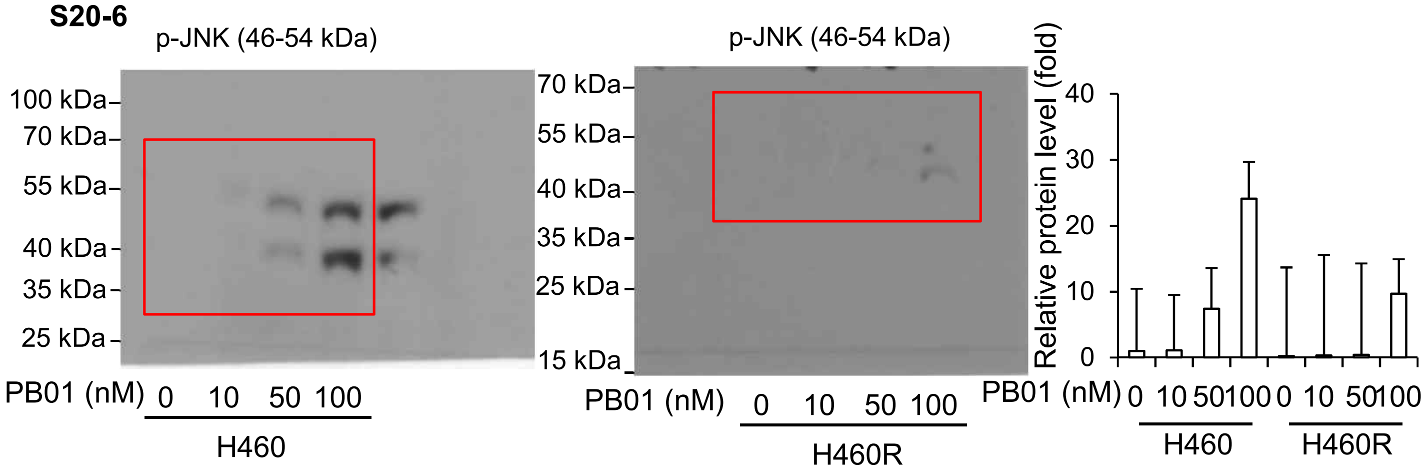

S20-7

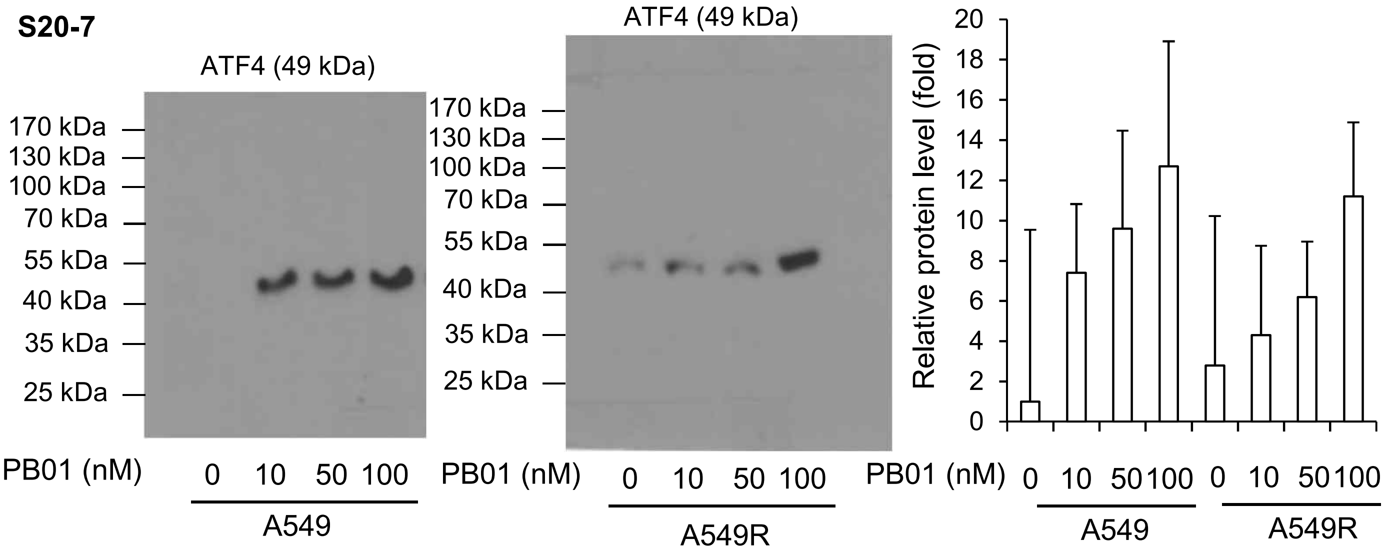

S20-8

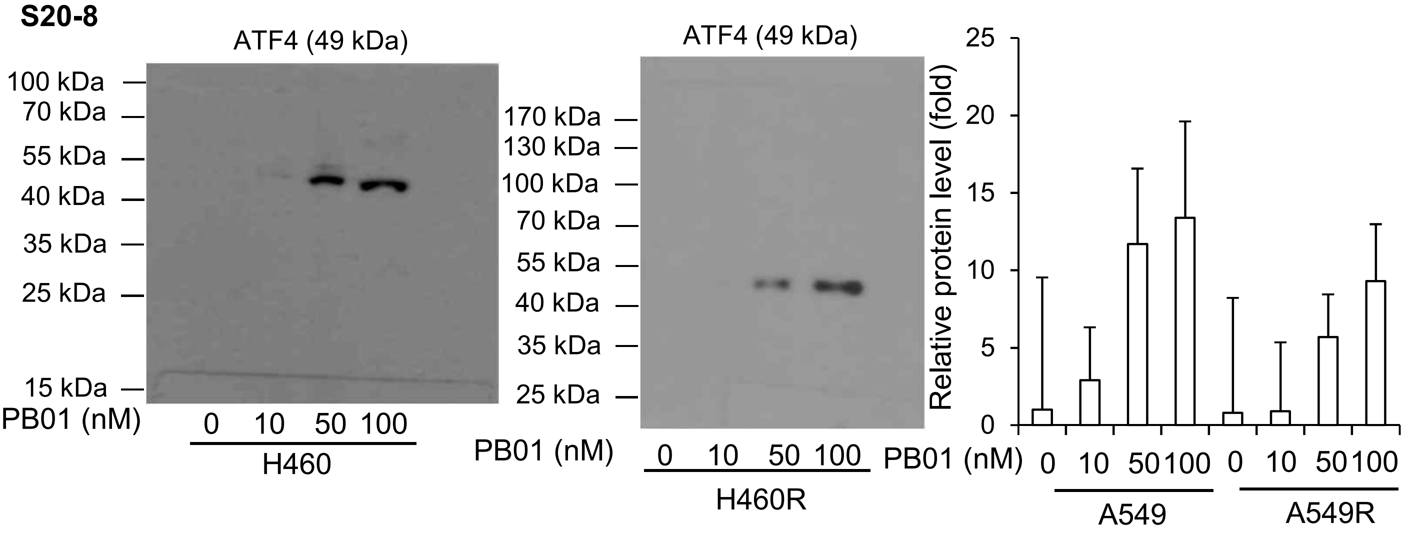

S20-9

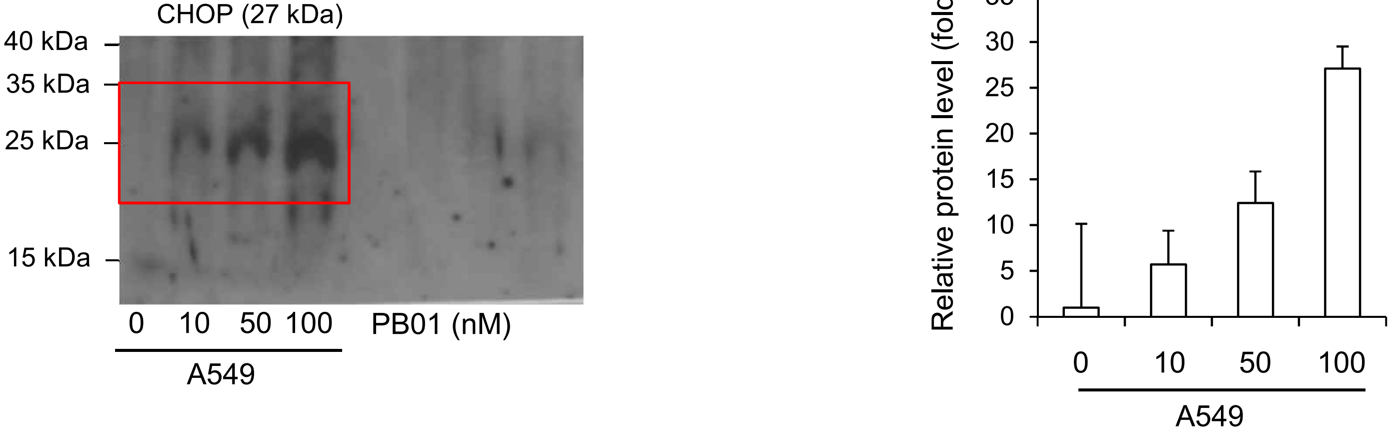

S20-10

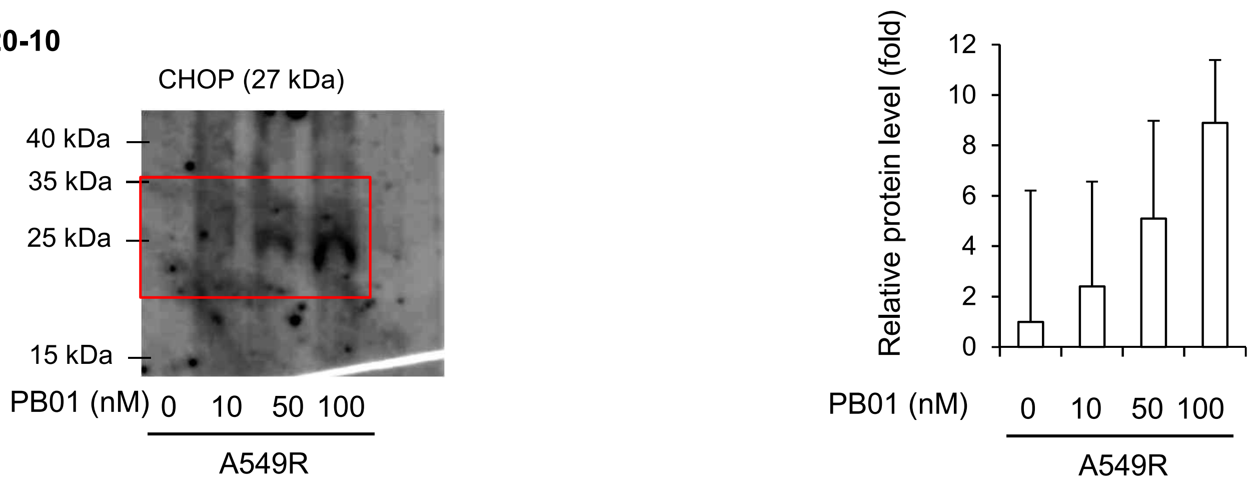

S20-11

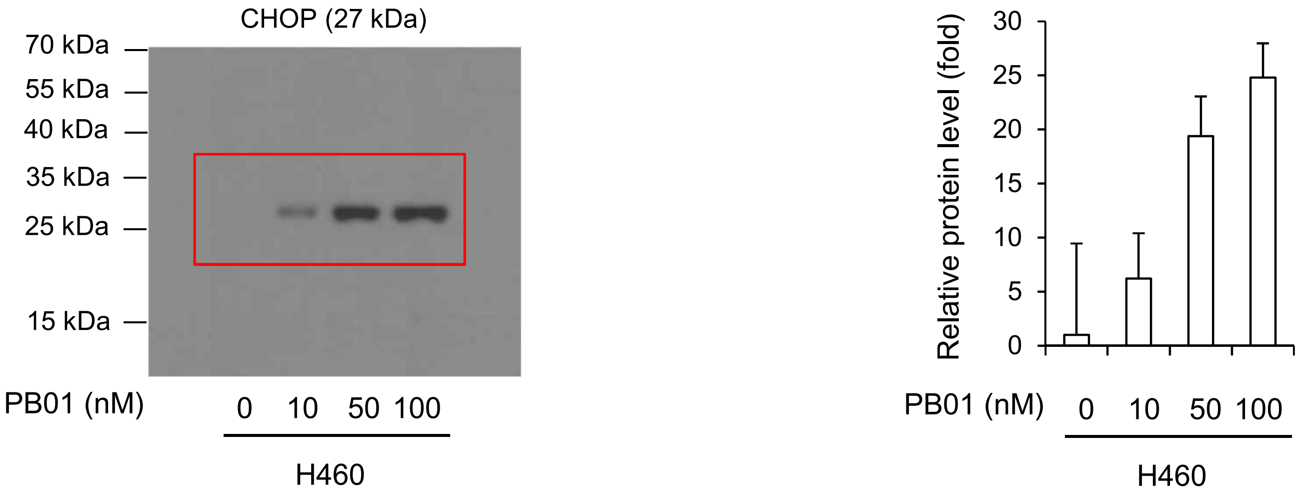

S20-12

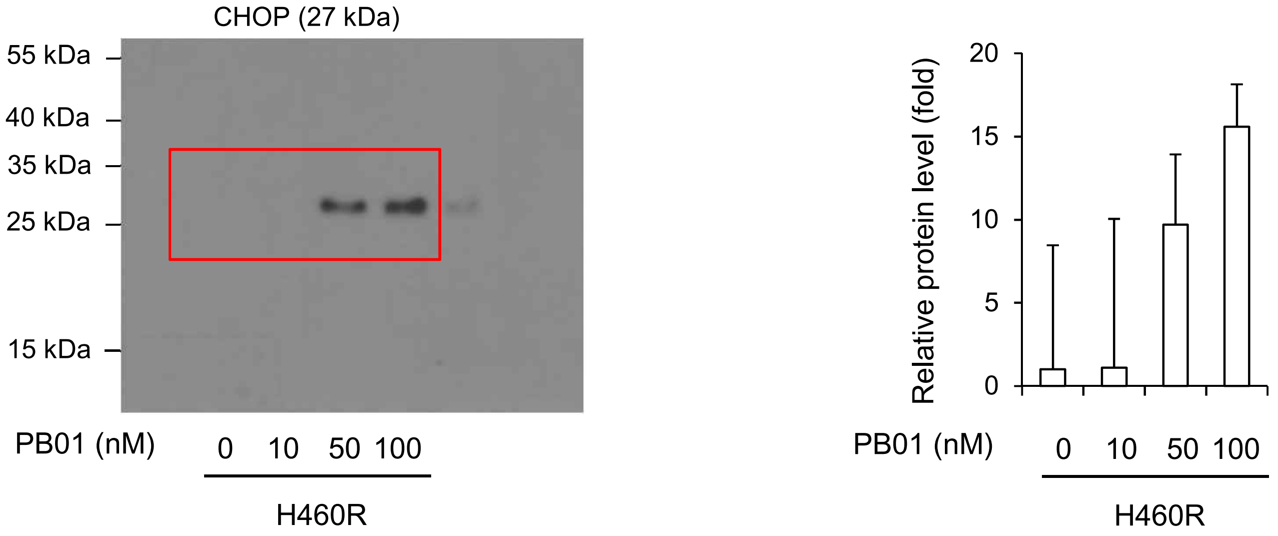

S20-13

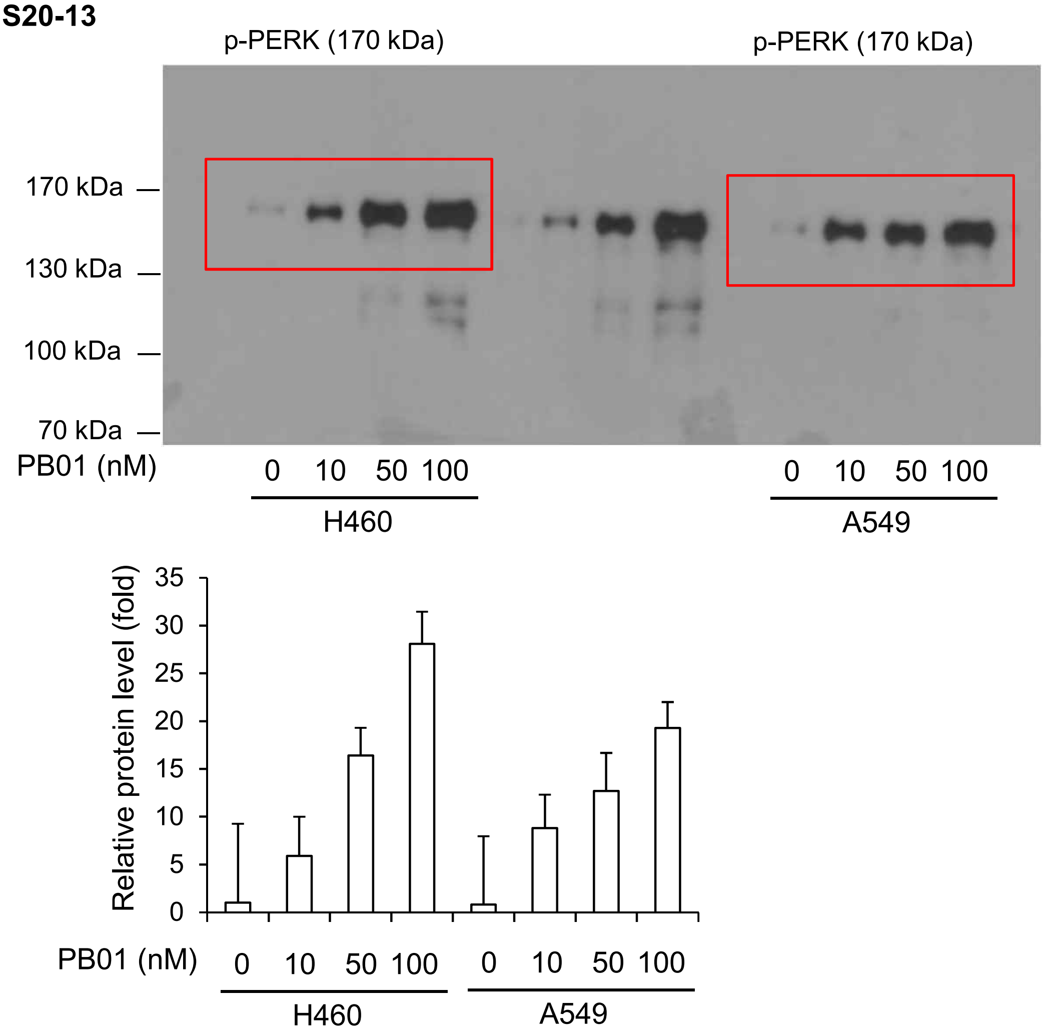
p-PERK (170 kDa)
p-PERK (170 kDa)
170 kDa
130 kDa
100 kDa
70 kDa
PB01 (nM)
0 10 50 100
H460
0 10 50 100
A549
Relative protein level (fold)
35
30
25
20
15
10
5
0
PB01 (nM)
0 10 50 100
H460
0 10 50 100
A549

S20-14

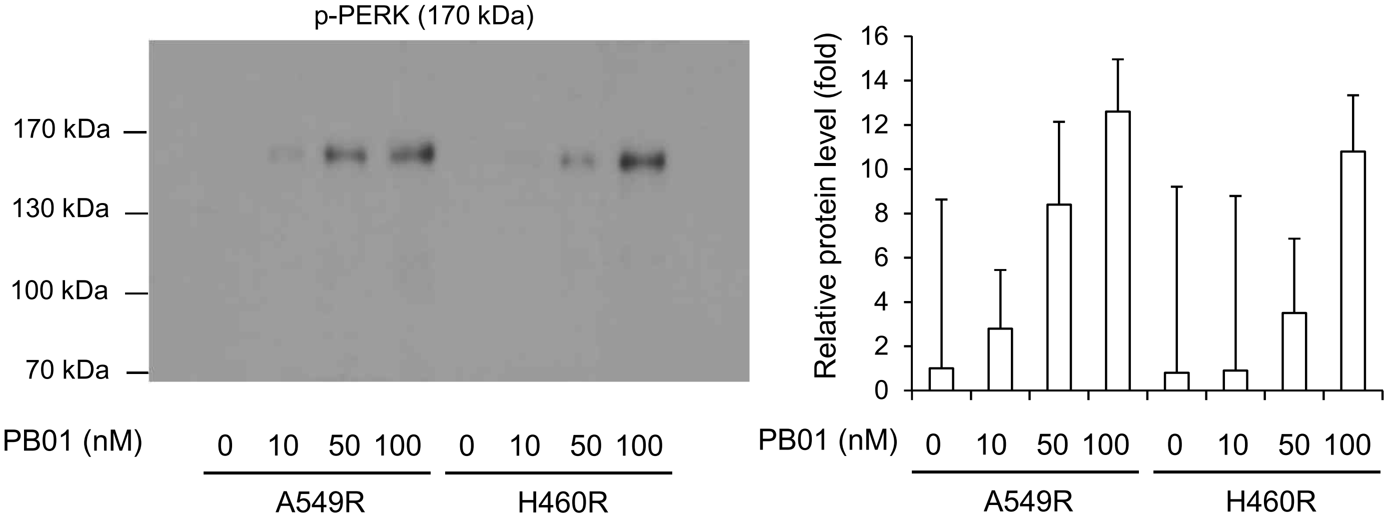
p-PERK (170 kDa)
170 kDa
130 kDa
100 kDa
70 kDa
PB01 (nM)
0 10 50 100
A549R
0 10 50 100
H460R
Relative protein level (fold)
16
14
12
10
8
6
4
2
0
PB01 (nM)
0 10 50 100
A549R
0 10 50 100
H460R

S20-15

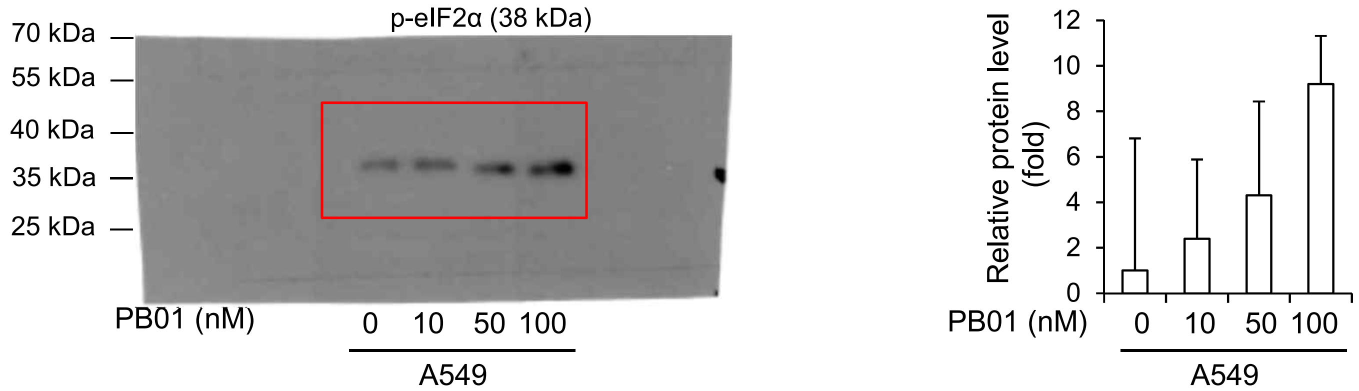

S20-16

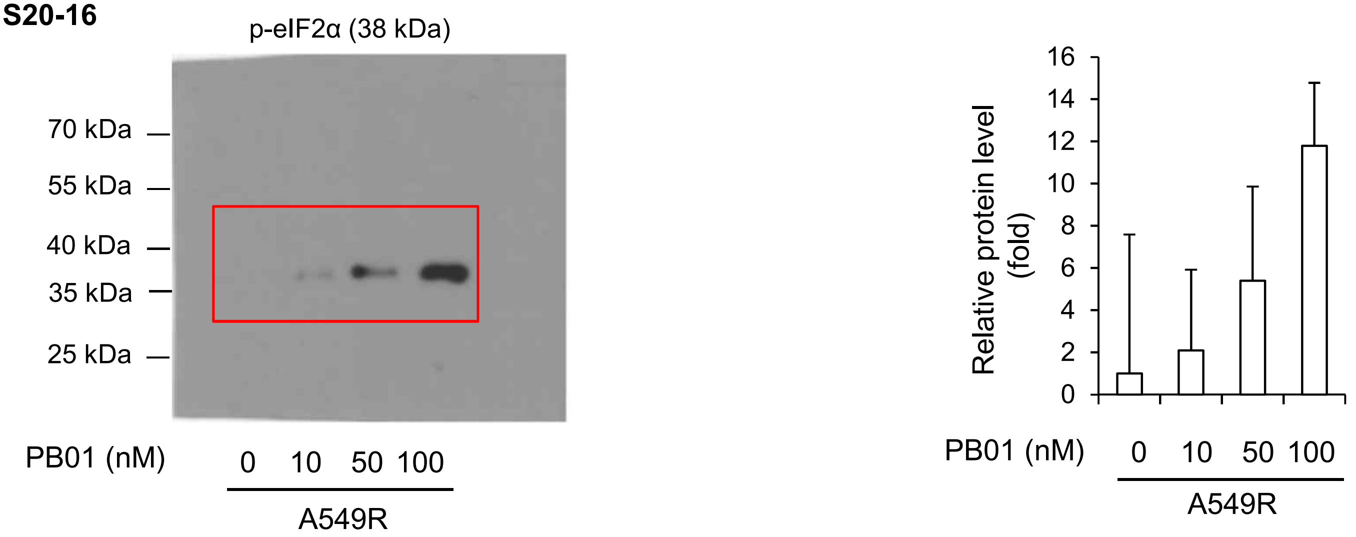

S20-17

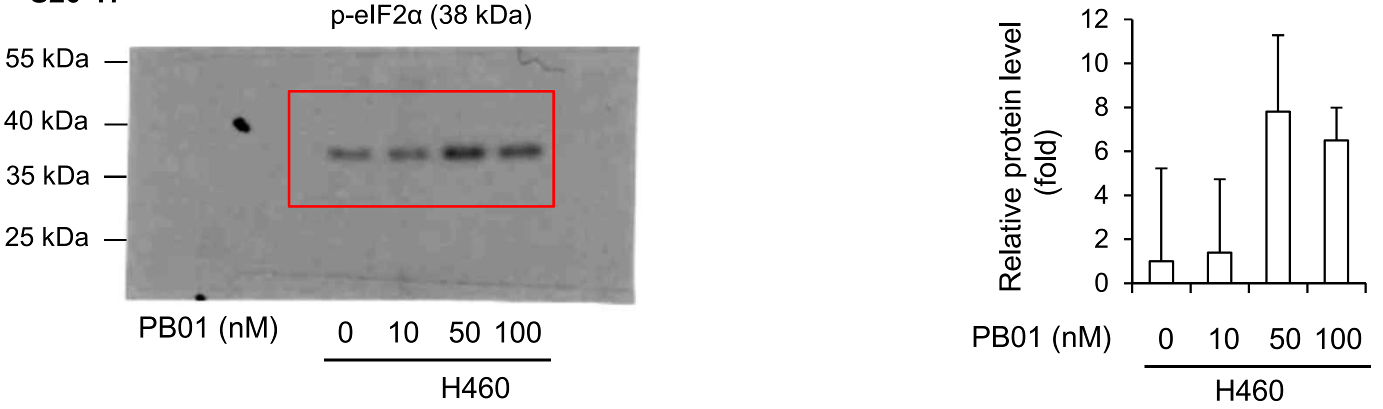

S20-18

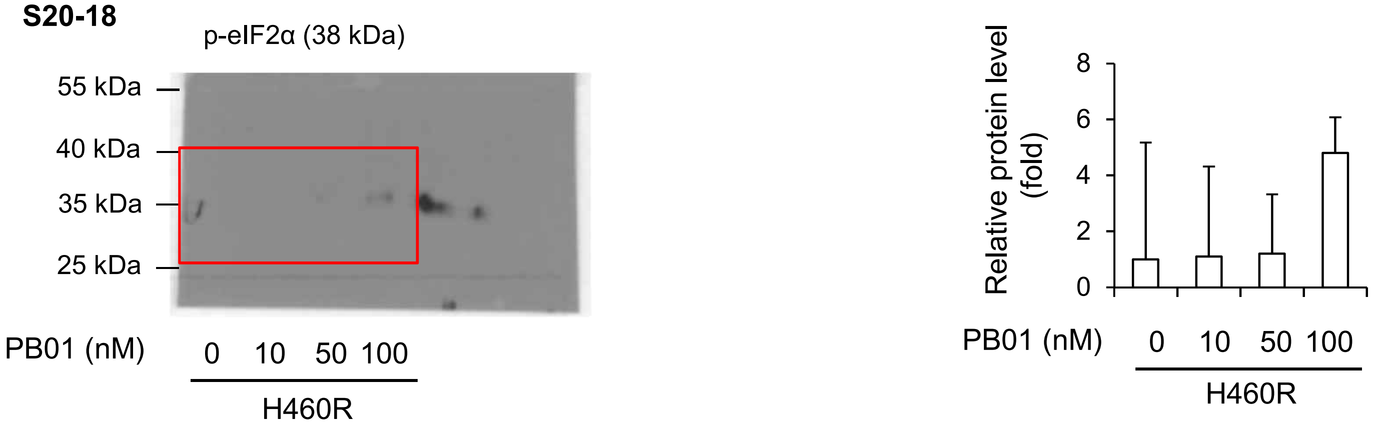

S20-19

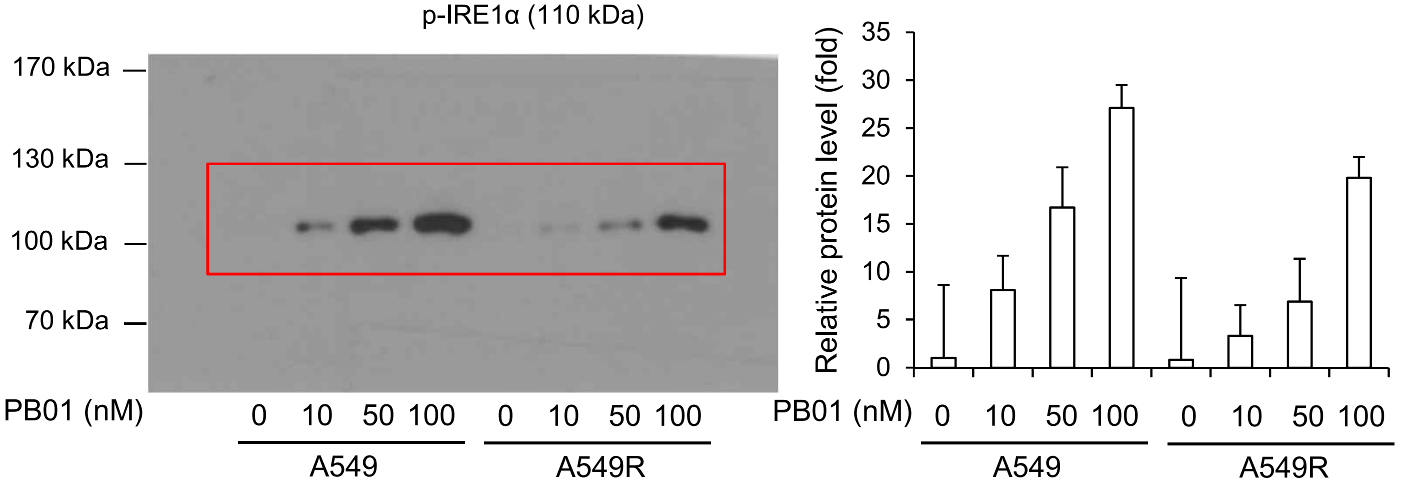
p-IRE1α (110 kDa)
170 kDa
130 kDa
100 kDa
70 kDa
PB01 (nM)
0 10 50 100 0 10 50 100
A549
A549R
Relative protein level (fold)
35
30
25
20
15
10
5
0
PB01 (nM)
0 10 50 100 0 10 50 100
A549
A549R

S20-20

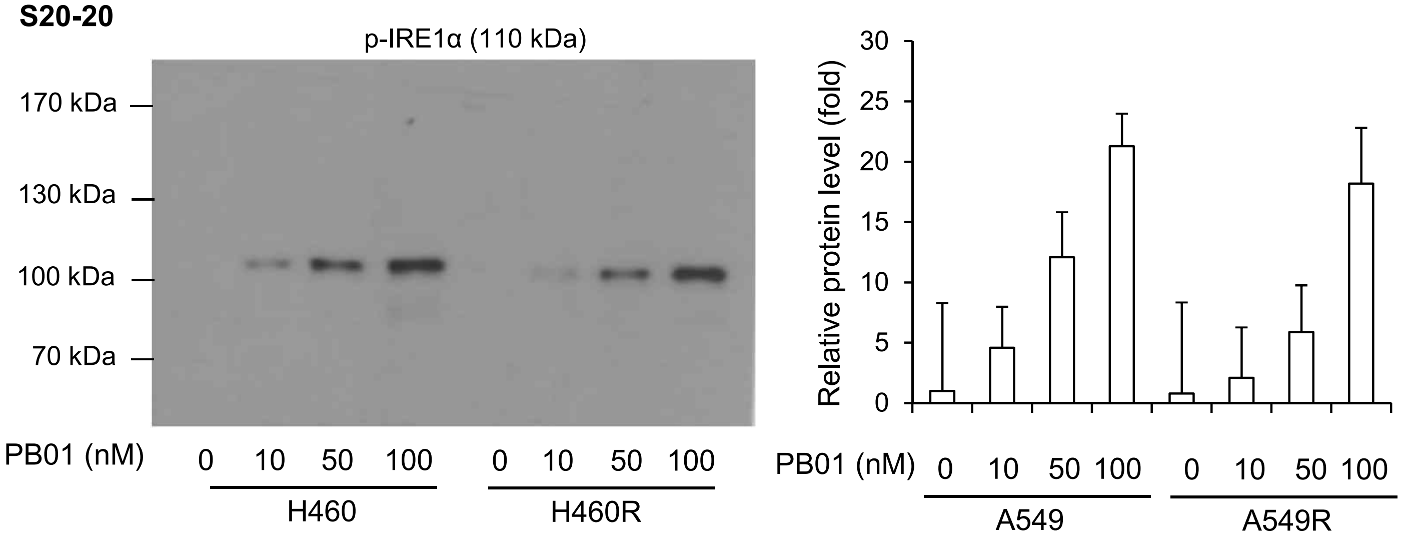
p-IRE1α (110 kDa)
170 kDa
130 kDa
100 kDa
70 kDa
PB01 (nM)
0 10 50 100 0 10 50 100
H460
H460R
Relative protein level (fold)
30
25
20
15
10
5
0
PB01 (nM)
0 10 50 100 0 10 50 100
A549
A549R

Figure S21. Supplementary materials for Figure 7e

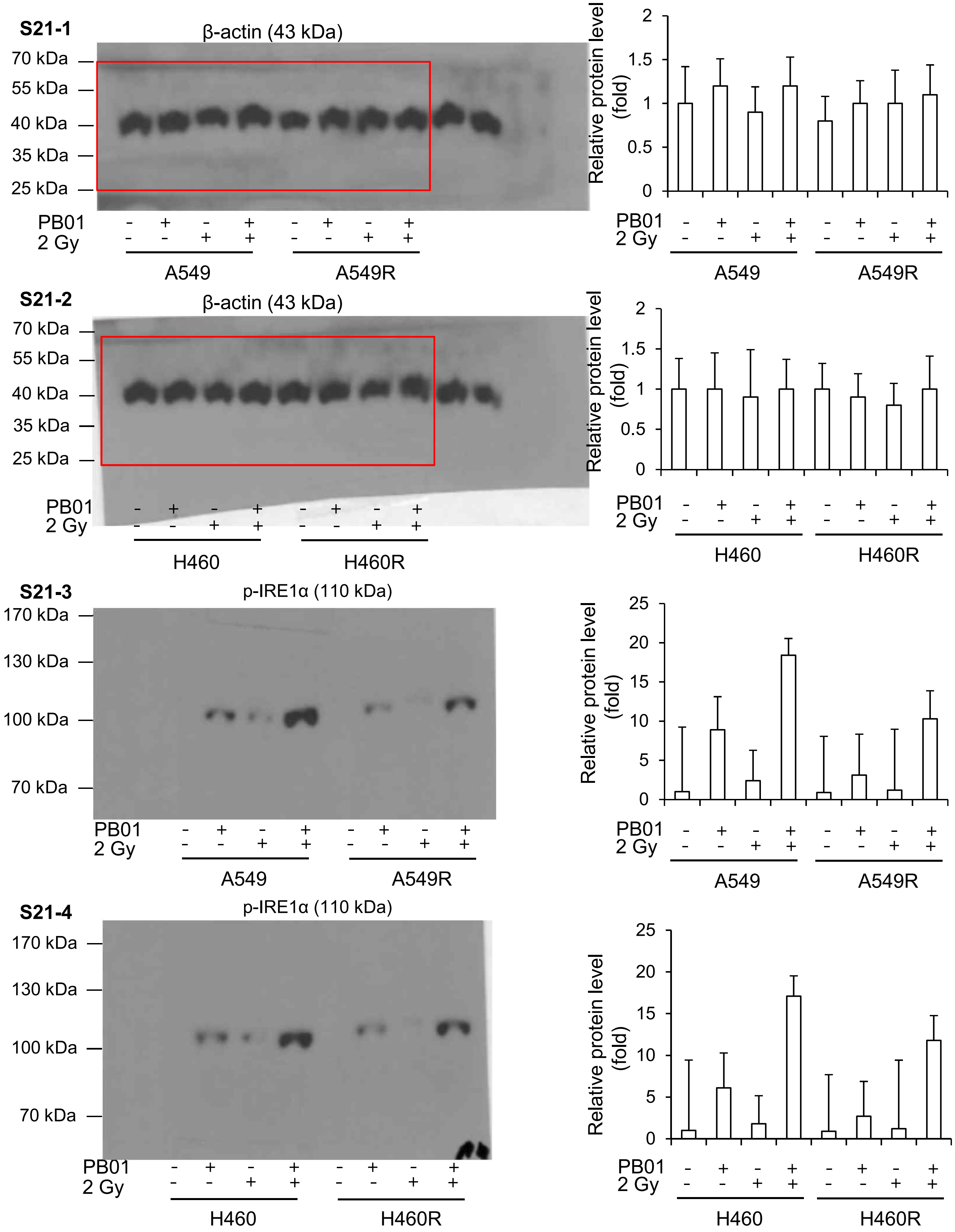

S21-5

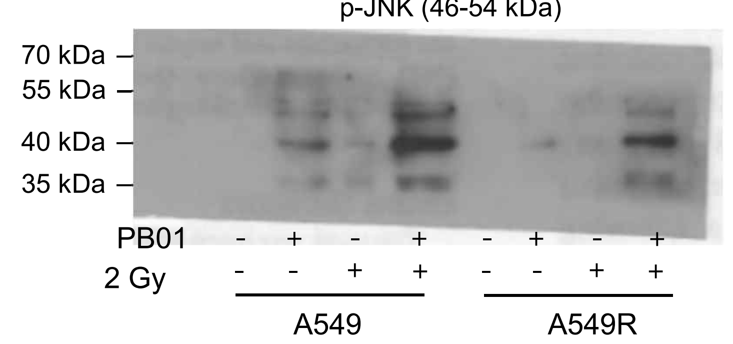

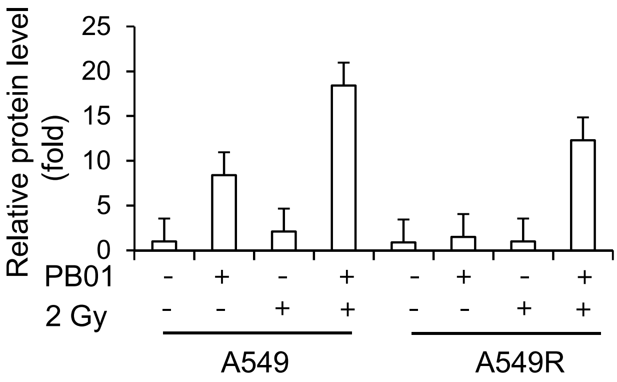

S21-6

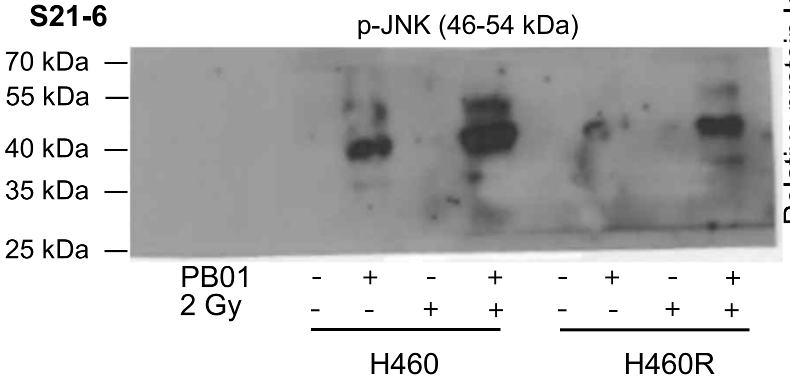

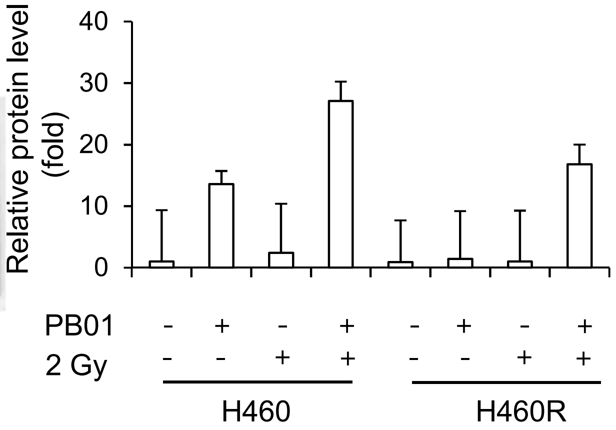

S21-7

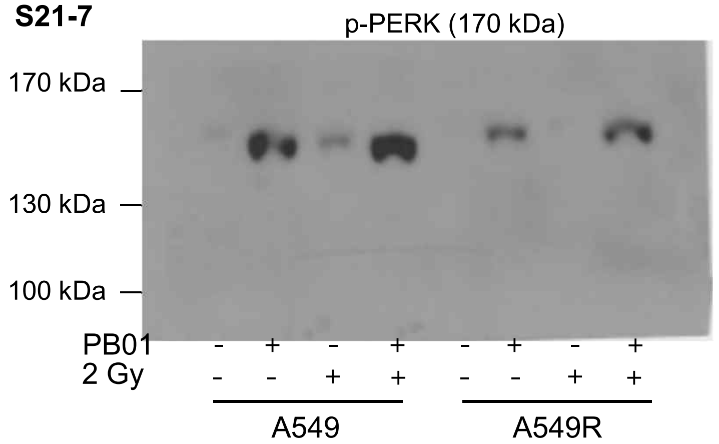

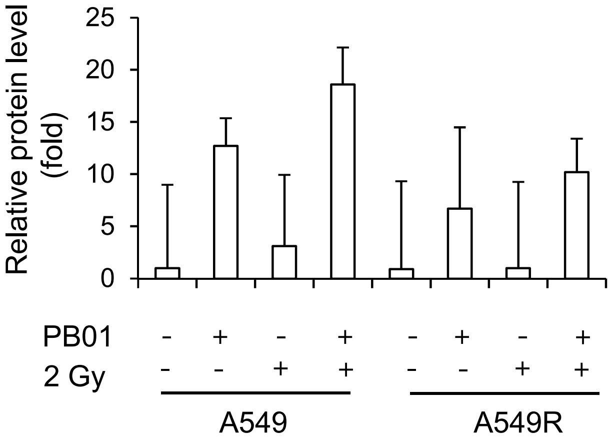

S21-8

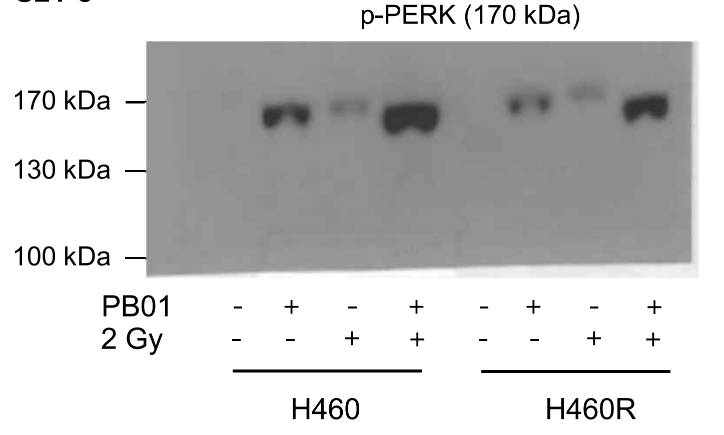

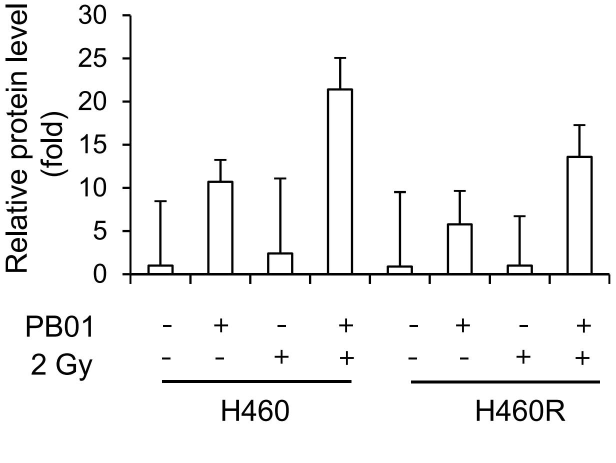

S21-9

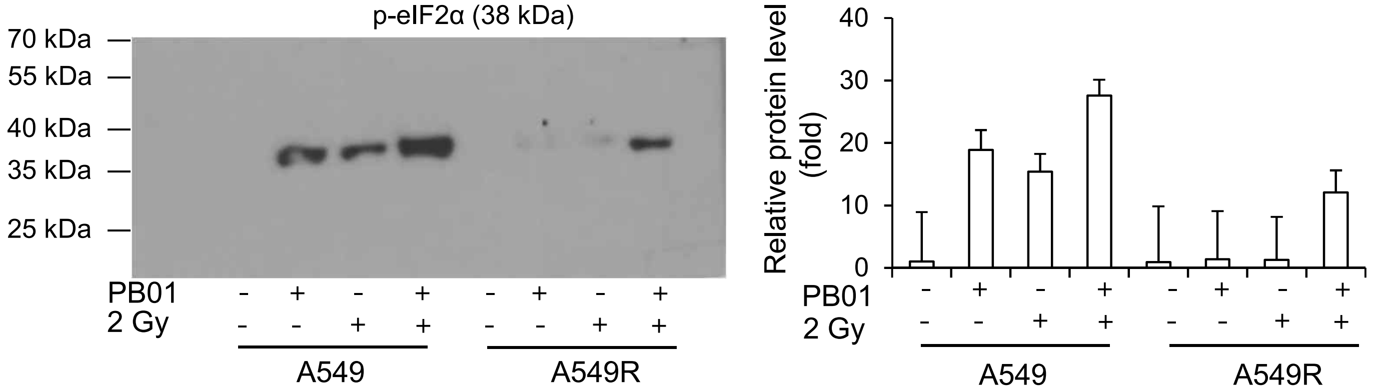

S21-10

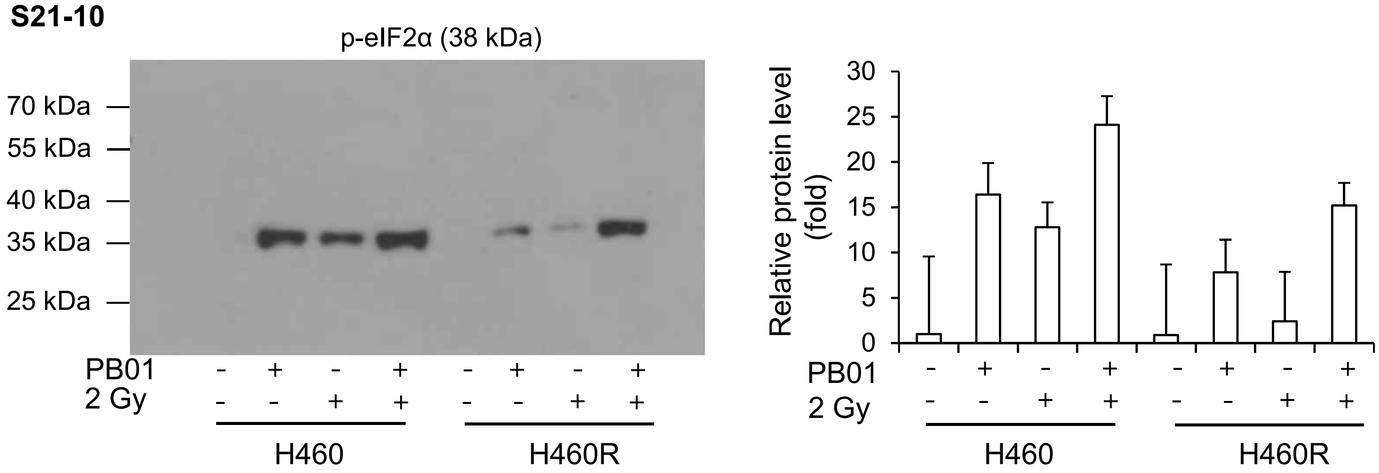

S21-11

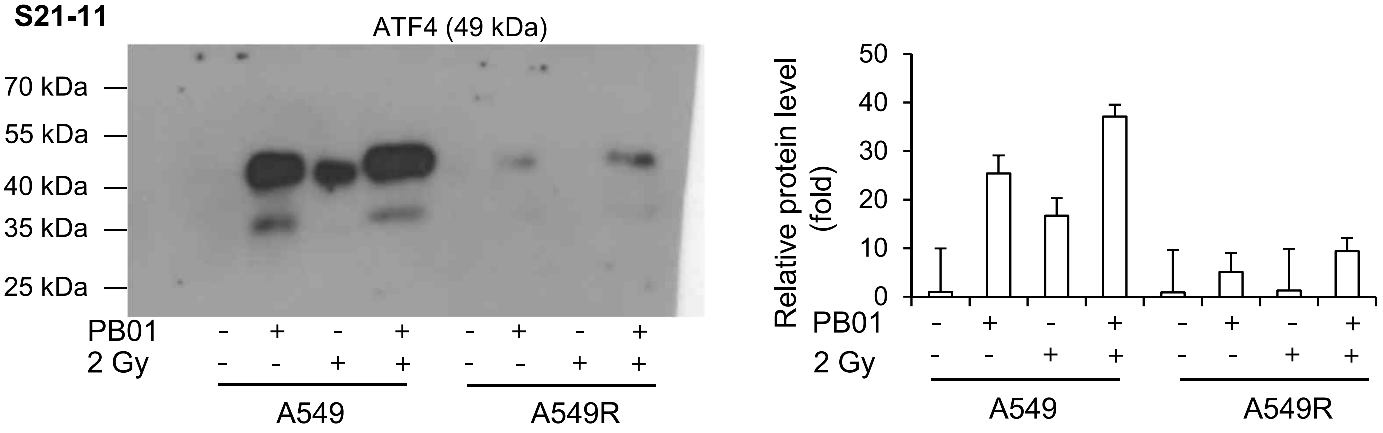

S21-12

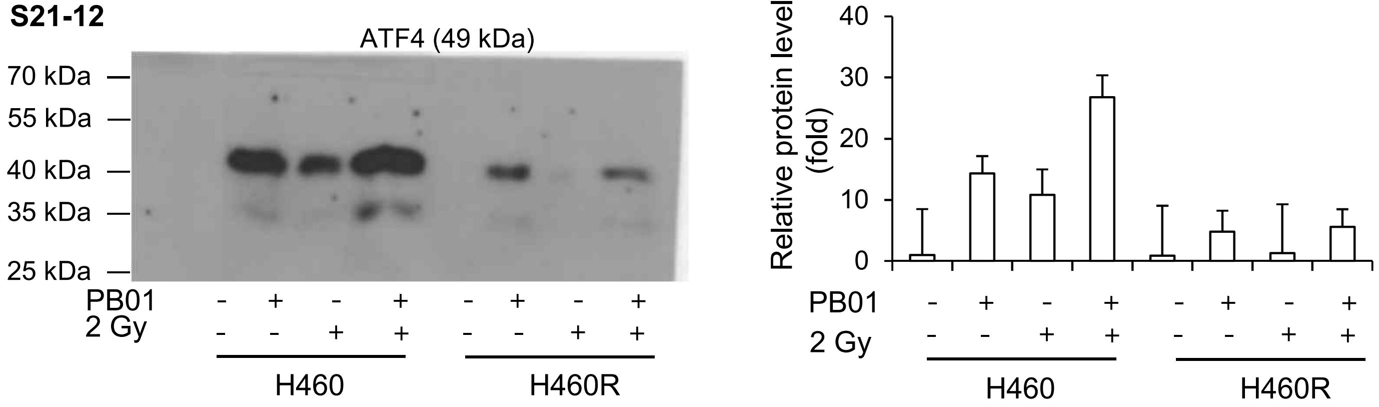

S21-13

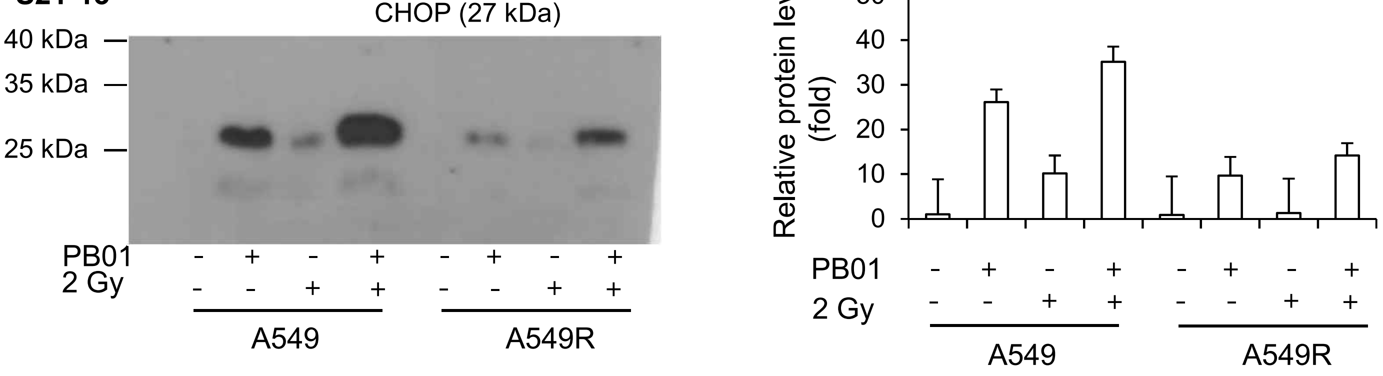

S21-14

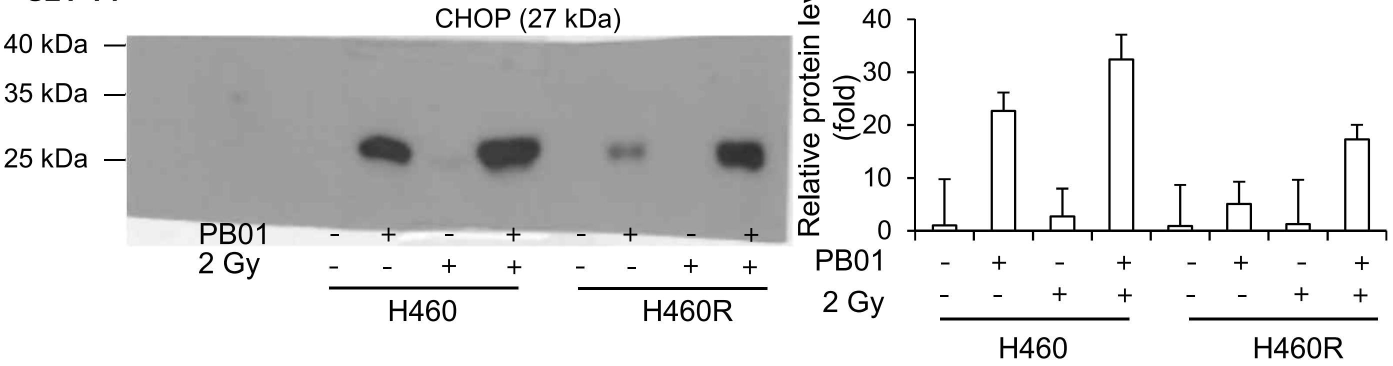

S21-15

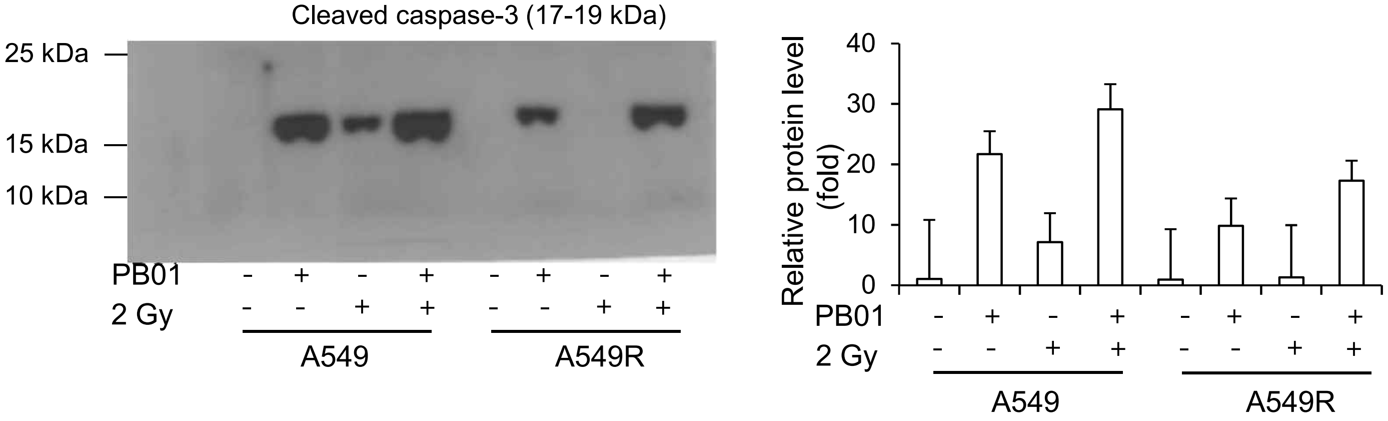

S21-16

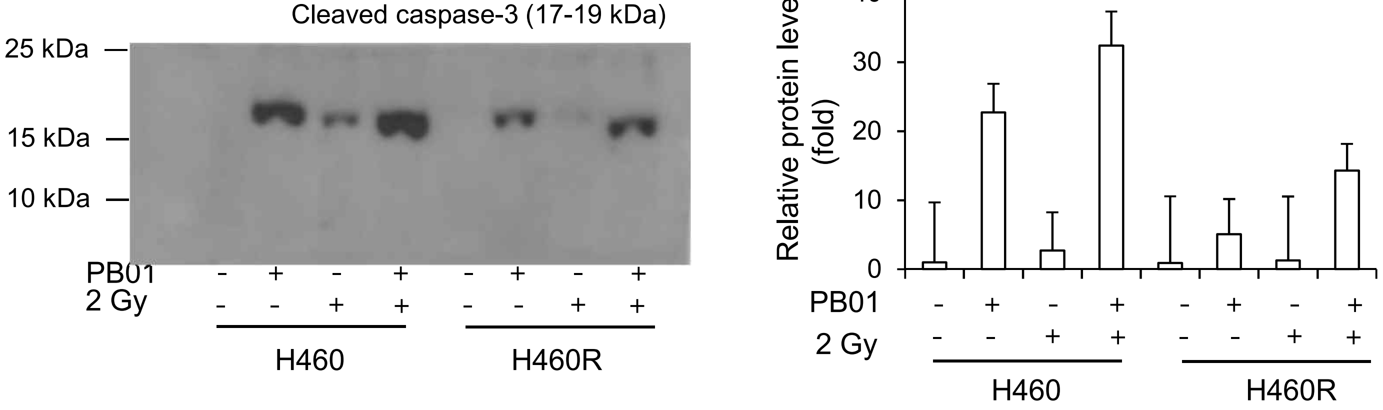

Figure S22. Supplementary materials for Figure 8c

S22-1

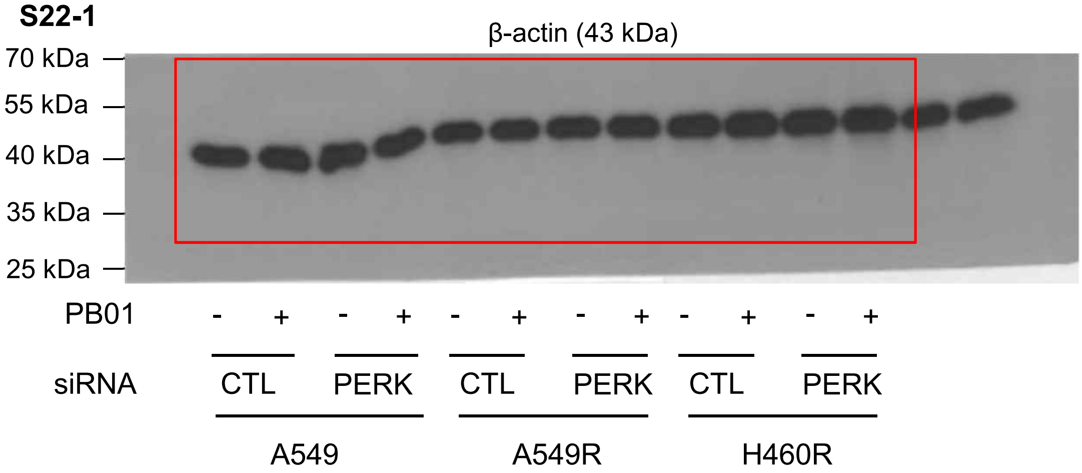

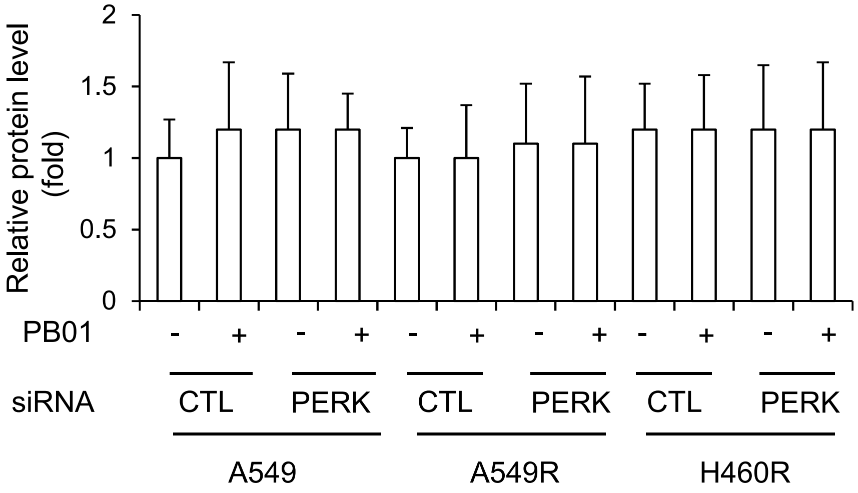

S22-2

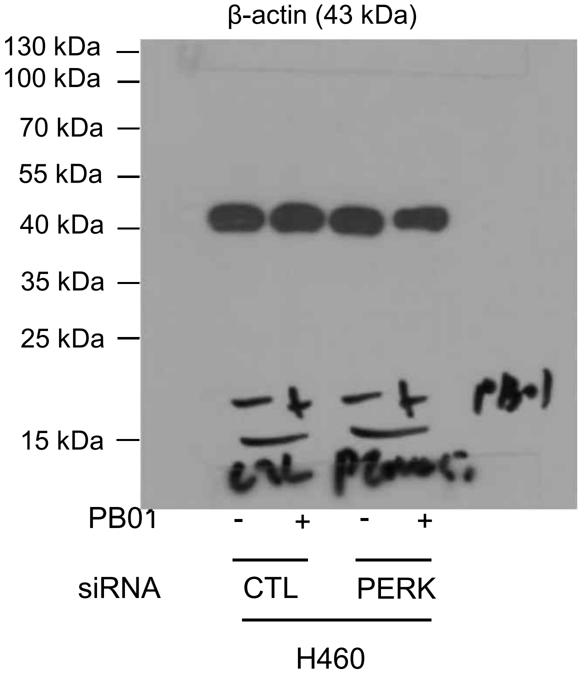

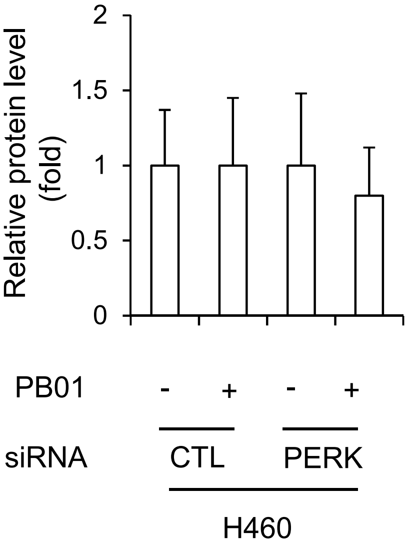

S22-3

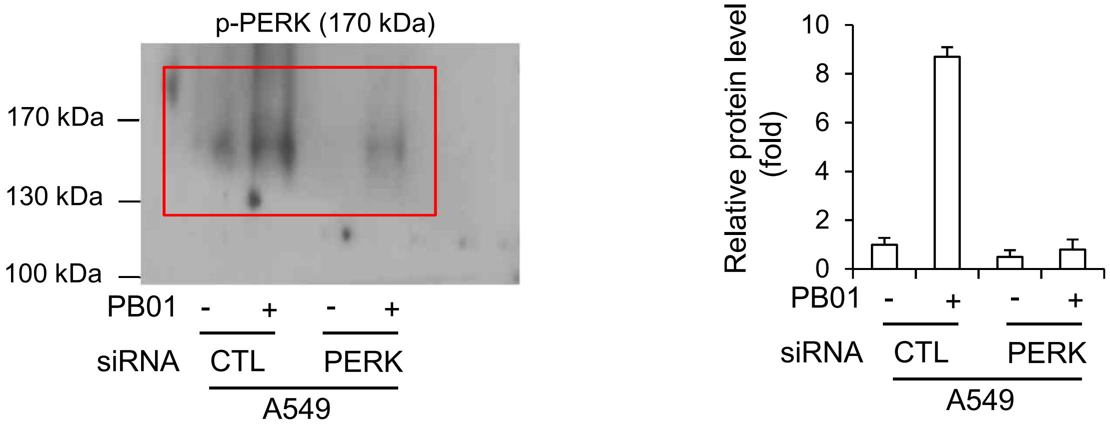

S22-4

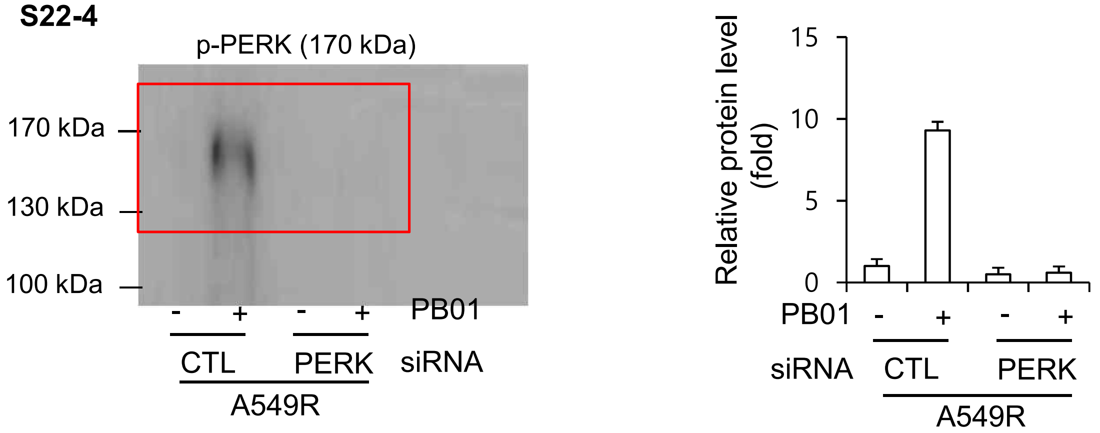

S22-5

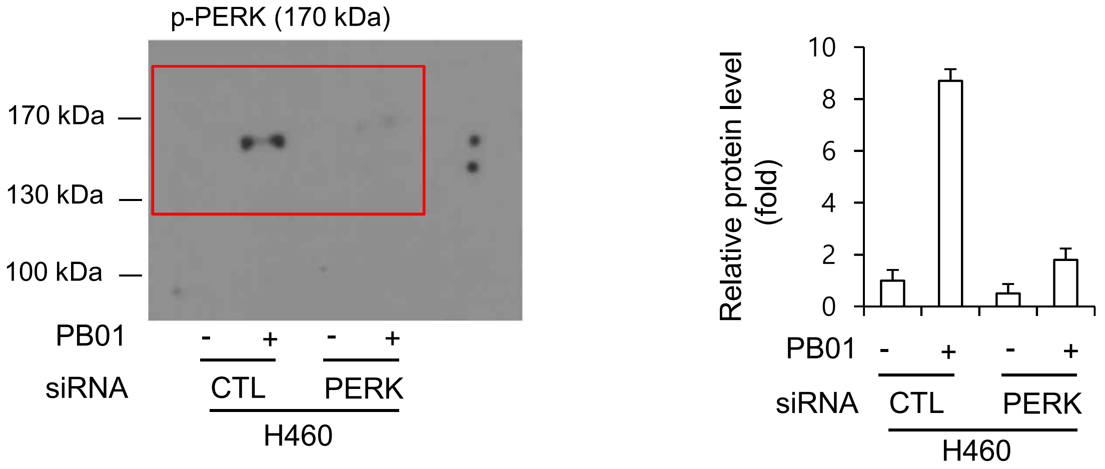

S22-6

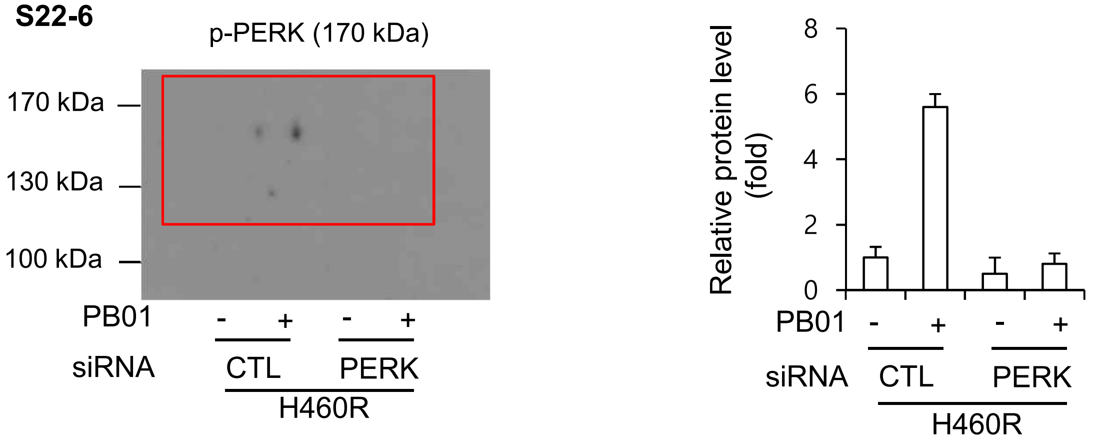

S22-7

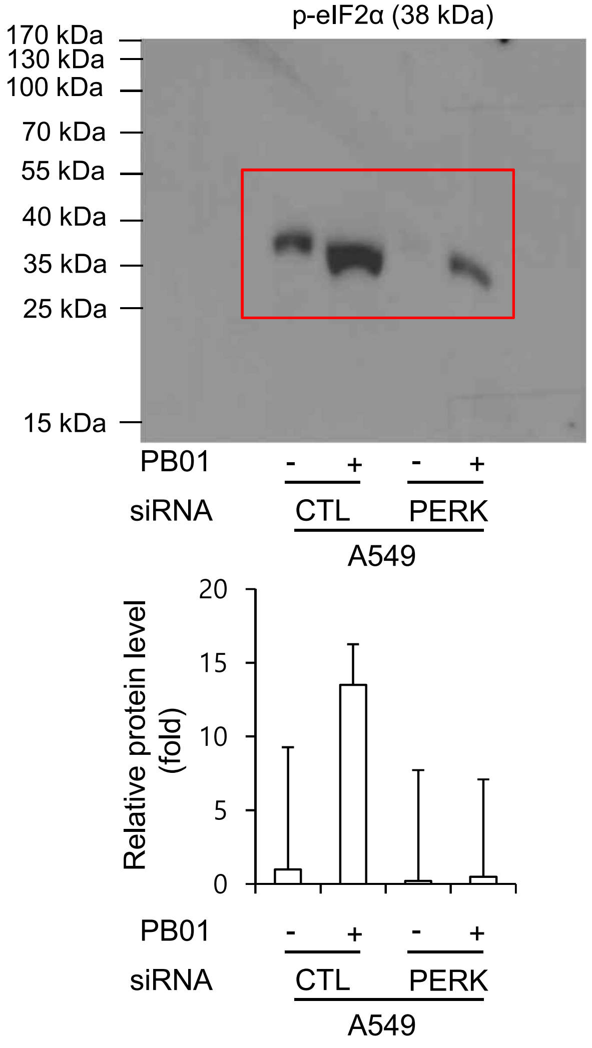

S22-8

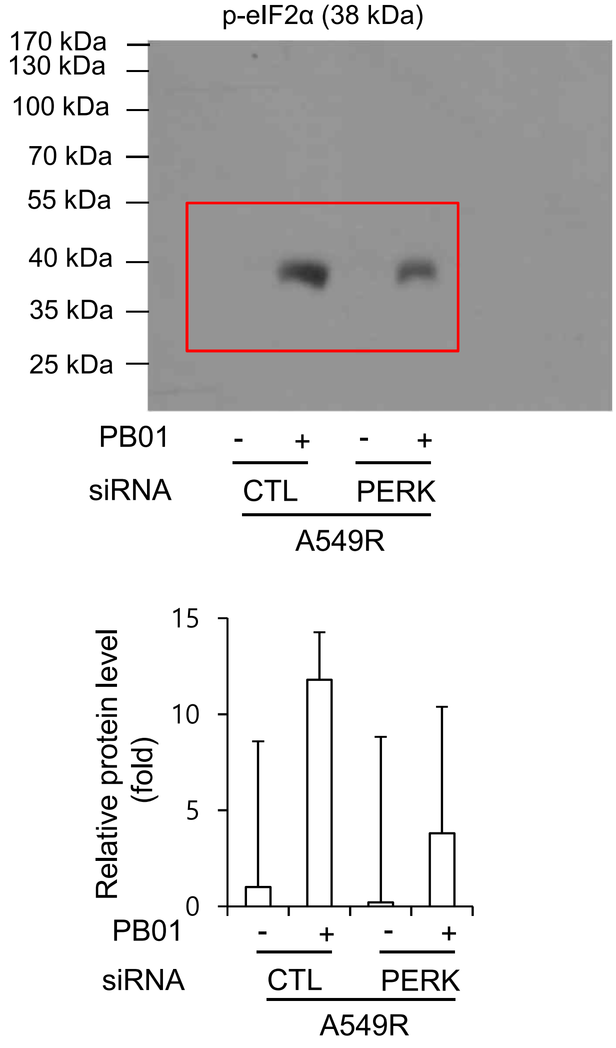

S22-9

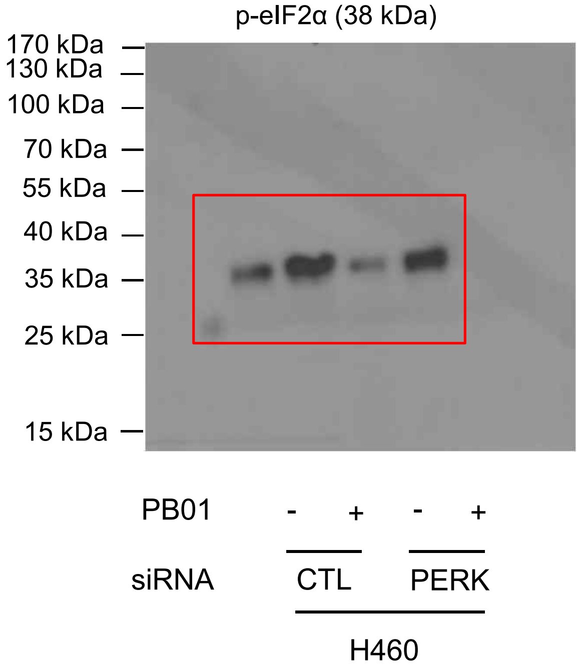

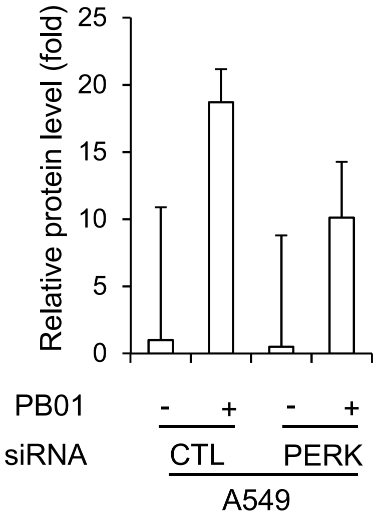

S22-10

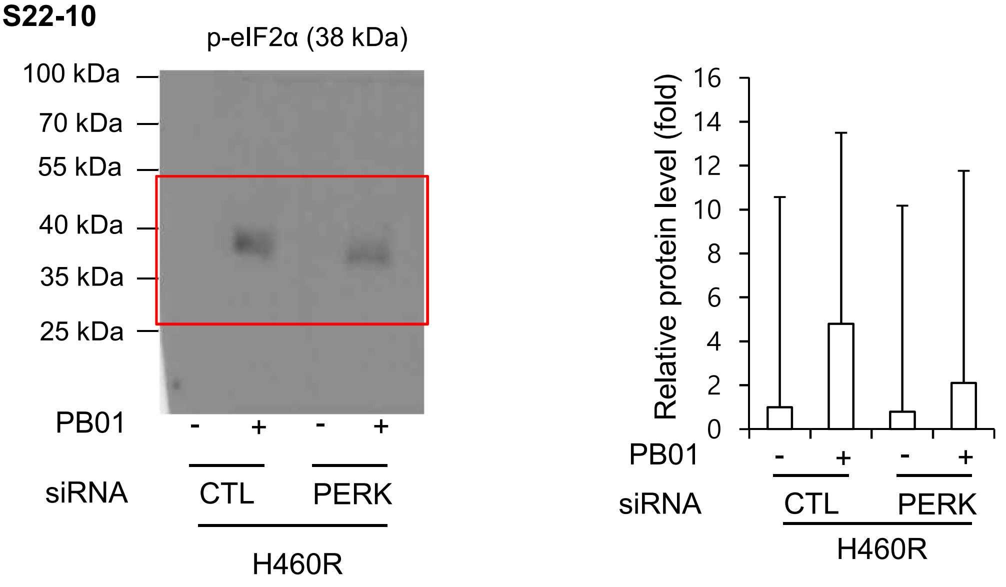

S22-11

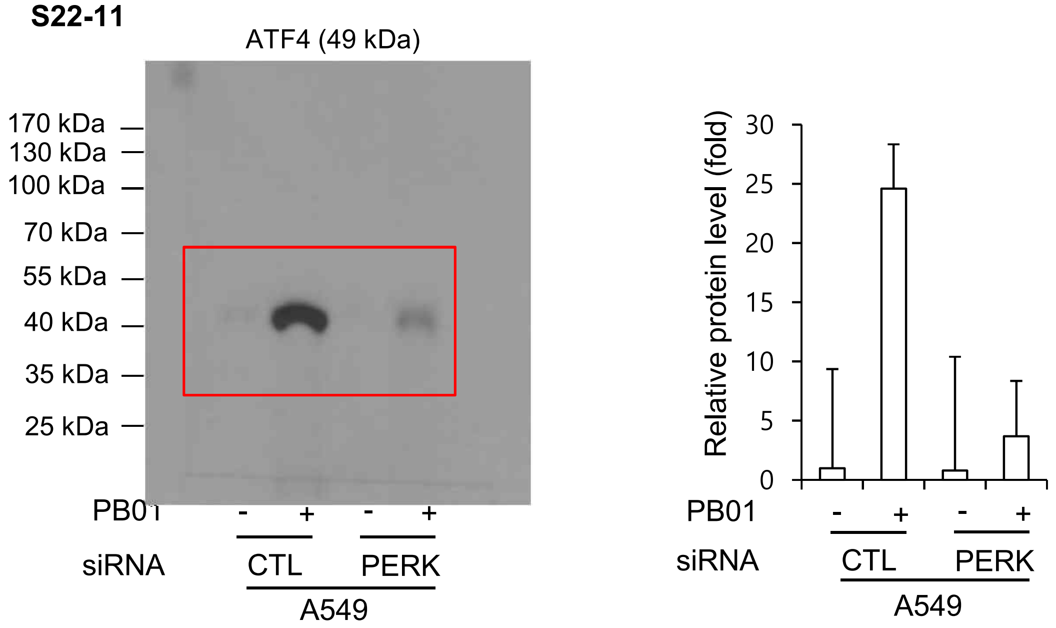

S22-12

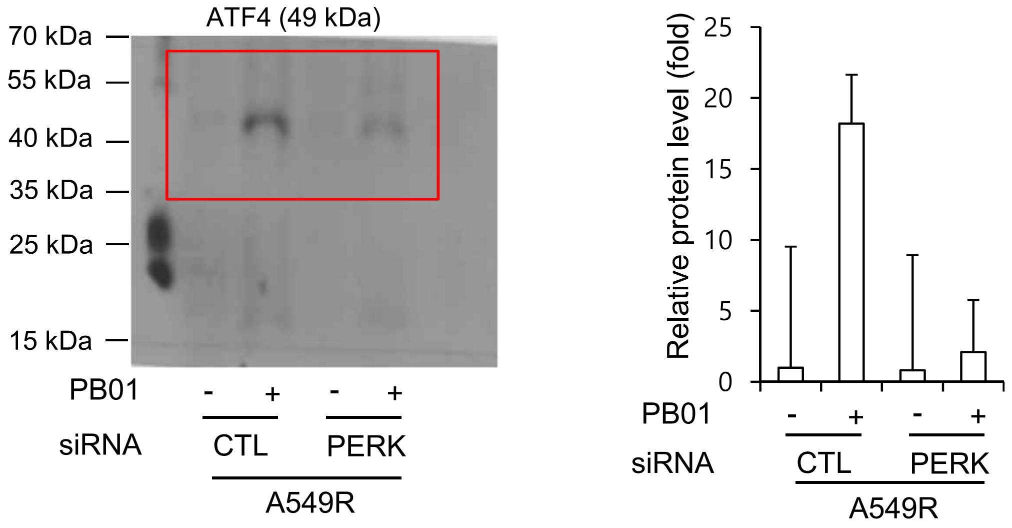

S22-13

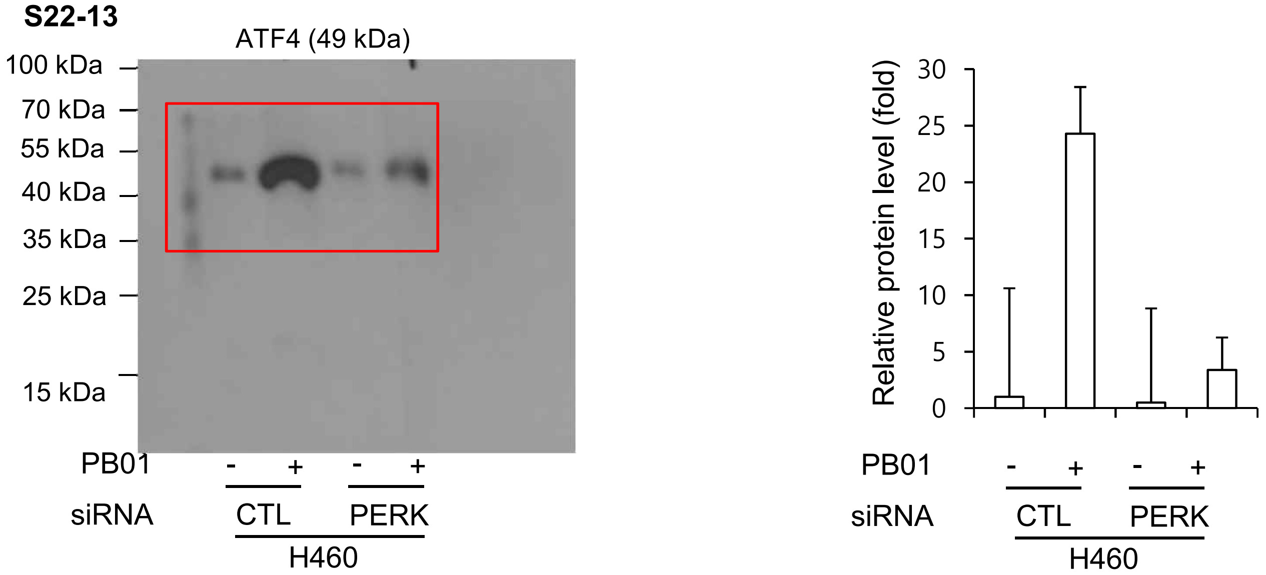

S22-14

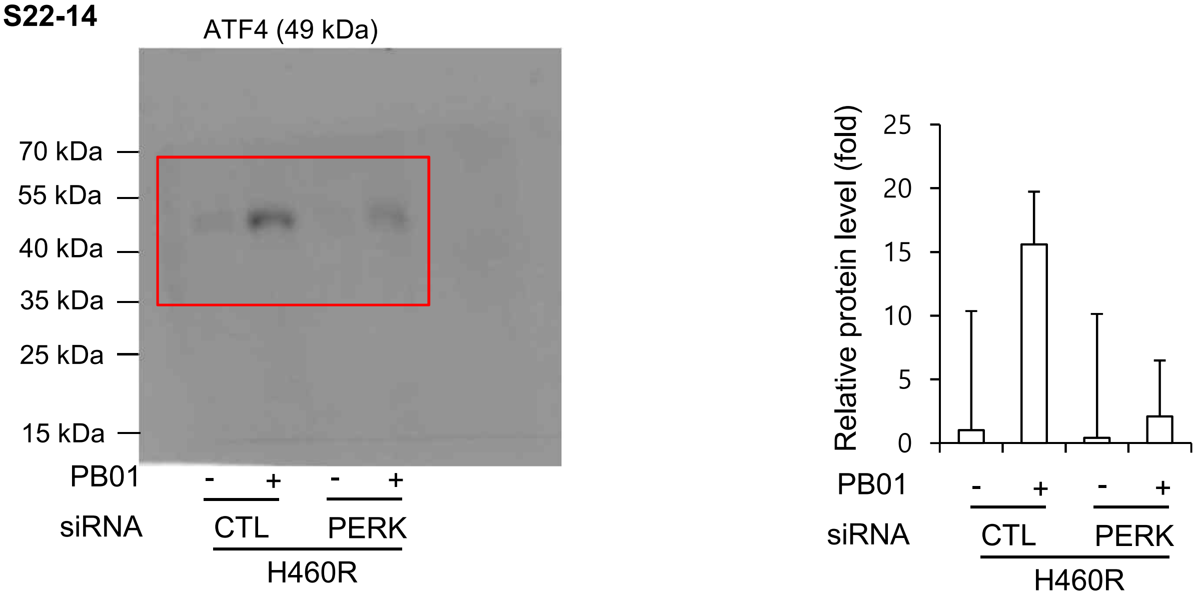

S22-15

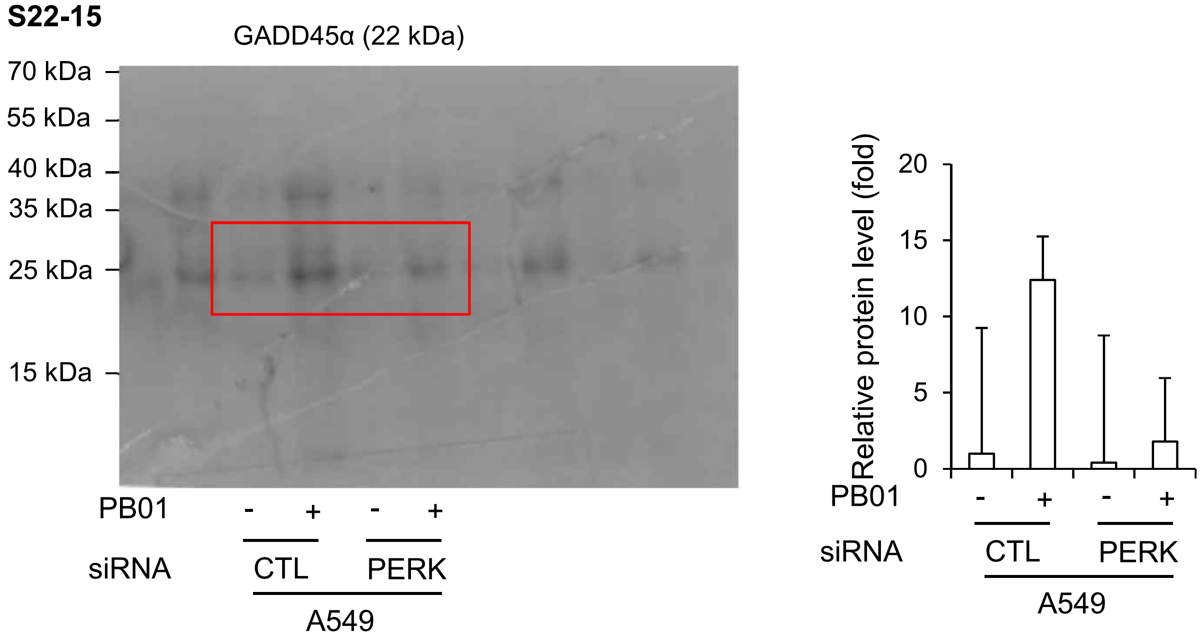

S22-16

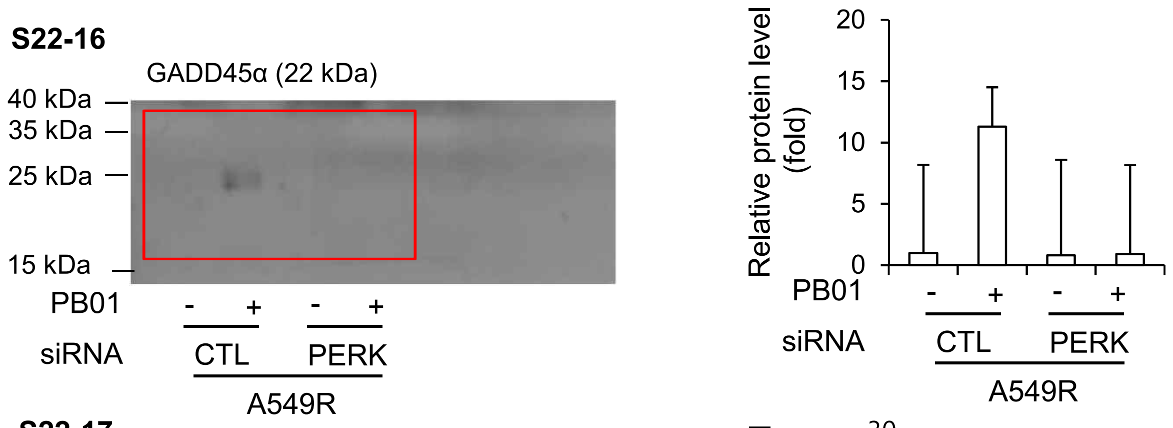

S22-17

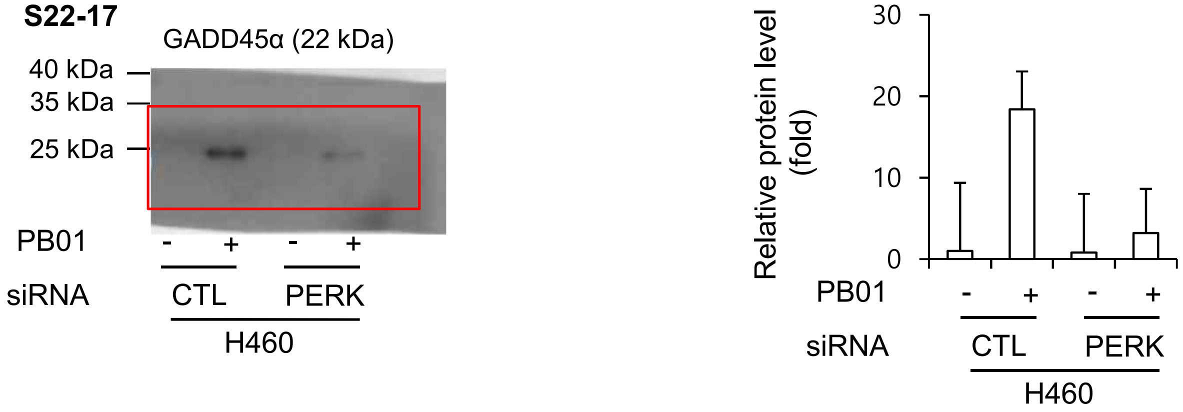

S22-18

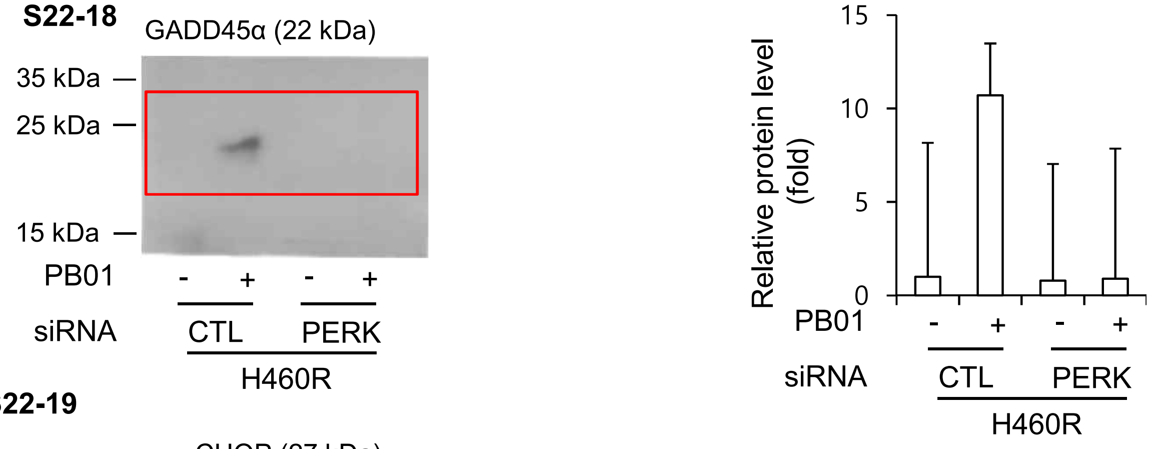

S22-19

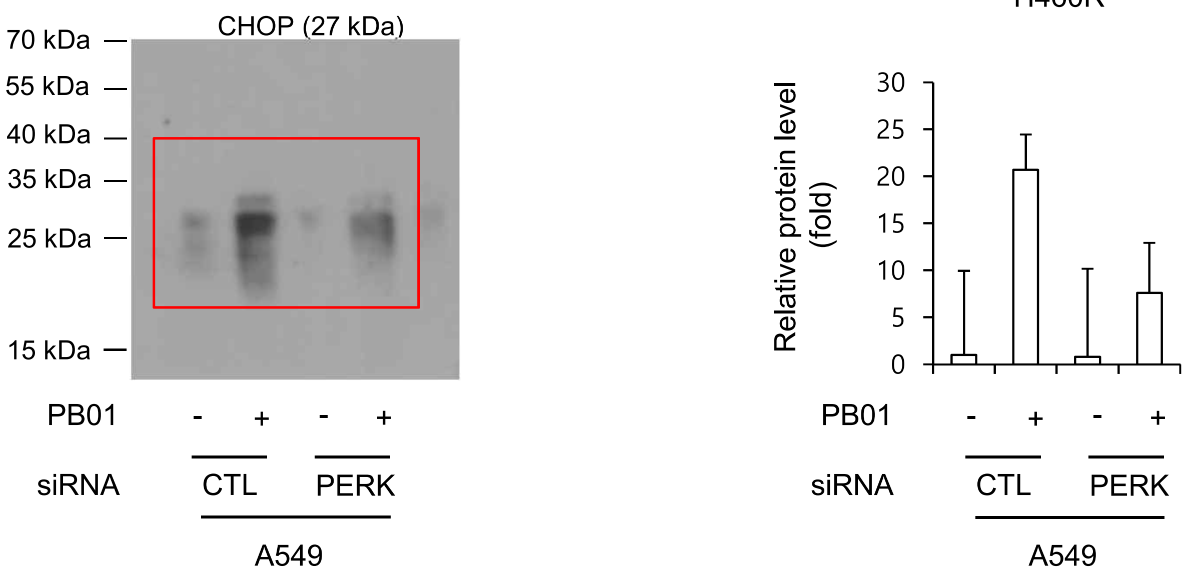

S22-20

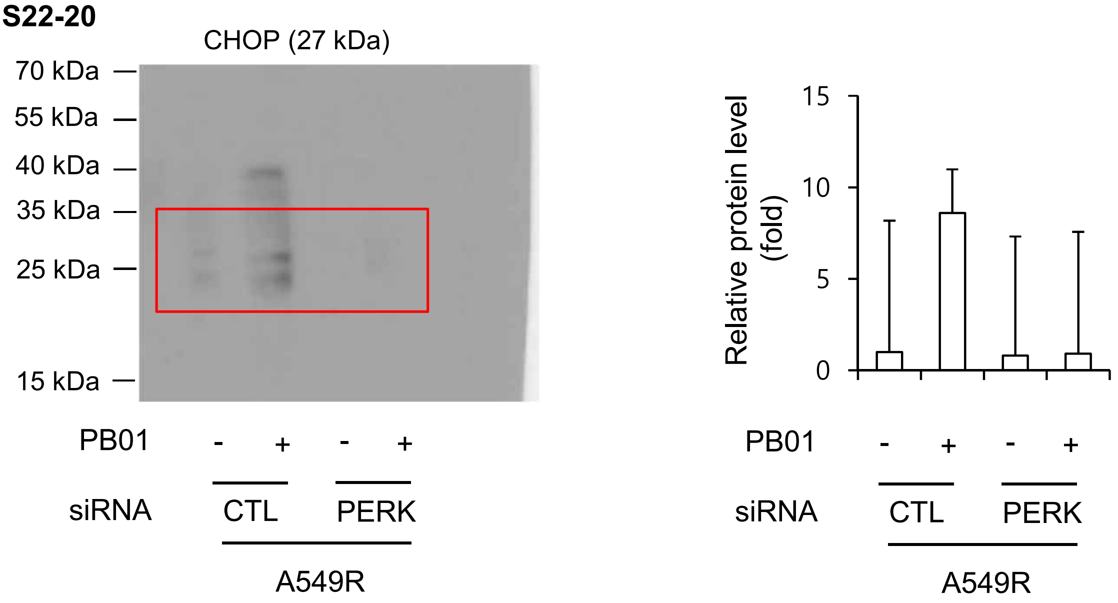

S22-21

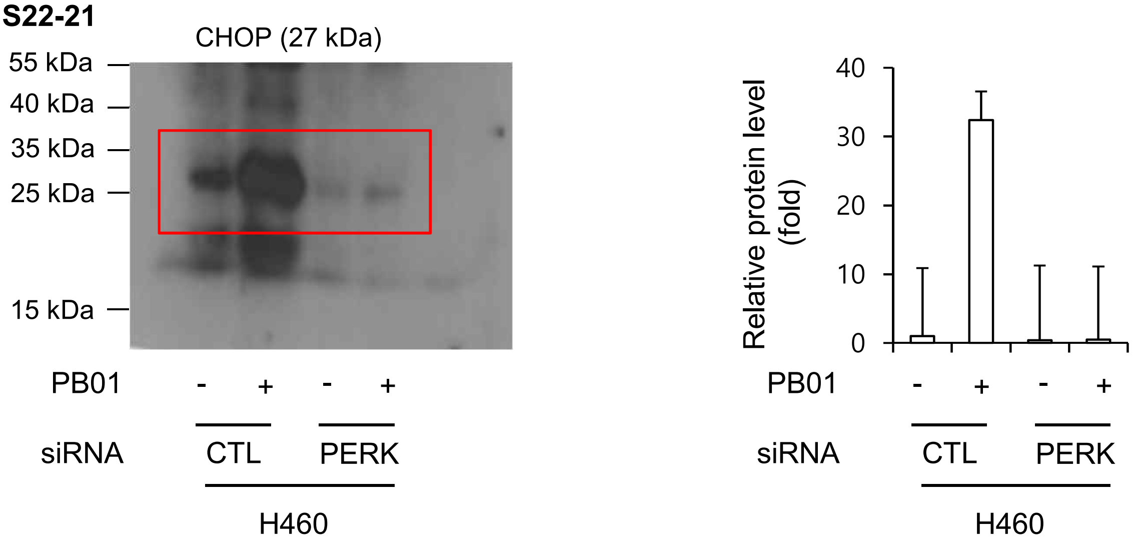

S22-22

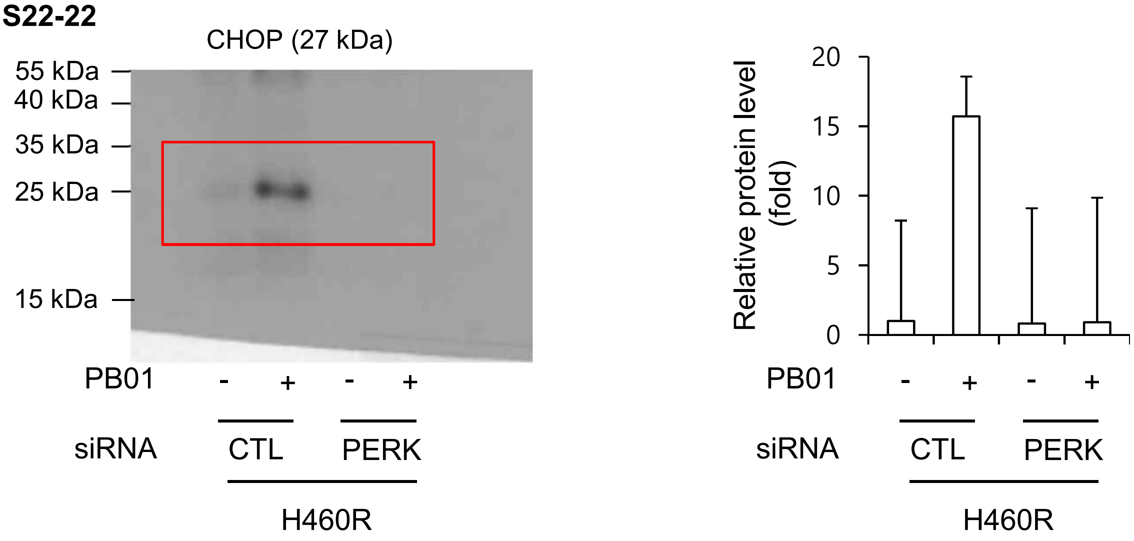

Figure S23. Supplementary materials for Figure 8d

S23-1

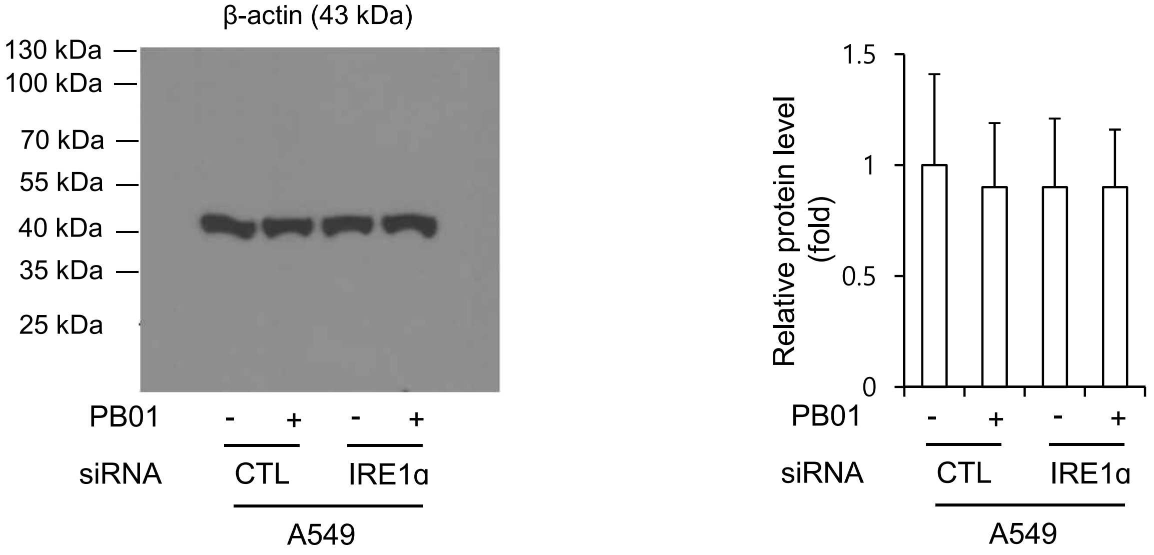

S23-2

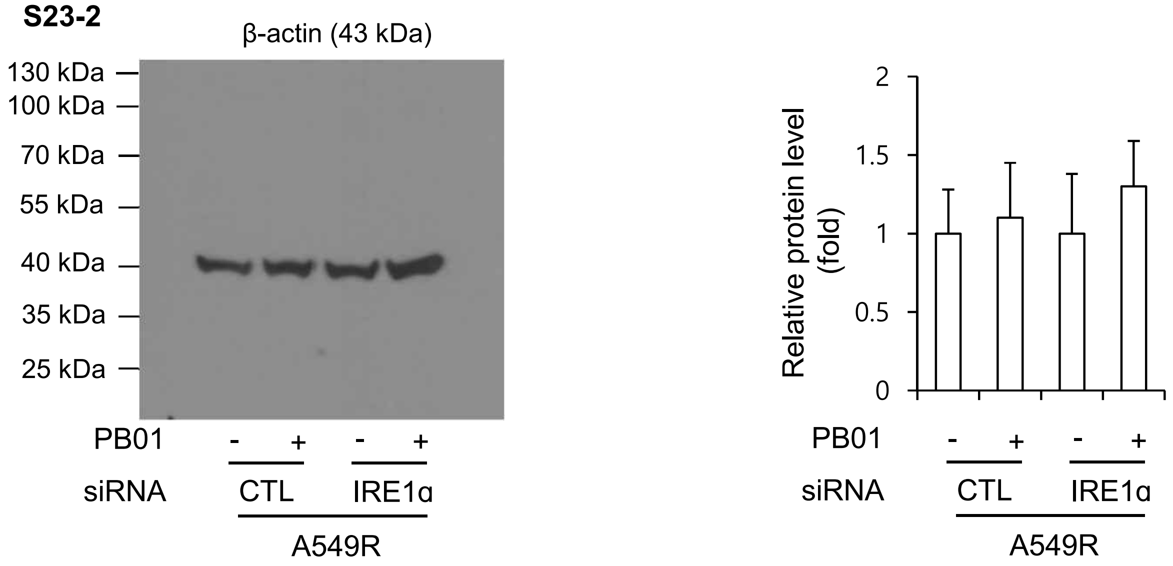

S23-3

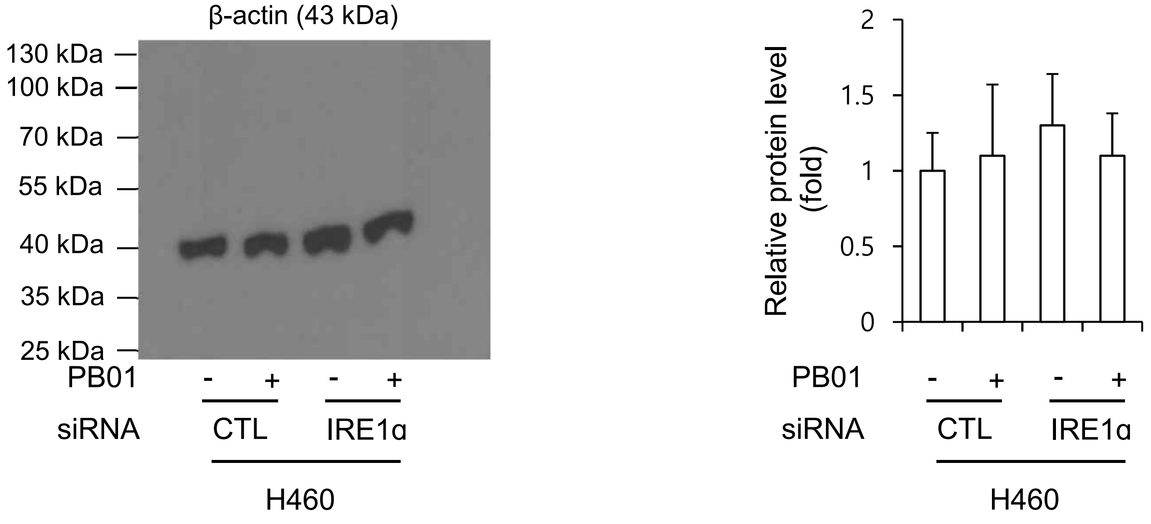

S23-4

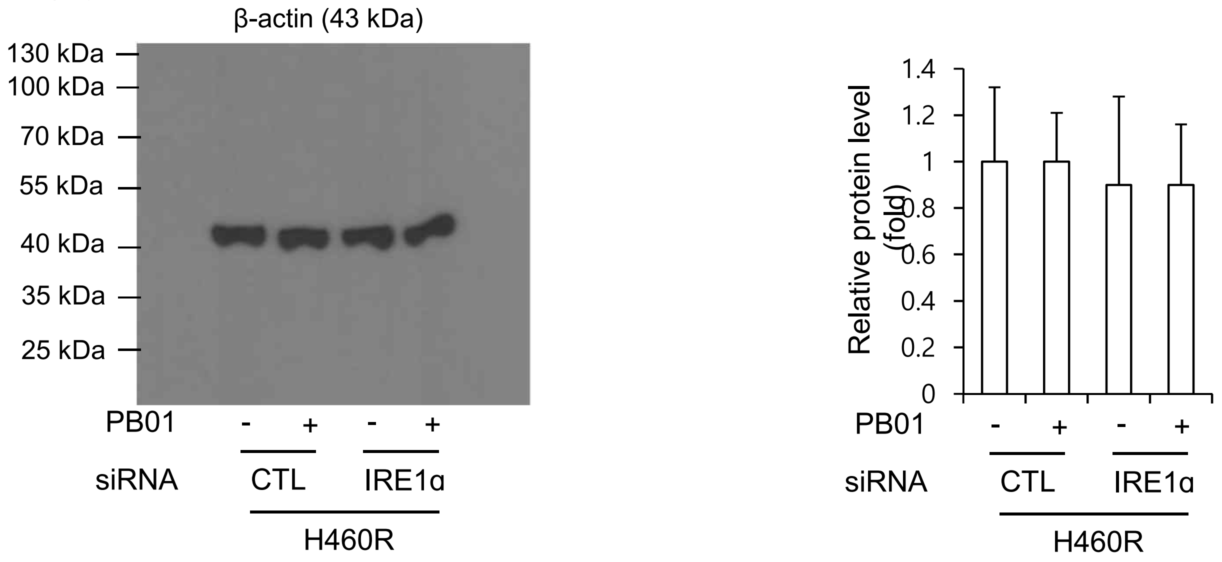

S23-5

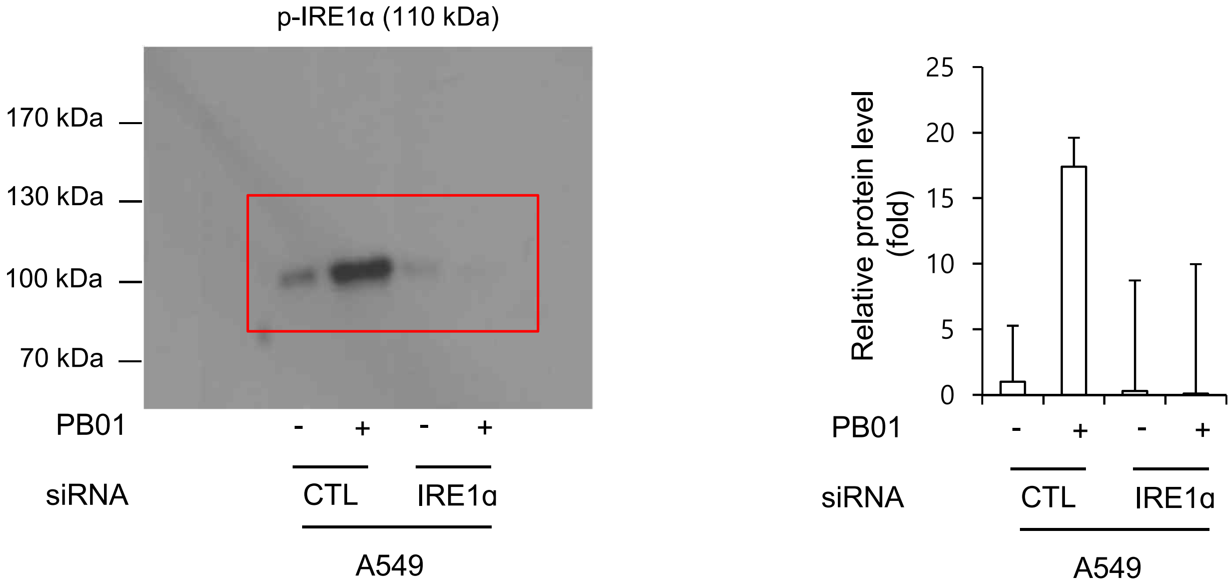

S23-6

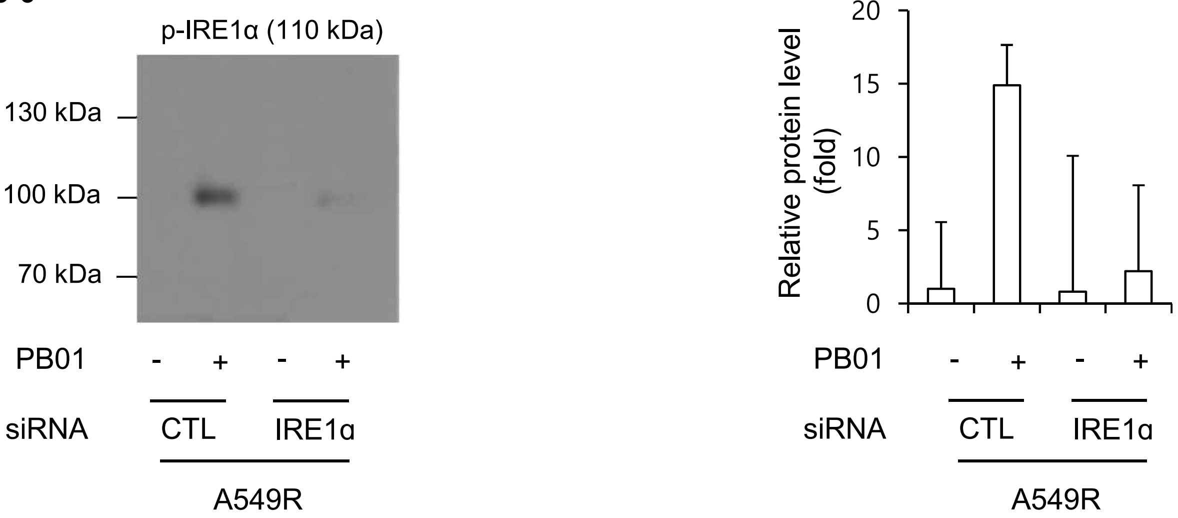

S23-7

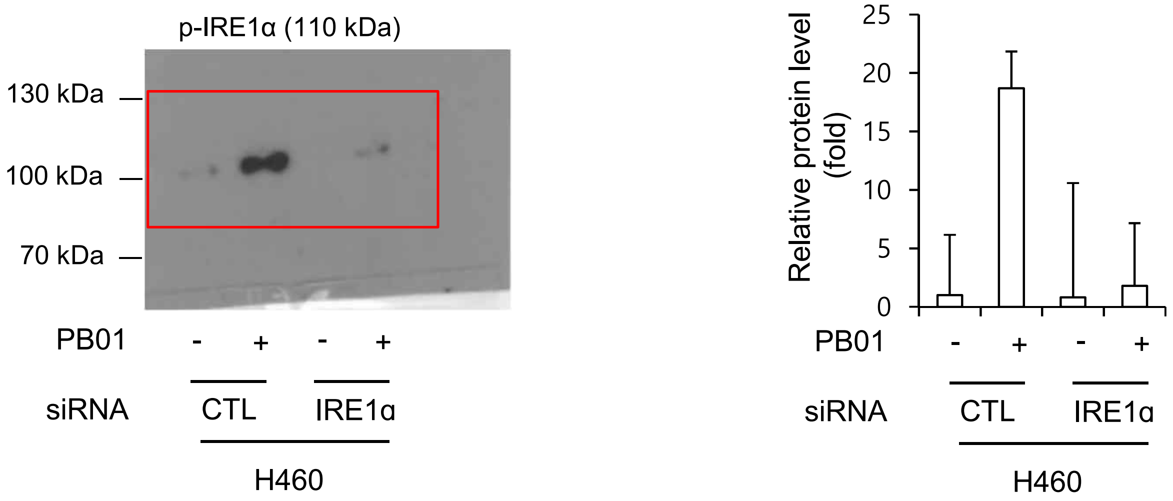

S23-8

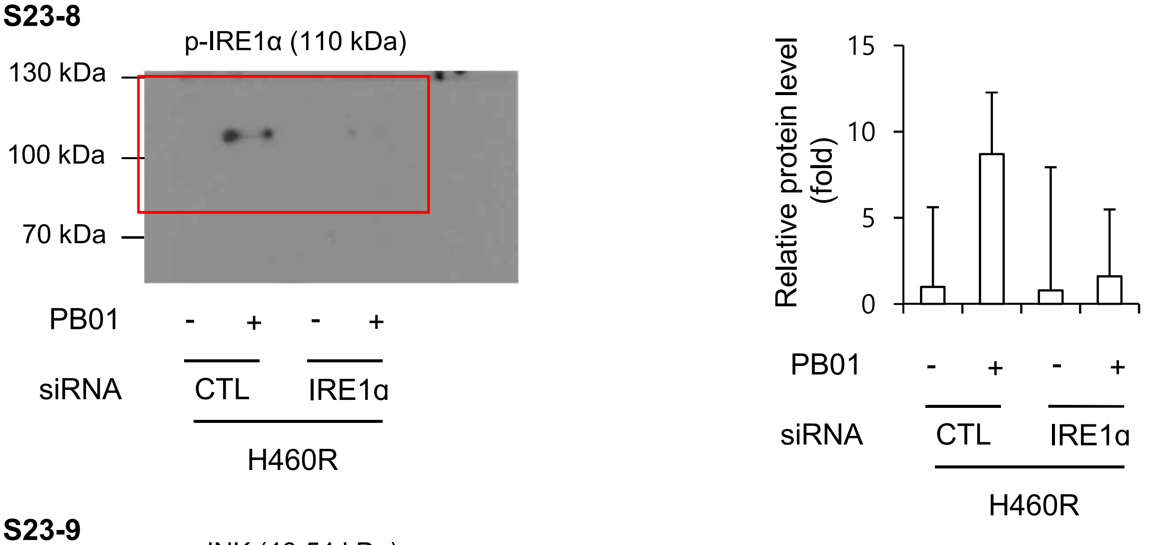

S23-9

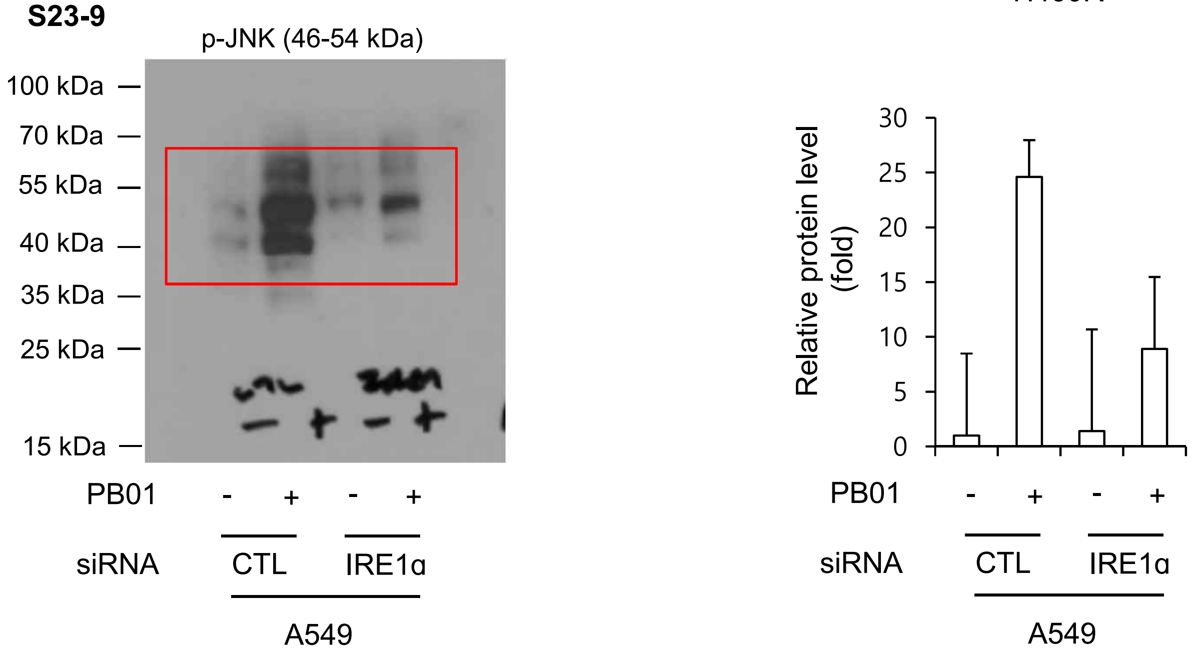

S23-10

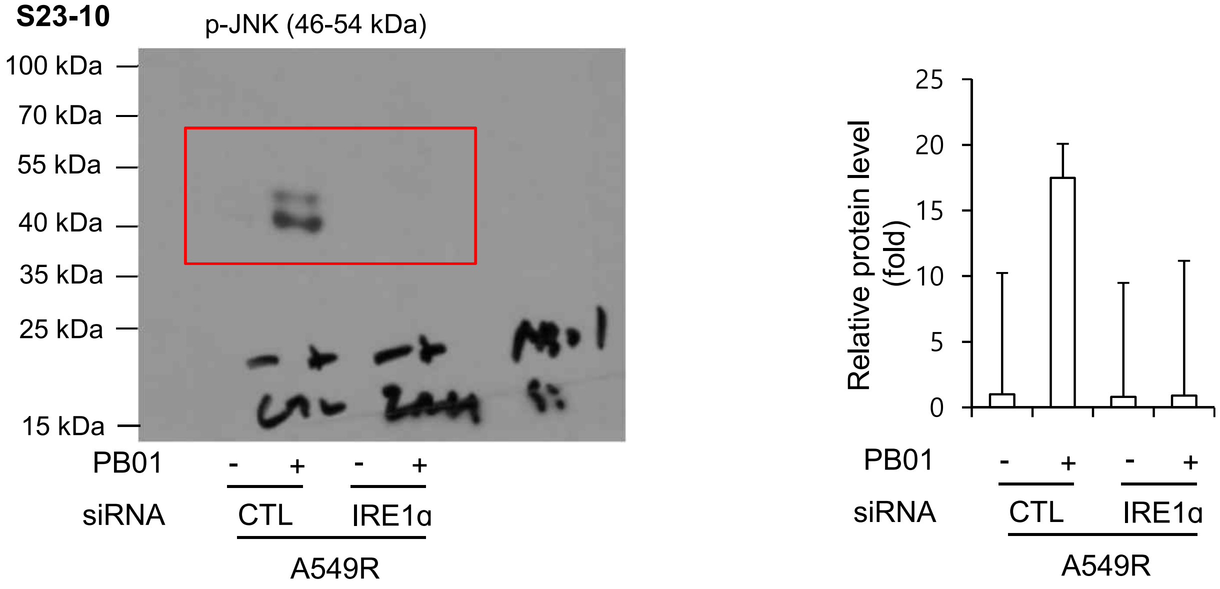

S23-11

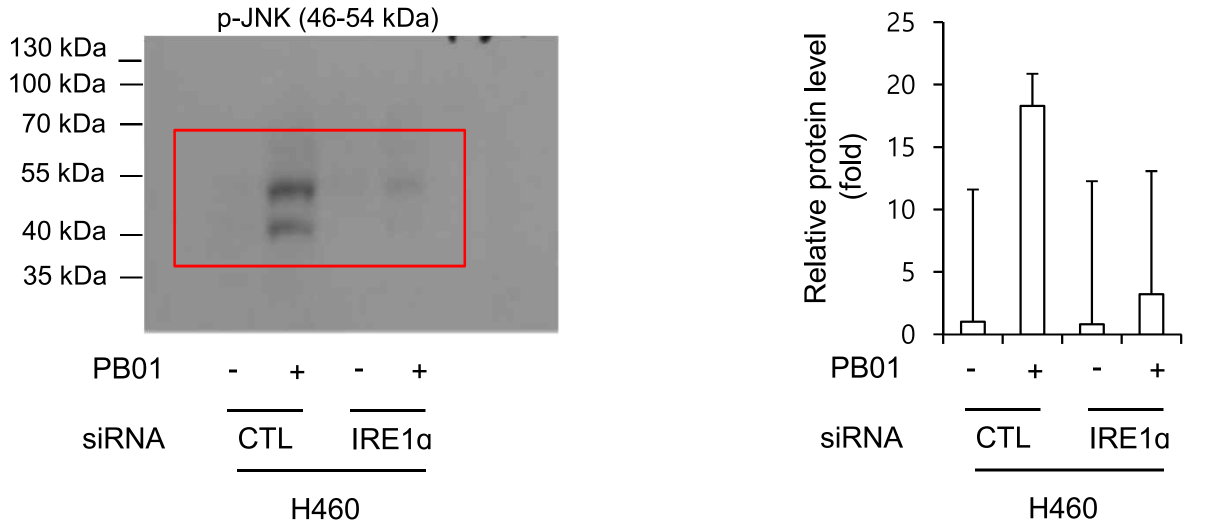

S23-12

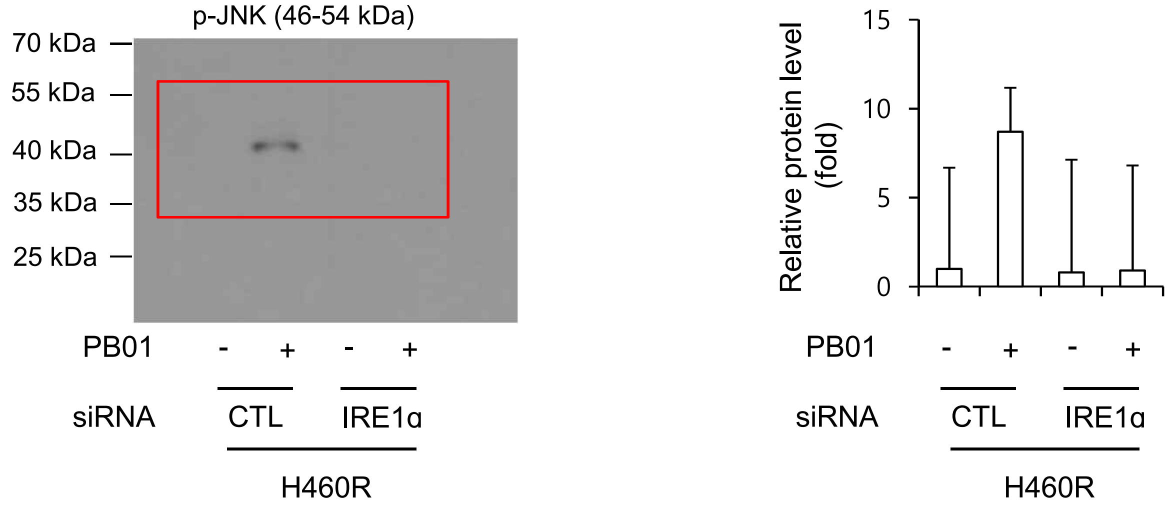

S23-13

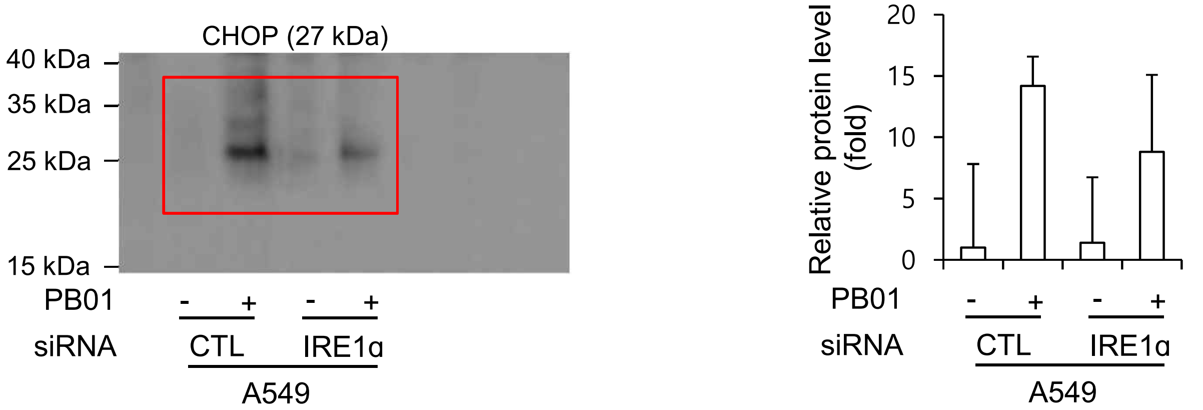

S23-14

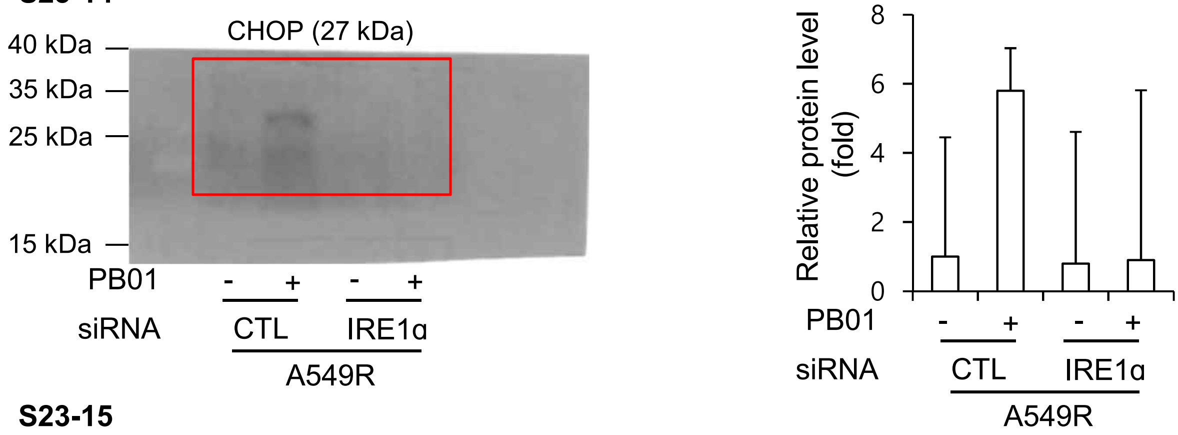

S23-15

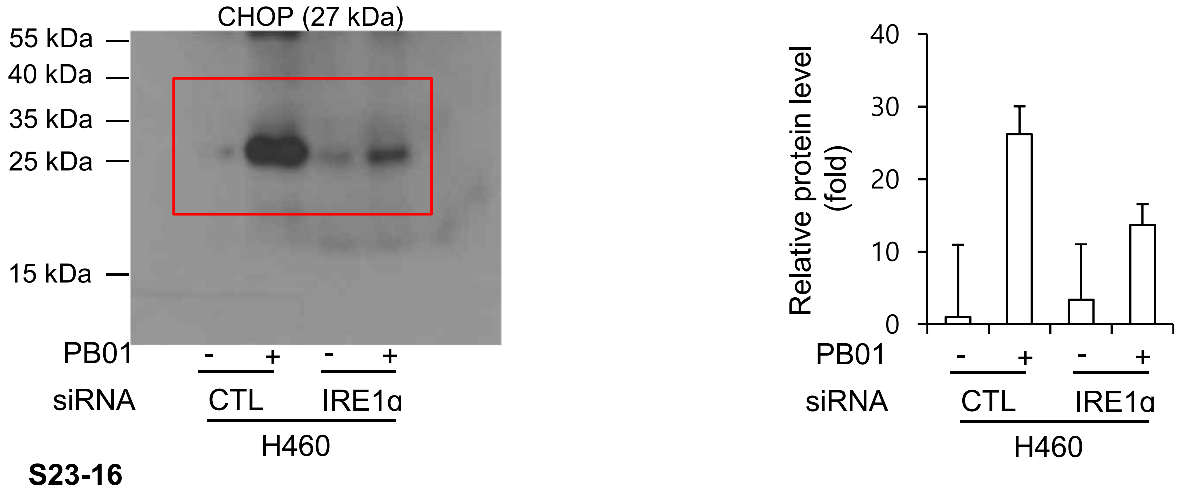

S23-16

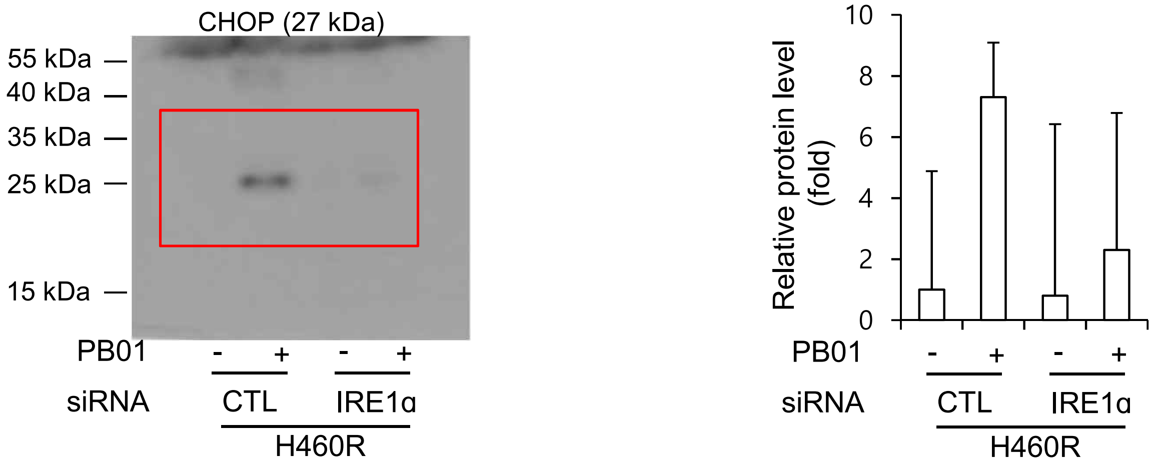

S23-17

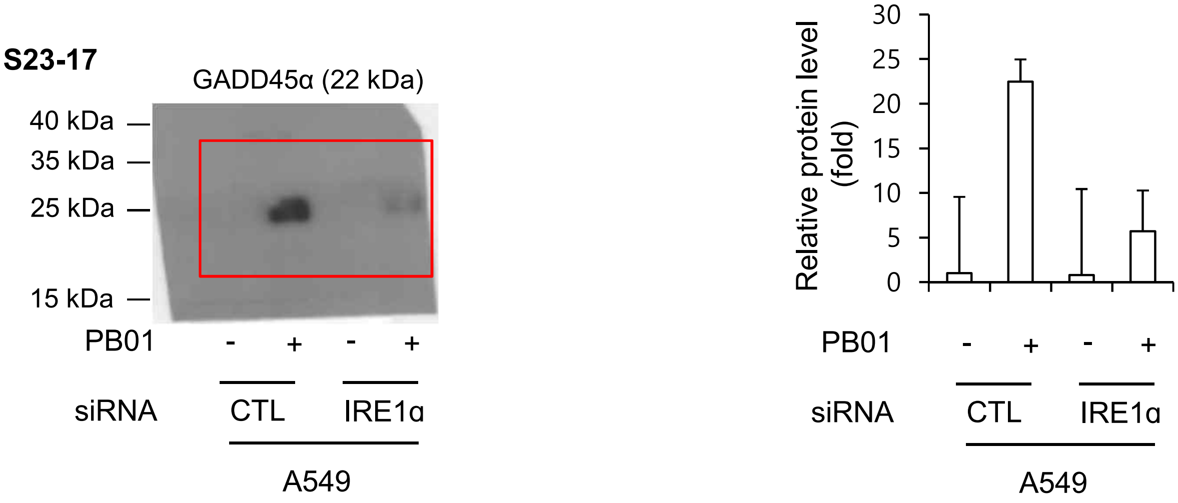

S23-18

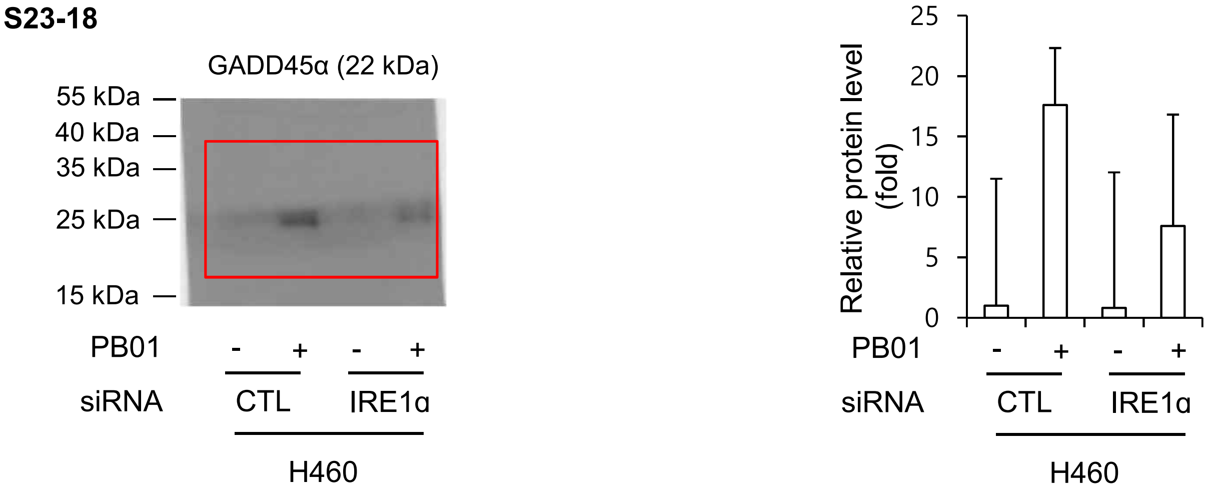

S23-19

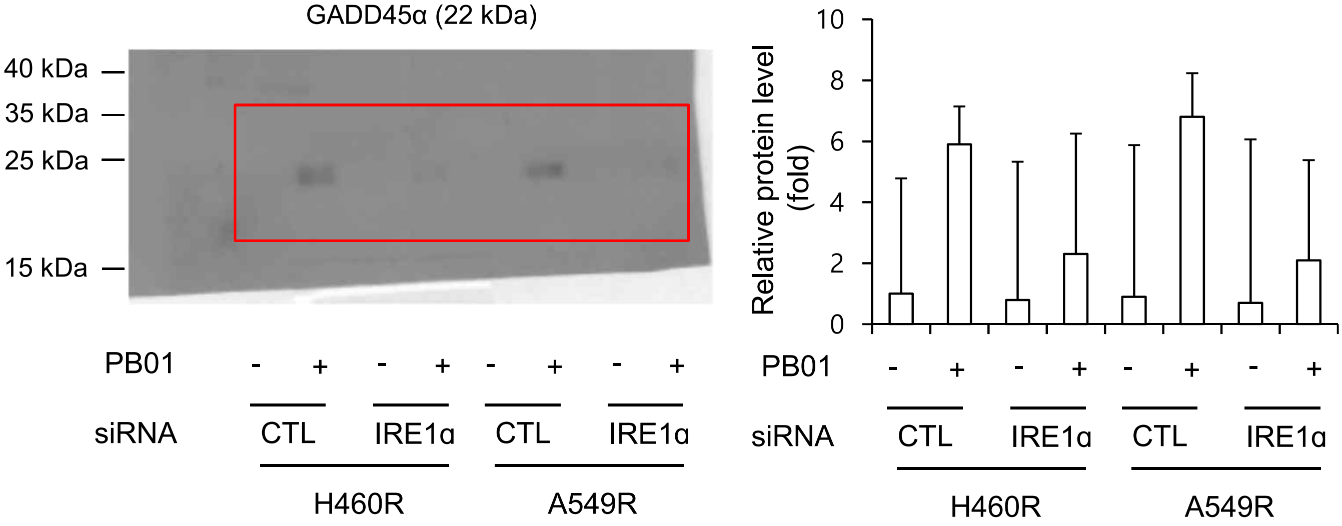

Supplement: Supplementary file 1 — Supplementary Information. [file 41598_2021_91716_MOESM1_ESM.pdf]
